# Supplementary material for: PCanPIE: A group I intron platform for efficient circRNA synthesis at ambient temperatures
Source: RNA. Author manuscript; Available in PMC 2026 Nov 9. (PMC13580324; doi:10.1261/rna.081078.126)
Supplement: Supplementary file 1 [file NIHMS2209016-supplement-Data_source_files.zip › 14. Data source files/Fig. 3 Data/2025_10_22 P6b timecourse/rw 2025_10_22 p6b timecourse t18h-[phosphor]gel_AllLanes.pdf]

Analysis Report: rw 2025_10_22 p6b timecourse t18h-[phosphor].gel

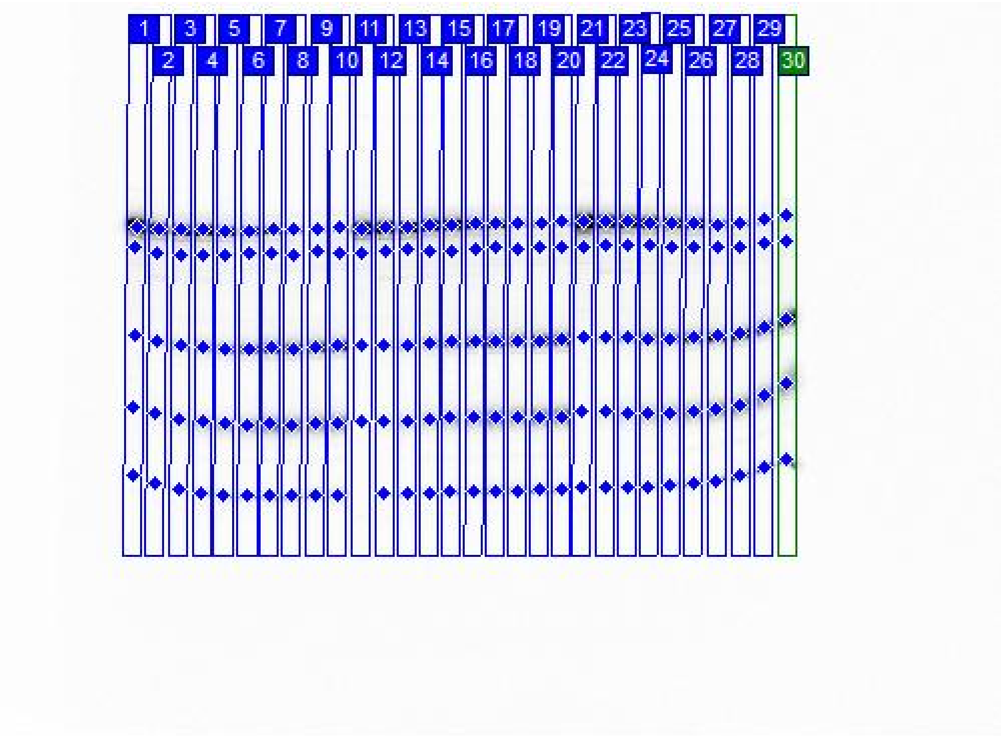

Lane Table

Lane	Bands	Band Volume	Lane Volume
Lane 1	5	1850246.85	2712813.53
Lane 2	5	1376152.99	2014833.16
Lane 3	5	1364767.87	2027291.40
Lane 4	5	1419027.08	2151750.01
Lane 5	5	1222684.25	1855046.58
Lane 6	5	1280039.46	1960762.04
Lane 7	5	1341676.72	2141227.74
Lane 8	5	1112482.18	1799824.73
Lane 9	5	1043309.50	1718035.11
Lane 10	5	1045626.06	1727388.27
Lane 11	4	2009473.71	2806202.04
Lane 12	5	1584092.72	2278046.50
Lane 13	5	1599383.73	2259506.03
Lane 14	5	1505633.36	2279475.03
Lane 15	5	1500406.58	2328533.07
Lane 16	5	1483584.37	2271500.09
Lane 17	5	1532782.57	2456131.91
Lane 18	5	989547.07	1662519.38
Lane 19	5	1213241.61	2001943.91
Lane 20	5	1284706.68	2128653.90
Lane 21	5	2401947.21	3451340.78
Lane 22	5	1607982.93	2366086.08
Lane 23	5	1597266.25	2390009.51
Lane 24	5	1721065.68	2574115.38
Lane 25	5	1294680.25	2045441.40

ImageQuant TL

Lane	Bands	Band Volume	Lane Volume
Lane 26	5	1516322.64	2336376.07
Lane 27	5	1256523.76	2004562.41
Lane 28	5	1112898.57	1799601.32
Lane 29	5	1042925.94	1781033.04
Lane 30	5	1037863.41	2012156.29

ImageQuant TL

Lane Data Report: rw 2025_10_22 p6b timecourse t18h-[phosphor].gel, Lane 1

Summary

**Name:**	Lane 1

Background

**Background Type:**	Image Rectangle
**Rectangle:**	Left 278, Right 928, Top 972, Bottom 1100

Band Detection

**Automatic Detection:**	Yes
**Minimum Band Slope:**	184
**Median Filter:**	5
**Percentage Maximum Peak:**	0
**Maximum Peak Measure:**	Gel
**Edge Detection Method:**	Fixed Width
**Fixed Width pixels:**	17
**Band Positions Edited:**	Yes

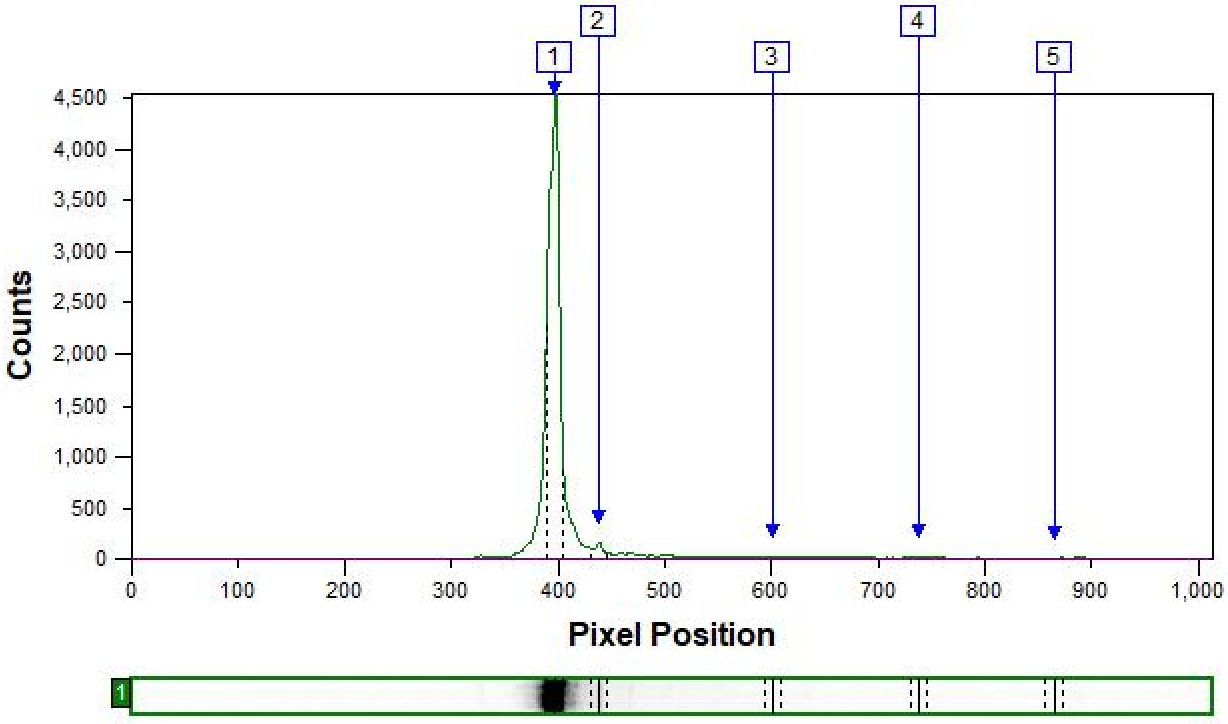

Band Table

Band No	Volume	Vol+BkGnd	Band %
1	1778746.49	1785608.43	96.136
2	56286.67	63150.34	3.042
3	7906.65	14772.50	0.427
4	4749.52	11616.84	0.257
5	2557.52	9426.74	0.138

ImageQuant TL

Lane Data Report: rw 2025_10_22 p6b timecourse t18h-[phosphor].gel, Lane 2

Summary

**Name:**	Lane 2

Background

**Background Type:**	Image Rectangle
**Rectangle:**	Left 278, Right 928, Top 972, Bottom 1100

Band Detection

**Automatic Detection:**	Yes
**Minimum Band Slope:**	184
**Median Filter:**	5
**Percentage Maximum Peak:**	0
**Maximum Peak Measure:**	Gel
**Edge Detection Method:**	Fixed Width
**Fixed Width pixels:**	17
**Band Positions Edited:**	Yes

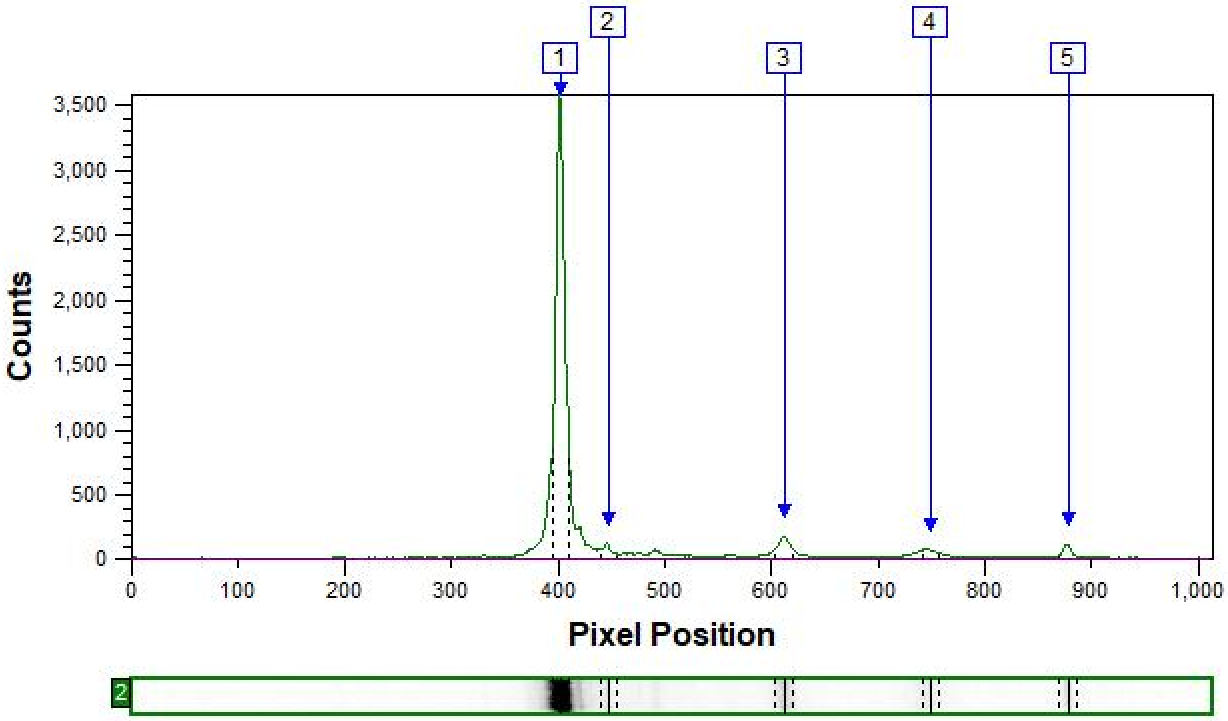

Band Table

Band No	Volume	Vol+BkGnd	Band %
1	1202850.88	1209714.74	87.407
2	38977.35	45838.85	2.832
3	68499.29	75367.30	4.978
4	33088.52	39966.87	2.404
5	32736.96	39598.59	2.379

ImageQuant TL

Lane Data Report: rw 2025_10_22 p6b timecourse t18h-[phosphor].gel, Lane 3

Summary

**Name:**	Lane 3

Background

**Background Type:**	Image Rectangle
**Rectangle:**	Left 278, Right 928, Top 972, Bottom 1100

Band Detection

**Automatic Detection:**	Yes
**Minimum Band Slope:**	184
**Median Filter:**	5
**Percentage Maximum Peak:**	0
**Maximum Peak Measure:**	Gel
**Edge Detection Method:**	Fixed Width
**Fixed Width pixels:**	17
**Band Positions Edited:**	Yes

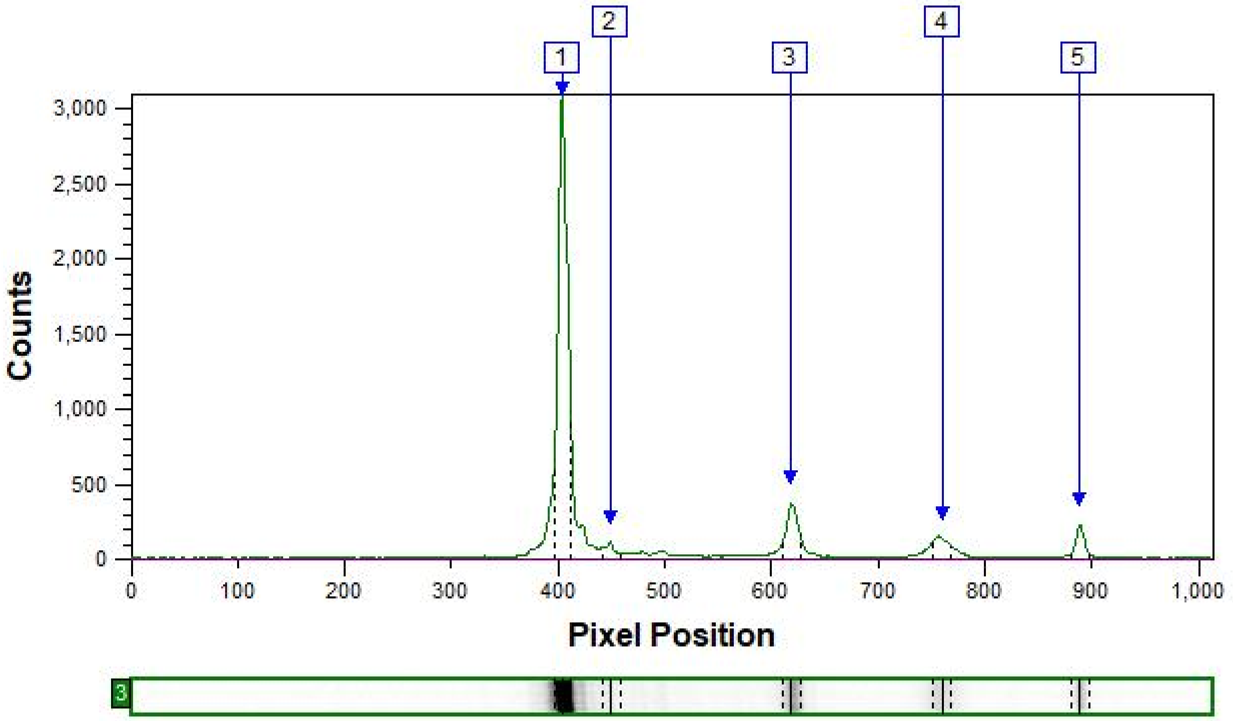

Band Table

Band No	Volume	Vol+BkGnd	Band %
1	1051327.24	1058189.41	77.033
2	38016.16	44880.09	2.786
3	139624.99	146487.88	10.231
4	67816.80	74680.45	4.969
5	67982.68	74848.22	4.981

Lane Data Report: rw 2025_10_22 p6b timecourse t18h-[phosphor].gel, Lane 4

Summary

**Name:**	Lane 4

Background

**Background Type:**	Image Rectangle
**Rectangle:**	Left 278, Right 928, Top 972, Bottom 1100

Band Detection

**Automatic Detection:**	Yes
**Minimum Band Slope:**	184
**Median Filter:**	5
**Percentage Maximum Peak:**	0
**Maximum Peak Measure:**	Gel
**Edge Detection Method:**	Fixed Width
**Fixed Width pixels:**	17
**Band Positions Edited:**	Yes

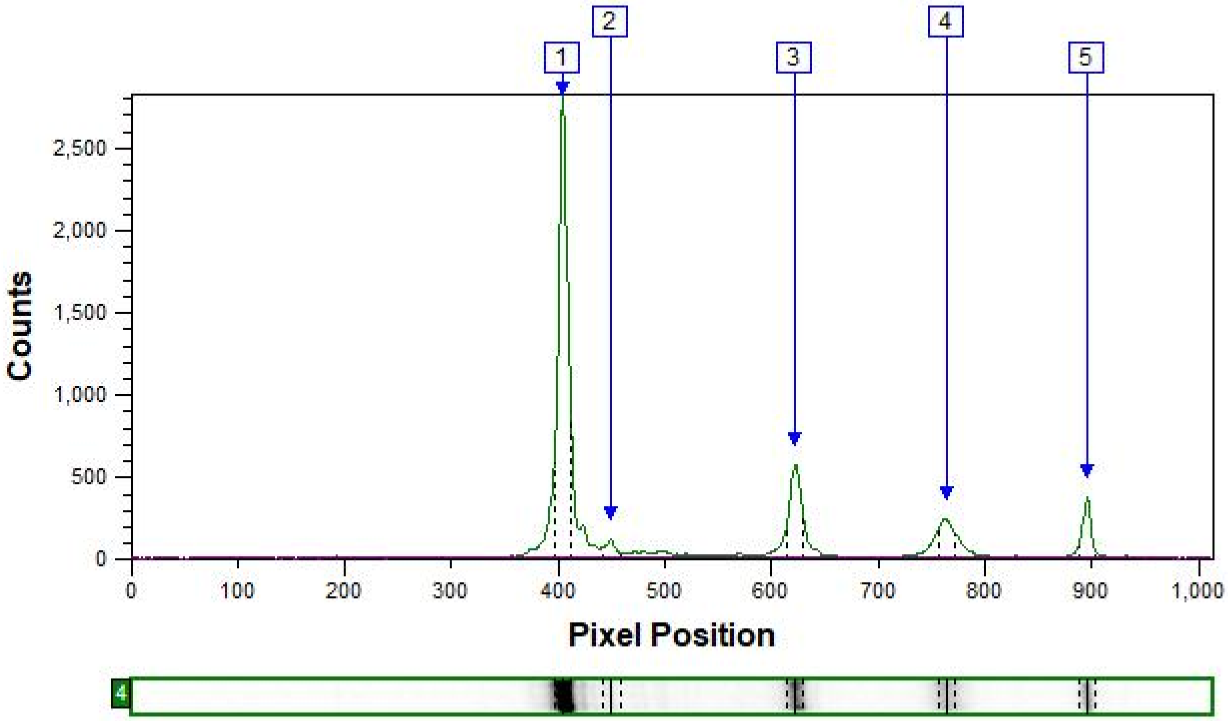

Band Table

Band No	Volume	Vol+BkGnd	Band %
1	930676.84	937539.14	65.586
2	40620.97	47485.27	2.863
3	223405.58	230268.71	15.744
4	113356.29	120219.59	7.988
5	110967.40	117835.98	7.820

Lane Data Report: rw 2025_10_22 p6b timecourse t18h-[phosphor].gel, Lane 5

Summary

**Name:**	Lane 5

Background

**Background Type:**	Image Rectangle
**Rectangle:**	Left 278, Right 928, Top 972, Bottom 1100

Band Detection

**Automatic Detection:**	Yes
**Minimum Band Slope:**	184
**Median Filter:**	5
**Percentage Maximum Peak:**	0
**Maximum Peak Measure:**	Gel
**Edge Detection Method:**	Fixed Width
**Fixed Width pixels:**	17
**Band Positions Edited:**	Yes

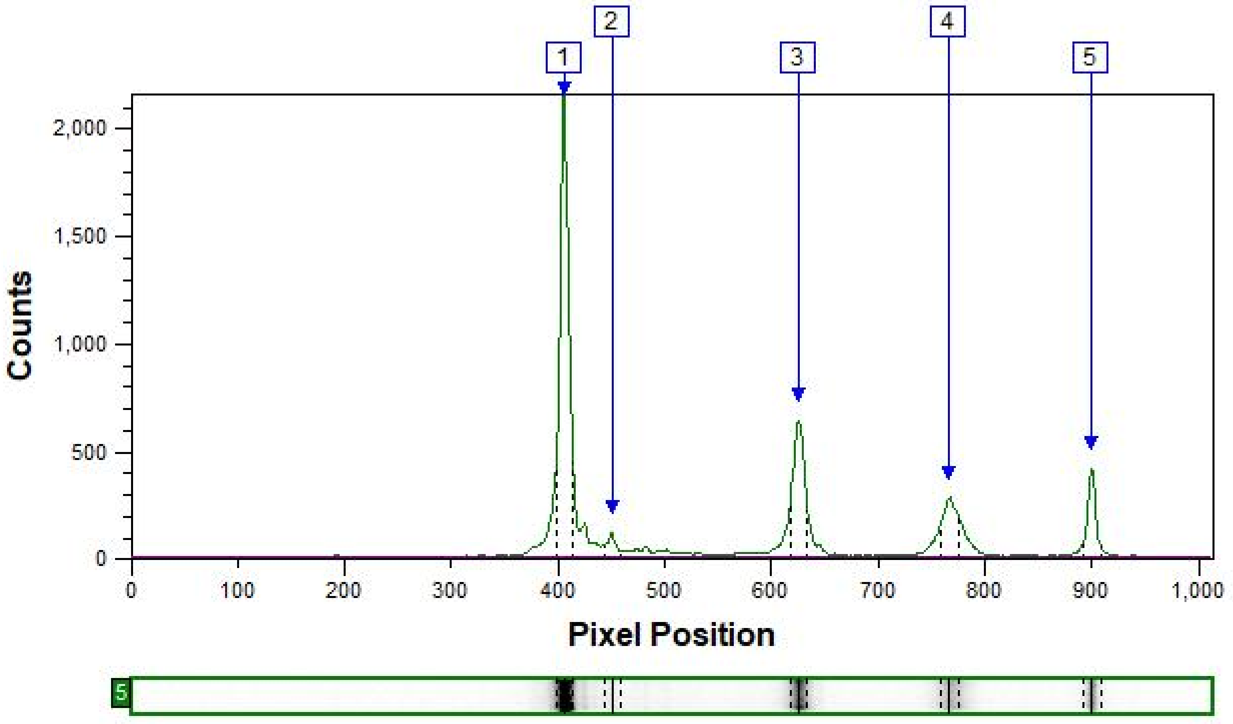

Band Table

Band No	Volume	Vol+BkGnd	Band %
1	675531.44	682397.41	55.250
2	35949.18	42817.05	2.940
3	260768.44	267635.00	21.328
4	124717.07	131583.90	10.200
5	125718.12	132569.80	10.282

ImageQuant TL

Lane Data Report: rw 2025_10_22 p6b timecourse t18h-[phosphor].gel, Lane 6

Summary

**Name:**	Lane 6

Background

**Background Type:**	Image Rectangle
**Rectangle:**	Left 278, Right 928, Top 972, Bottom 1100

Band Detection

**Automatic Detection:**	Yes
**Minimum Band Slope:**	184
**Median Filter:**	5
**Percentage Maximum Peak:**	0
**Maximum Peak Measure:**	Gel
**Edge Detection Method:**	Fixed Width
**Fixed Width pixels:**	17
**Band Positions Edited:**	Yes

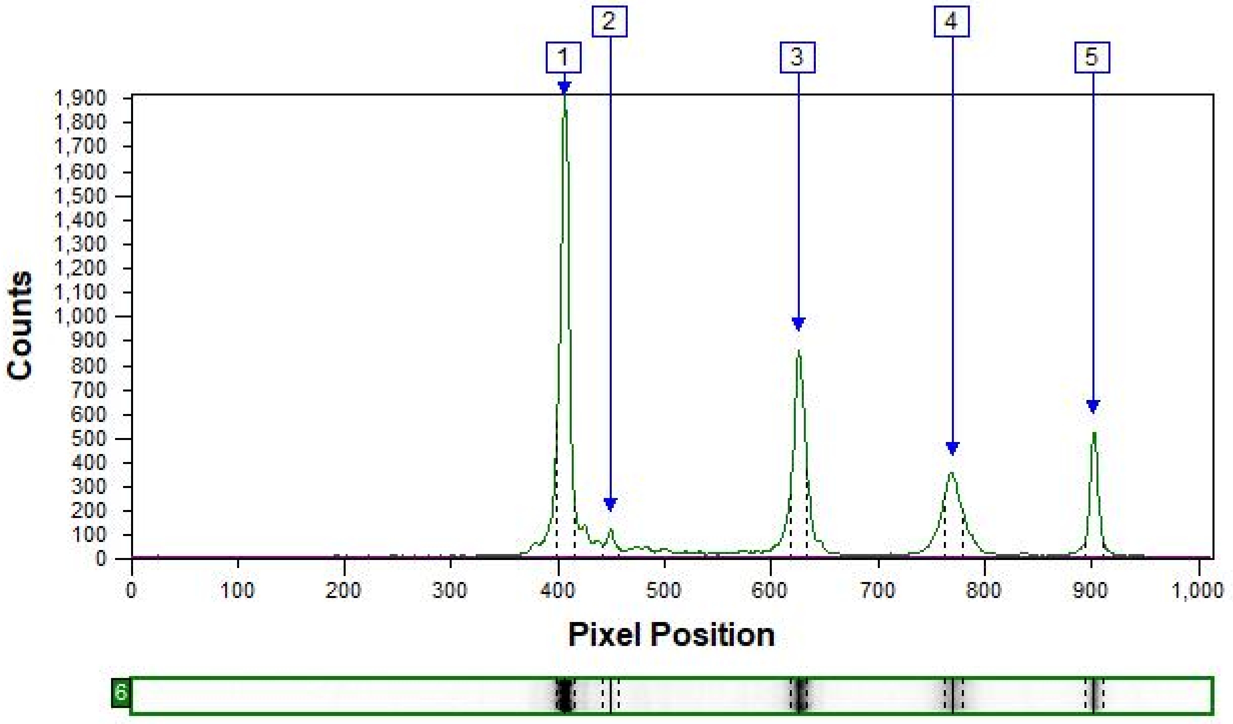

Band Table

Band No	Volume	Vol+BkGnd	Band %
1	593892.85	600754.23	46.396
2	37694.11	44555.50	2.945
3	329567.35	336428.73	25.747
4	159231.12	166092.50	12.440
5	159654.03	166515.41	12.473

Lane Data Report: rw 2025_10_22 p6b timecourse t18h-[phosphor].gel, Lane 7

Summary

**Name:**	Lane 7

Background

**Background Type:**	Image Rectangle
**Rectangle:**	Left 278, Right 928, Top 972, Bottom 1100

Band Detection

**Automatic Detection:**	Yes
**Minimum Band Slope:**	184
**Median Filter:**	5
**Percentage Maximum Peak:**	0
**Maximum Peak Measure:**	Gel
**Edge Detection Method:**	Fixed Width
**Fixed Width pixels:**	17
**Band Positions Edited:**	Yes

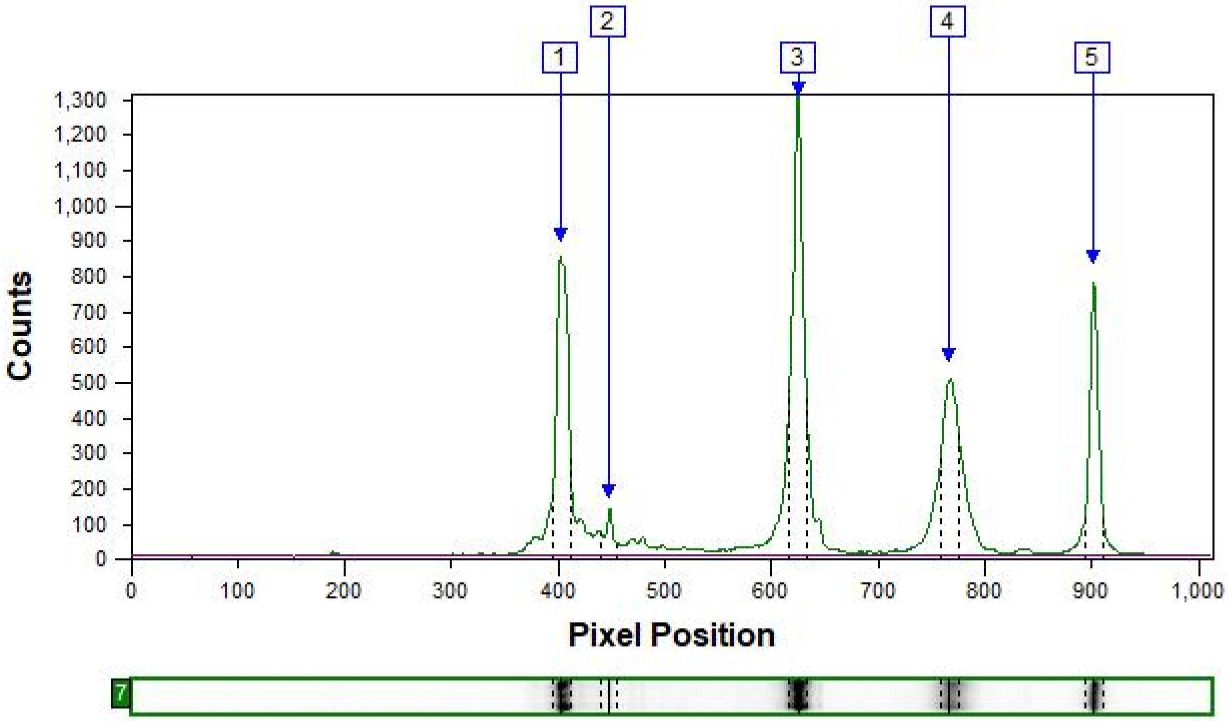

Band Table

Band No	Volume	Vol+BkGnd	Band %
1	339799.41	346660.79	25.326
2	40050.82	46912.20	2.985
3	480885.34	487746.72	35.842
4	235307.79	242169.18	17.538
5	245633.35	252494.73	18.308

ImageQuant TL

Lane Data Report: rw 2025_10_22 p6b timecourse t18h-[phosphor].gel, Lane 8

Summary

**Name:**	Lane 8

Background

**Background Type:**	Image Rectangle
**Rectangle:**	Left 278, Right 928, Top 972, Bottom 1100

Band Detection

**Automatic Detection:**	Yes
**Minimum Band Slope:**	184
**Median Filter:**	5
**Percentage Maximum Peak:**	0
**Maximum Peak Measure:**	Gel
**Edge Detection Method:**	Fixed Width
**Fixed Width pixels:**	17
**Band Positions Edited:**	Yes

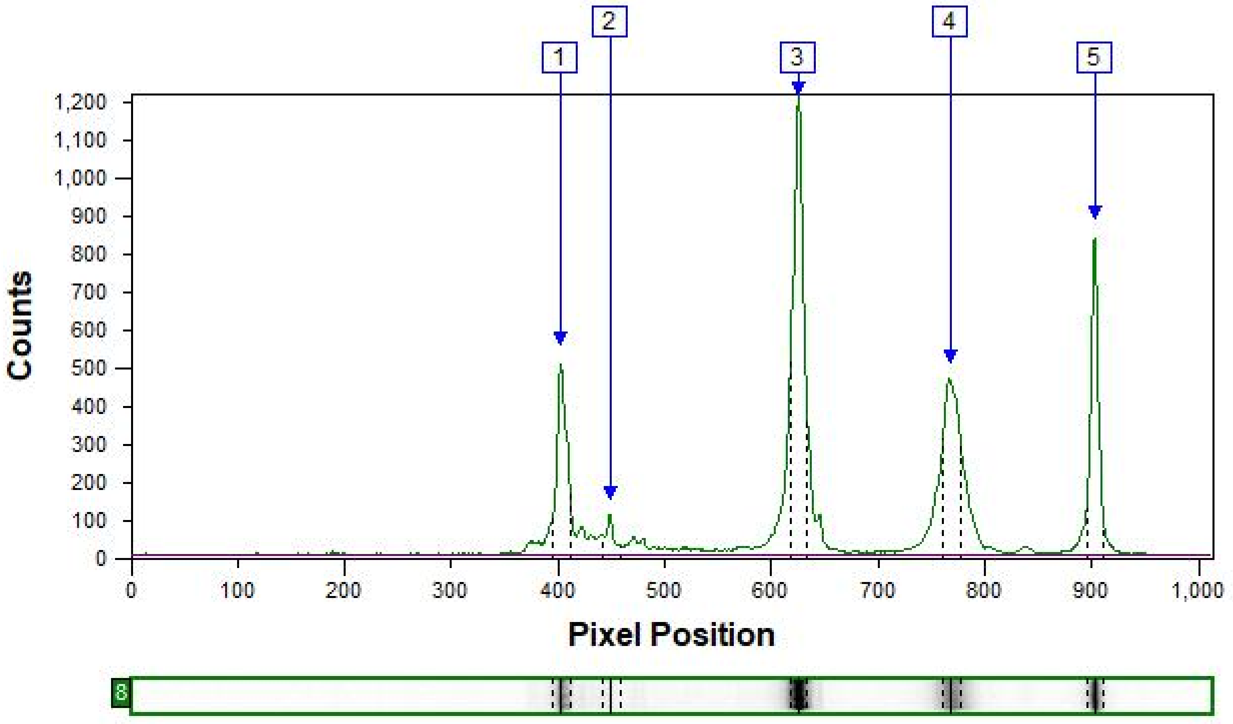

Band Table

Band No	Volume	Vol+BkGnd	Band %
1	173092.34	179953.72	15.559
2	31010.73	37872.12	2.788
3	452250.10	459111.48	40.652
4	221917.99	228779.37	19.948
5	234211.03	241072.41	21.053

ImageQuant TL

Lane Data Report: rw 2025_10_22 p6b timecourse t18h-[phosphor].gel, Lane 9

Summary

**Name:**	Lane 9

Background

**Background Type:**	Image Rectangle
**Rectangle:**	Left 278, Right 928, Top 972, Bottom 1100

Band Detection

**Automatic Detection:**	Yes
**Minimum Band Slope:**	184
**Median Filter:**	5
**Percentage Maximum Peak:**	0
**Maximum Peak Measure:**	Gel
**Edge Detection Method:**	Fixed Width
**Fixed Width pixels:**	17
**Band Positions Edited:**	Yes

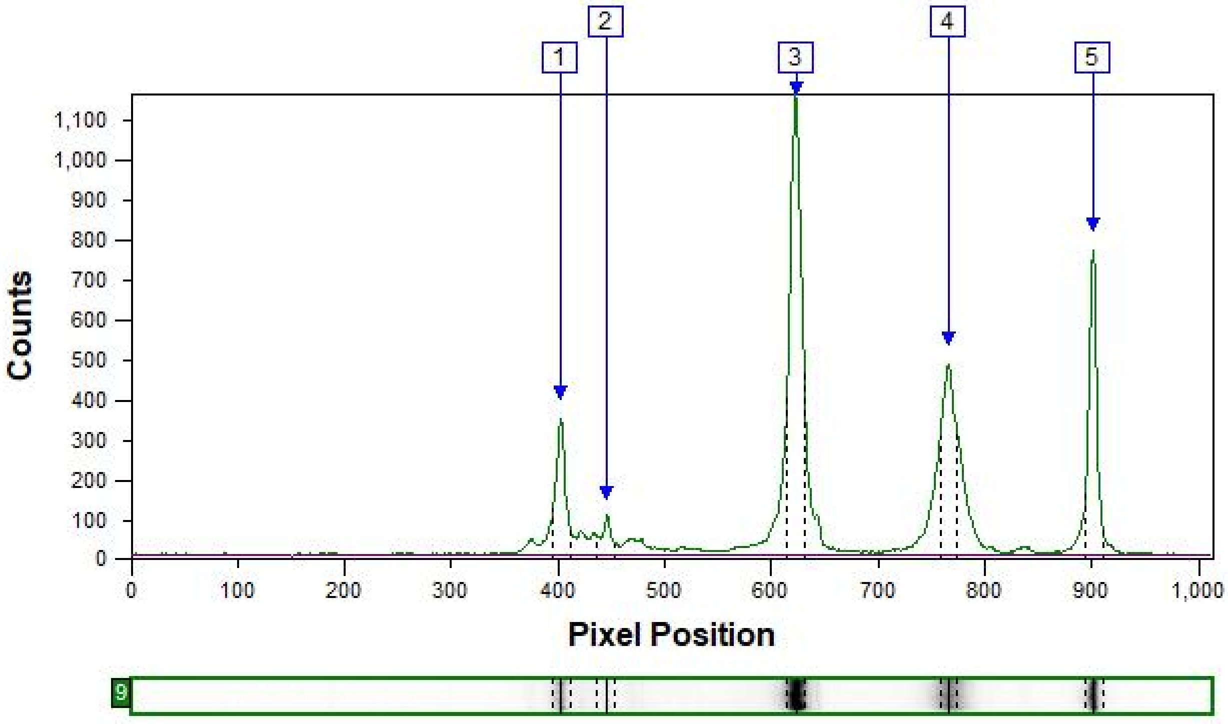

Band Table

Band No	Volume	Vol+BkGnd	Band %
1	116536.50	123397.88	11.170
2	32553.76	39415.15	3.120
3	449020.98	455882.36	43.038
4	216324.54	223185.92	20.734
5	228873.72	235735.11	21.937

IQTL Report version 8.1
Time of Report: 3:31 PM 10/22/2025 (Eastern Daylight Time)

ImageQuant TL

Lane Data Report: rw 2025_10_22 p6b timecourse t18h-[phosphor].gel, Lane 10

Summary

**Name:**	Lane 10

Background

**Background Type:**	Image Rectangle
**Rectangle:**	Left 278, Right 928, Top 972, Bottom 1100

Band Detection

**Automatic Detection:**	Yes
**Minimum Band Slope:**	184
**Median Filter:**	5
**Percentage Maximum Peak:**	0
**Maximum Peak Measure:**	Gel
**Edge Detection Method:**	Fixed Width
**Fixed Width pixels:**	17
**Band Positions Edited:**	Yes

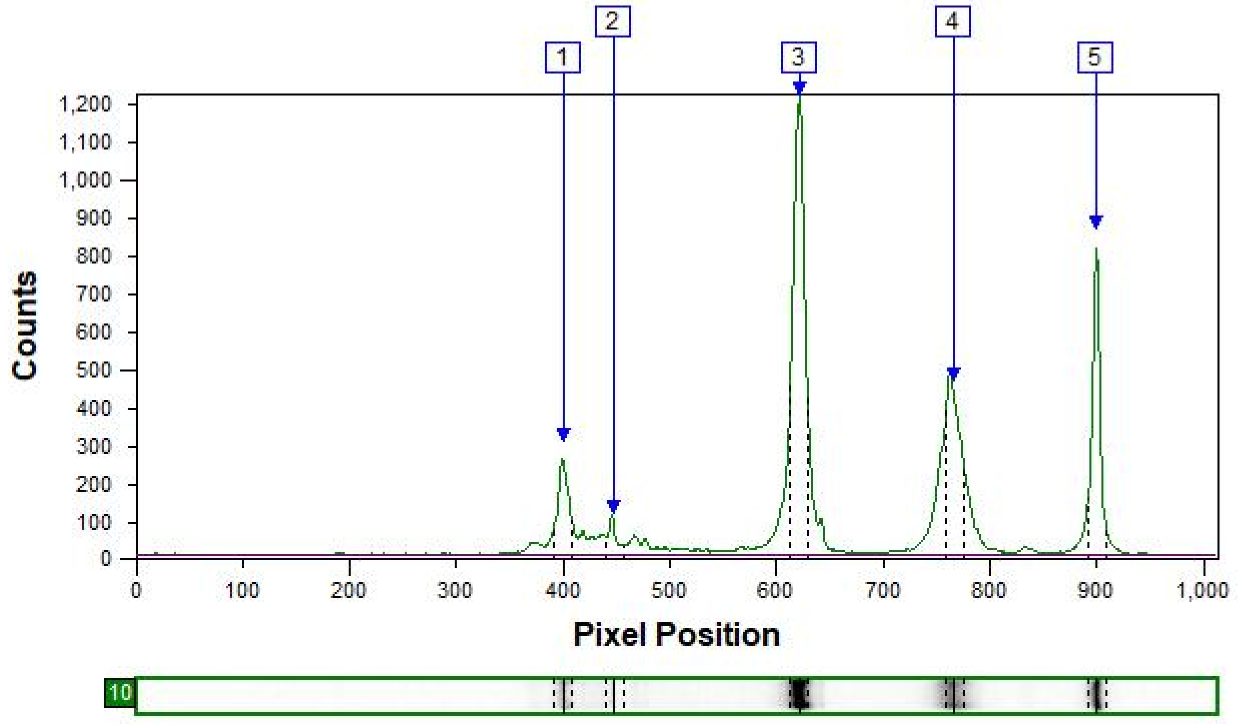

Band Table

Band No	Volume	Vol+BkGnd	Band %
1	96572.28	103433.66	9.236
2	29895.84	36757.22	2.859
3	468758.00	475619.38	44.830
4	215896.88	222758.27	20.648
5	234503.06	241364.44	22.427

ImageQuant TL

Lane Data Report: rw 2025_10_22 p6b timecourse t18h-[phosphor].gel, Lane 11

Summary

**Name:**	Lane 11

Background

**Background Type:**	Image Rectangle
**Rectangle:**	Left 278, Right 928, Top 972, Bottom 1100

Band Detection

**Automatic Detection:**	Yes
**Minimum Band Slope:**	184
**Median Filter:**	5
**Percentage Maximum Peak:**	0
**Maximum Peak Measure:**	Gel
**Edge Detection Method:**	Fixed Width
**Fixed Width pixels:**	17
**Band Positions Edited:**	Yes

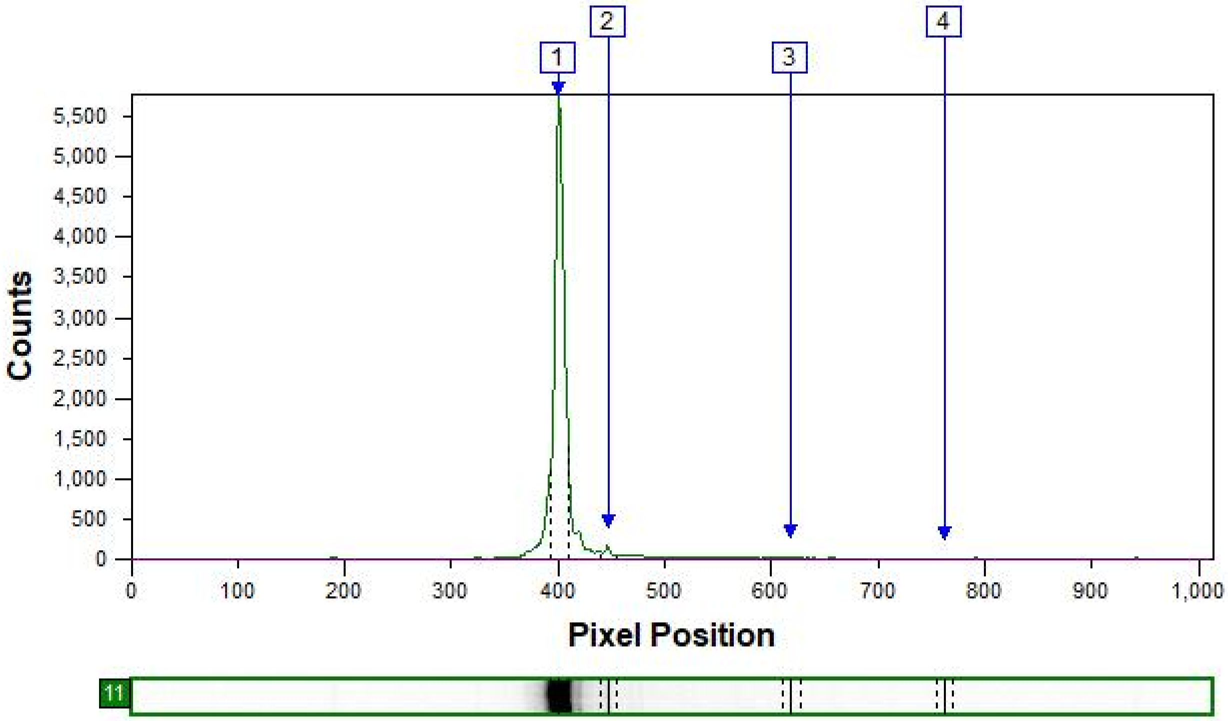

Band Table

Band No	Volume	Vol+BkGnd	Band %
1	1946875.16	1953736.54	96.885
2	50424.65	57286.03	2.509
3	7706.56	14567.94	0.384
4	4467.35	11328.73	0.222

ImageQuant TL

Lane Data Report: rw 2025_10_22 p6b timecourse t18h-[phosphor].gel, Lane 12

Summary

**Name:**	Lane 12

Background

**Background Type:**	Image Rectangle
**Rectangle:**	Left 278, Right 928, Top 972, Bottom 1100

Band Detection

**Automatic Detection:**	Yes
**Minimum Band Slope:**	184
**Median Filter:**	5
**Percentage Maximum Peak:**	0
**Maximum Peak Measure:**	Gel
**Edge Detection Method:**	Fixed Width
**Fixed Width pixels:**	17
**Band Positions Edited:**	Yes

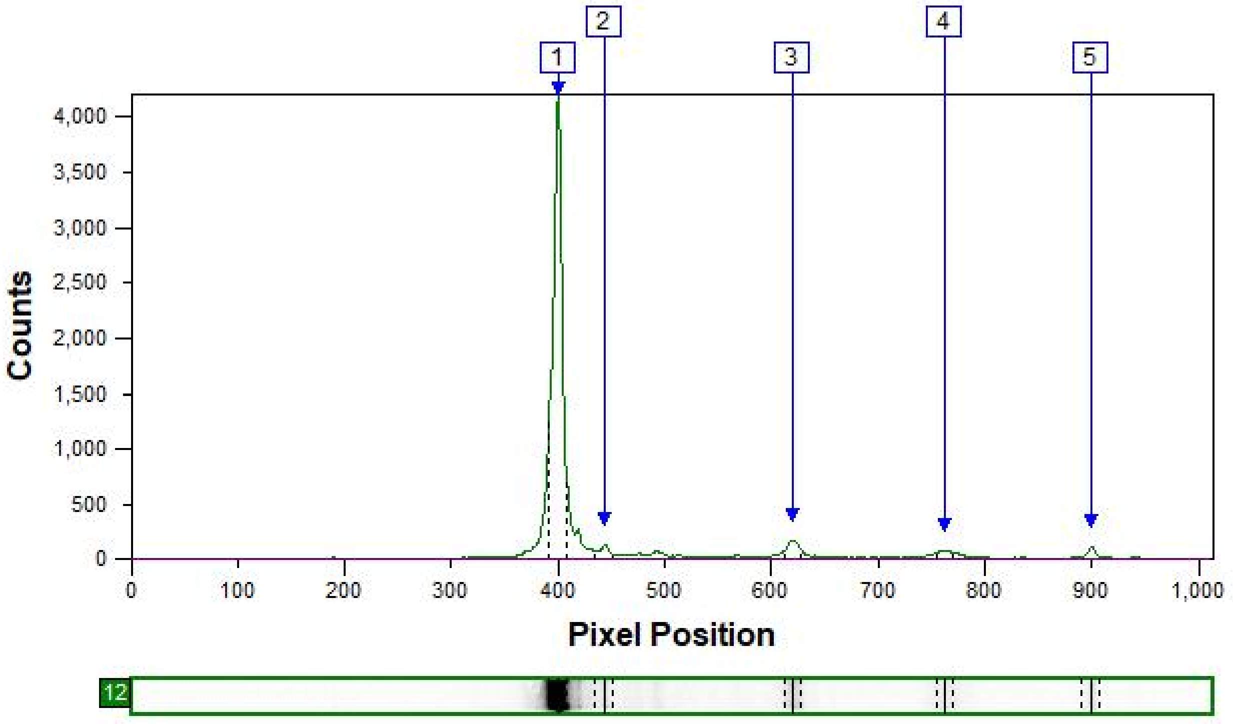

Band Table

Band No	Volume	Vol+BkGnd	Band %
1	1402956.41	1409817.80	88.565
2	44847.85	51709.23	2.831
3	67827.63	74689.01	4.282
4	35568.66	42430.04	2.245
5	32892.17	39753.55	2.076

ImageQuant TL

Lane Data Report: rw 2025_10_22 p6b timecourse t18h-[phosphor].gel, Lane 13

Summary

**Name:**	Lane 13

Background

**Background Type:**	Image Rectangle
**Rectangle:**	Left 278, Right 928, Top 972, Bottom 1100

Band Detection

**Automatic Detection:**	Yes
**Minimum Band Slope:**	184
**Median Filter:**	5
**Percentage Maximum Peak:**	0
**Maximum Peak Measure:**	Gel
**Edge Detection Method:**	Fixed Width
**Fixed Width pixels:**	17
**Band Positions Edited:**	Yes

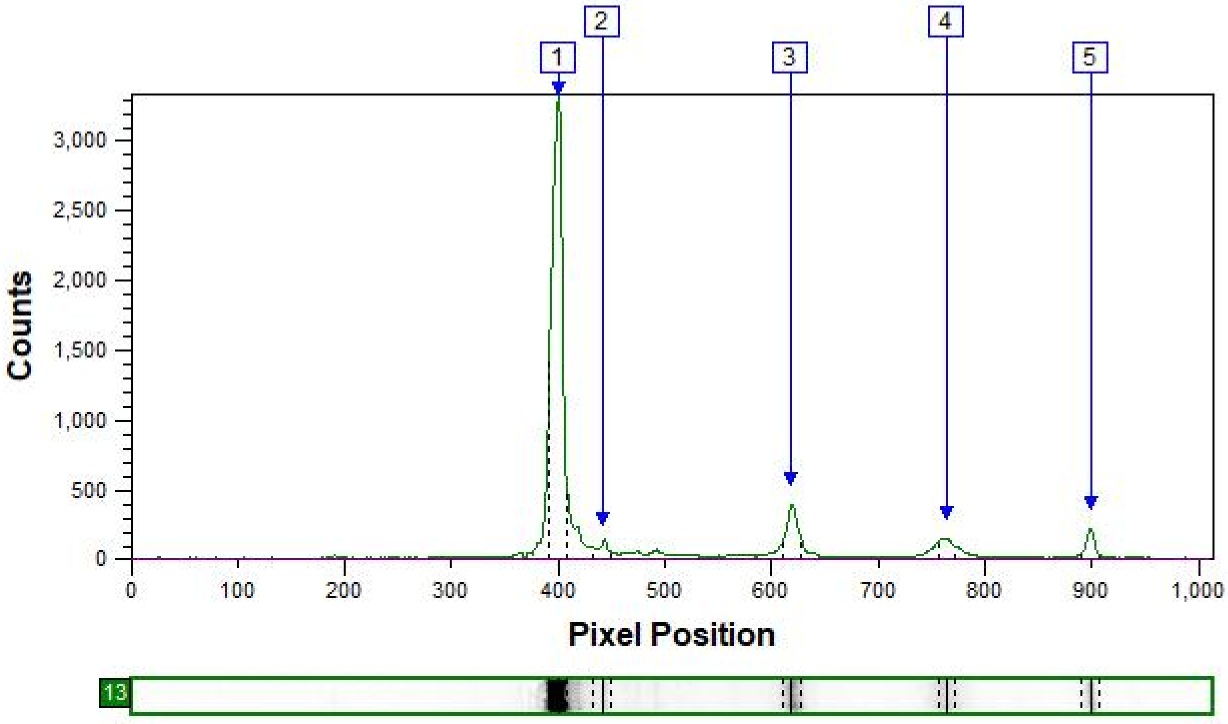

Band Table

Band No	Volume	Vol+BkGnd	Band %
1	1273829.22	1280690.60	79.645
2	46525.58	53386.96	2.909
3	142076.27	148937.65	8.883
4	69894.92	76756.30	4.370
5	67057.74	73919.12	4.193

IQTL Report version 8.1
Time of Report: 3:31 PM 10/22/2025 (Eastern Daylight Time)

ImageQuant TL

Lane Data Report: rw 2025_10_22 p6b timecourse t18h-[phosphor].gel, Lane 14

Summary

**Name:**	Lane 14

Background

**Background Type:**	Image Rectangle
**Rectangle:**	Left 278, Right 928, Top 972, Bottom 1100

Band Detection

**Automatic Detection:**	Yes
**Minimum Band Slope:**	184
**Median Filter:**	5
**Percentage Maximum Peak:**	0
**Maximum Peak Measure:**	Gel
**Edge Detection Method:**	Fixed Width
**Fixed Width pixels:**	17
**Band Positions Edited:**	Yes

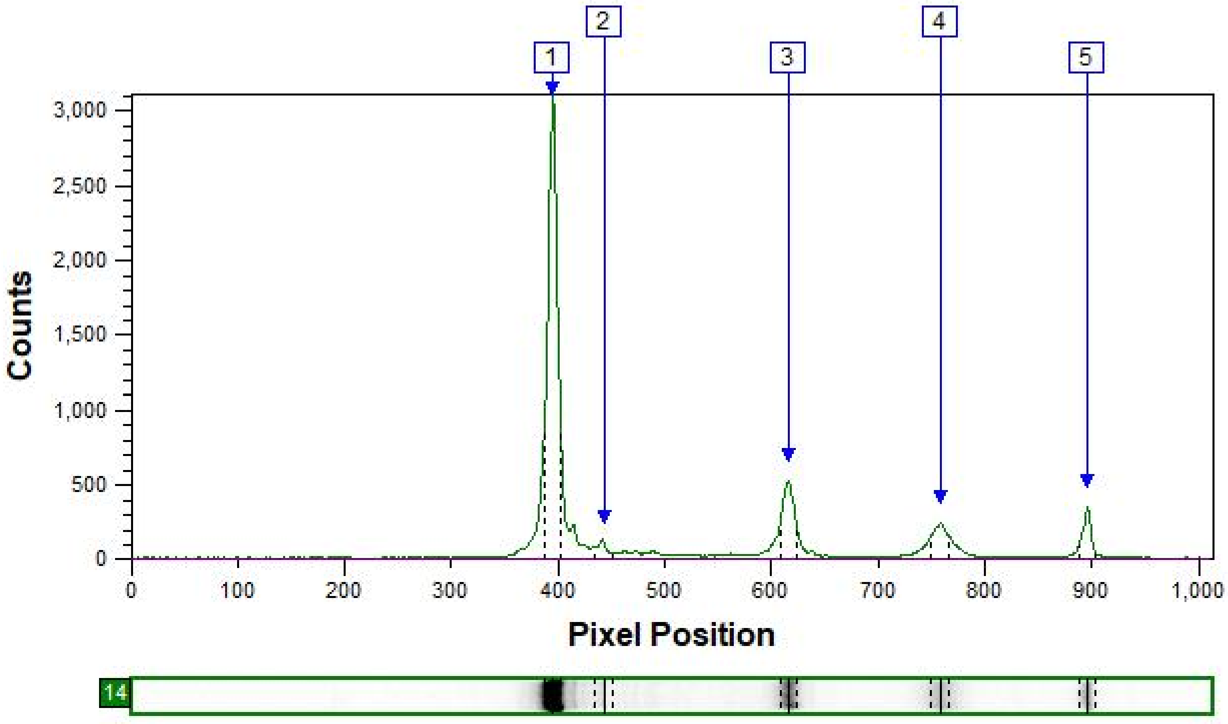

Band Table

Band No	Volume	Vol+BkGnd	Band %
1	1044950.07	1051811.45	69.403
2	40850.75	47712.13	2.713
3	214118.73	220980.11	14.221
4	104965.08	111826.46	6.971
5	100748.73	107610.11	6.691

Lane Data Report: rw 2025_10_22 p6b timecourse t18h-[phosphor].gel, Lane 15

Summary

**Name:**	Lane 15

Background

**Background Type:**	Image Rectangle
**Rectangle:**	Left 278, Right 928, Top 972, Bottom 1100

Band Detection

**Automatic Detection:**	Yes
**Minimum Band Slope:**	184
**Median Filter:**	5
**Percentage Maximum Peak:**	0
**Maximum Peak Measure:**	Gel
**Edge Detection Method:**	Fixed Width
**Fixed Width pixels:**	17
**Band Positions Edited:**	Yes

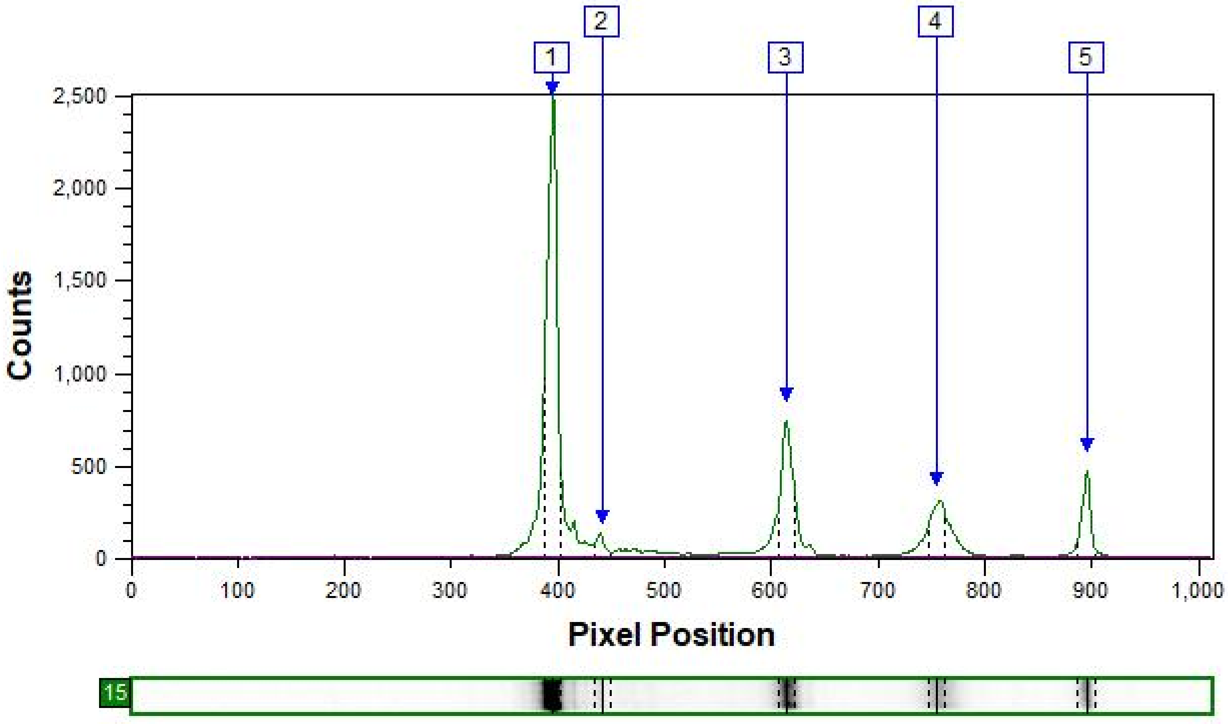

Band Table

Band No	Volume	Vol+BkGnd	Band %
1	886878.49	893739.87	59.109
2	41869.33	48730.71	2.791
3	290790.58	297651.96	19.381
4	140553.22	147414.60	9.368
5	140314.96	147176.34	9.352

Lane Data Report: rw 2025_10_22 p6b timecourse t18h-[phosphor].gel, Lane 16

Summary

**Name:**	Lane 16

Background

**Background Type:**	Image Rectangle
**Rectangle:**	Left 278, Right 928, Top 972, Bottom 1100

Band Detection

**Automatic Detection:**	Yes
**Minimum Band Slope:**	184
**Median Filter:**	5
**Percentage Maximum Peak:**	0
**Maximum Peak Measure:**	Gel
**Edge Detection Method:**	Fixed Width
**Fixed Width pixels:**	17
**Band Positions Edited:**	Yes

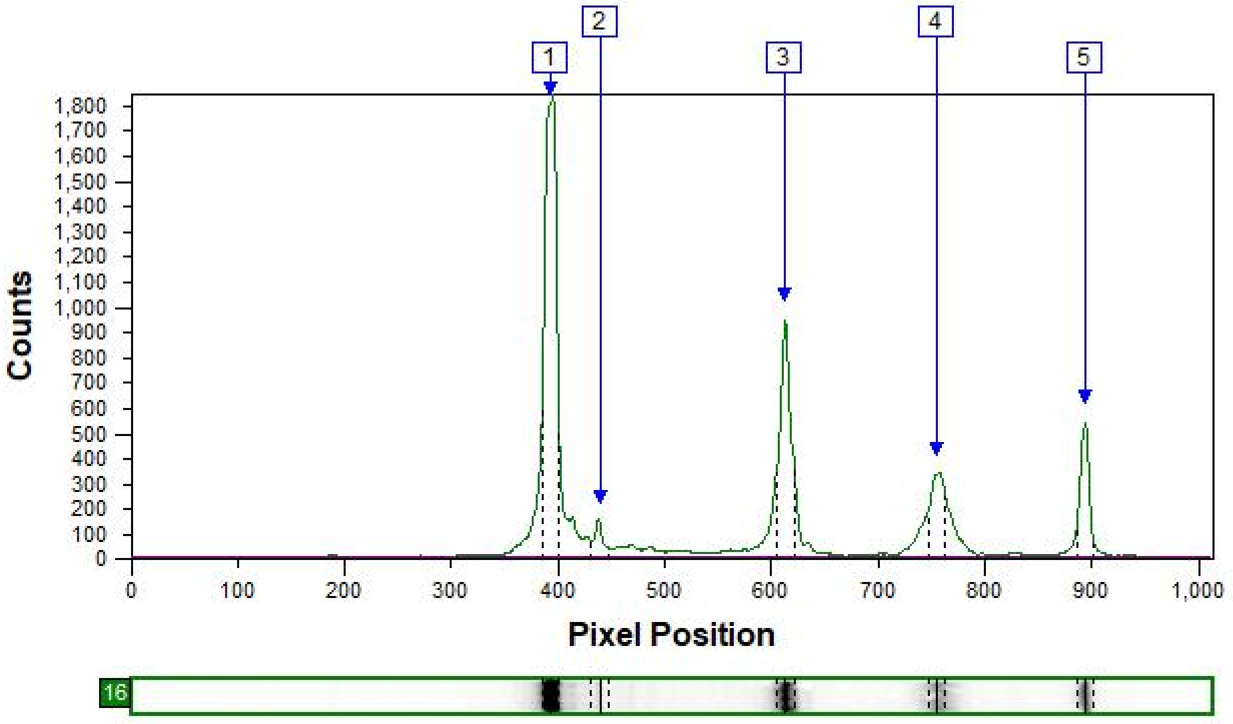

Band Table

Band No	Volume	Vol+BkGnd	Band %
1	767260.27	774121.65	51.717
2	43259.41	50120.79	2.916
3	348707.69	355569.07	23.504
4	155355.89	162217.27	10.472
5	169001.12	175862.50	11.391

ImageQuant TL

Lane Data Report: rw 2025_10_22 p6b timecourse t18h-[phosphor].gel, Lane 17

Summary

**Name:**	Lane 17

Background

**Background Type:**	Image Rectangle
**Rectangle:**	Left 278, Right 928, Top 972, Bottom 1100

Band Detection

**Automatic Detection:**	Yes
**Minimum Band Slope:**	184
**Median Filter:**	5
**Percentage Maximum Peak:**	0
**Maximum Peak Measure:**	Gel
**Edge Detection Method:**	Fixed Width
**Fixed Width pixels:**	17
**Band Positions Edited:**	Yes

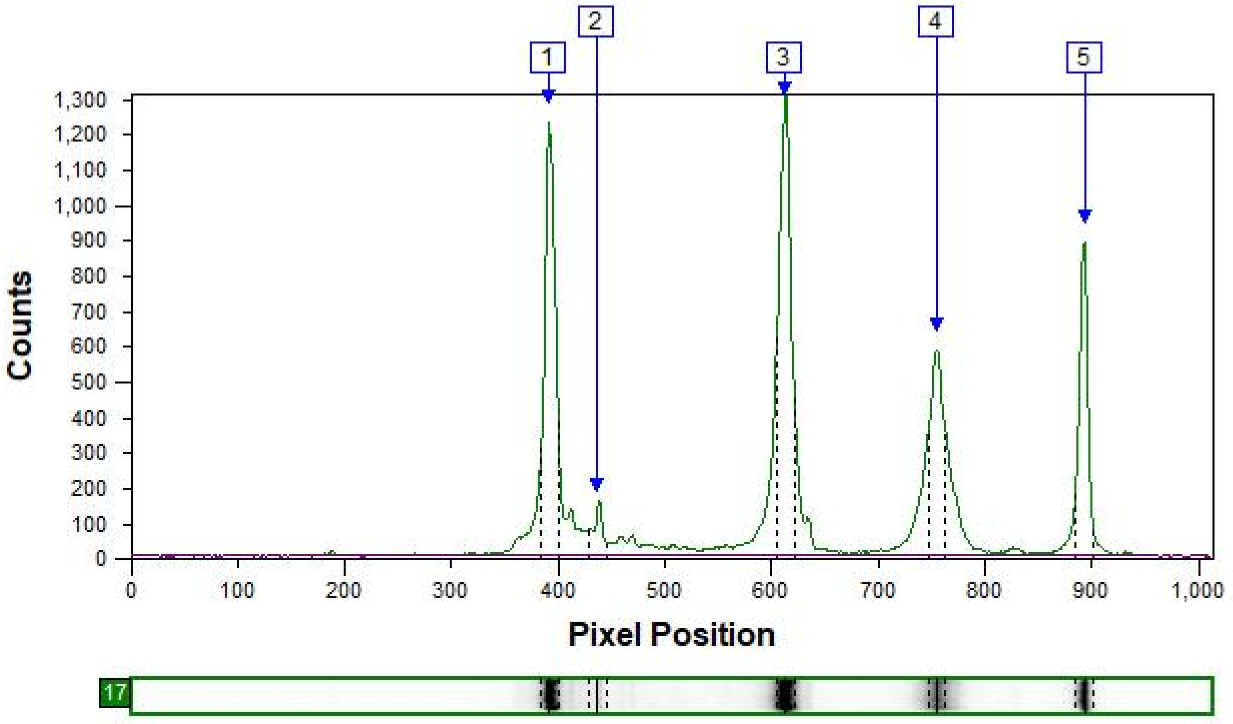

Band Table

Band No	Volume	Vol+BkGnd	Band %
1	433821.13	440682.51	28.303
2	46297.95	53159.33	3.021
3	536145.85	543007.23	34.979
4	254356.87	261218.25	16.594
5	262160.78	269022.16	17.104

ImageQuant TL

Lane Data Report: rw 2025_10_22 p6b timecourse t18h-[phosphor].gel, Lane 18

Summary

**Name:**	Lane 18

Background

**Background Type:**	Image Rectangle
**Rectangle:**	Left 278, Right 928, Top 972, Bottom 1100

Band Detection

**Automatic Detection:**	Yes
**Minimum Band Slope:**	184
**Median Filter:**	5
**Percentage Maximum Peak:**	0
**Maximum Peak Measure:**	Gel
**Edge Detection Method:**	Fixed Width
**Fixed Width pixels:**	17
**Band Positions Edited:**	Yes

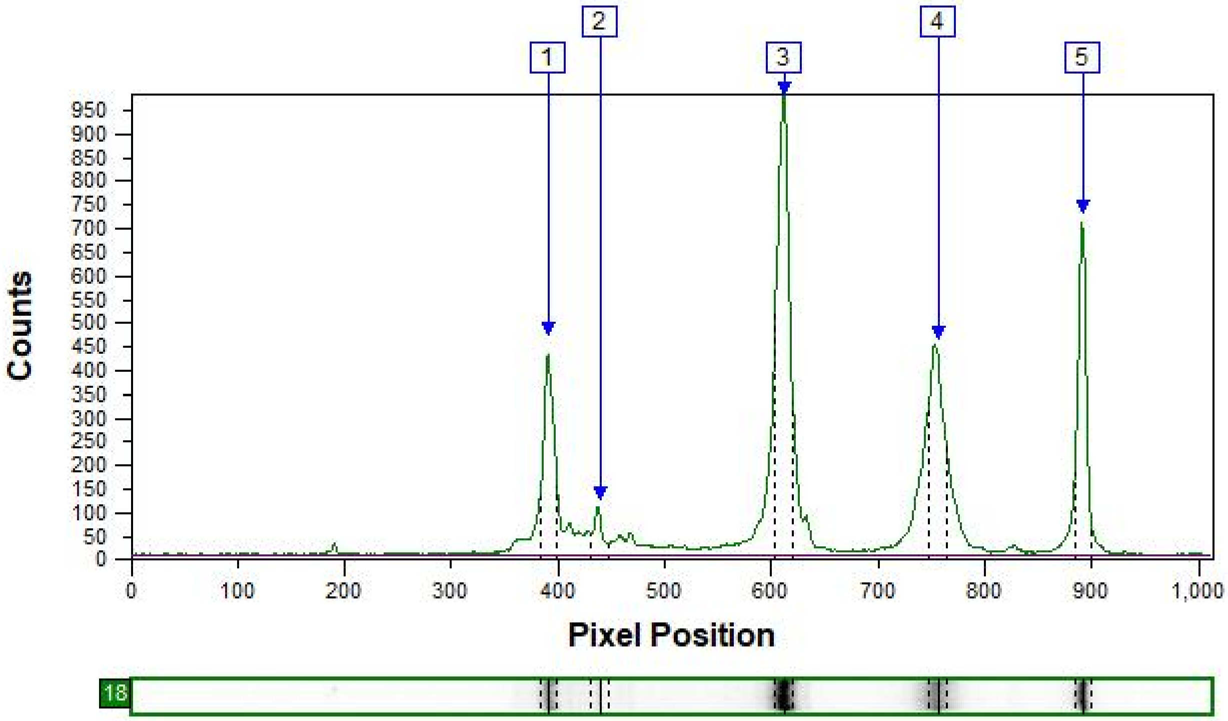

Band Table

Band No	Volume	Vol+BkGnd	Band %
1	153409.69	160271.07	15.503
2	28494.30	35355.68	2.880
3	406700.50	413561.88	41.100
4	196435.33	203296.71	19.851
5	204507.26	211368.64	20.667

ImageQuant TL

Lane Data Report: rw 2025_10_22 p6b timecourse t18h-[phosphor].gel, Lane 19

Summary

**Name:**	Lane 19

Background

**Background Type:**	Image Rectangle
**Rectangle:**	Left 278, Right 928, Top 972, Bottom 1100

Band Detection

**Automatic Detection:**	Yes
**Minimum Band Slope:**	184
**Median Filter:**	5
**Percentage Maximum Peak:**	0
**Maximum Peak Measure:**	Gel
**Edge Detection Method:**	Fixed Width
**Fixed Width pixels:**	17
**Band Positions Edited:**	Yes

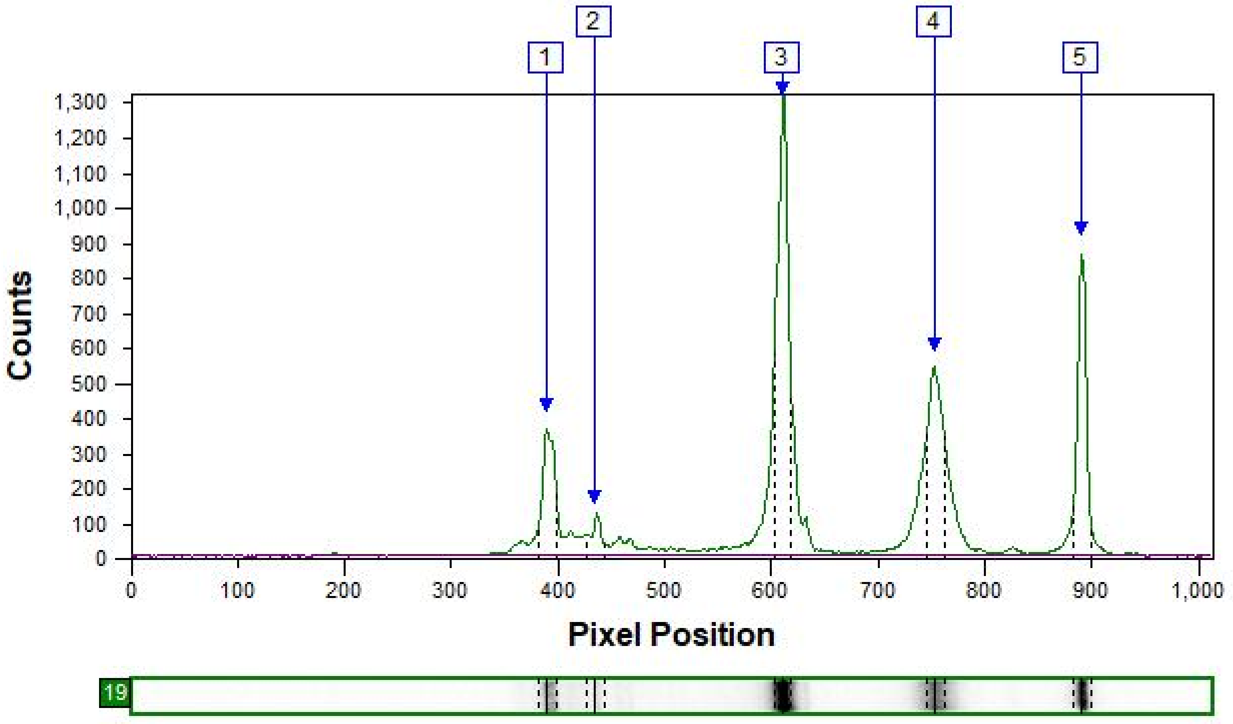

Band Table

Band No	Volume	Vol+BkGnd	Band %
1	144625.17	151486.55	11.921
2	38306.32	45167.70	3.157
3	520722.02	527583.41	42.920
4	247232.68	254094.06	20.378
5	262355.42	269216.81	21.624

Lane Data Report: rw 2025_10_22 p6b timecourse t18h-[phosphor].gel, Lane 20

Summary

**Name:**	Lane 20

Background

**Background Type:**	Image Rectangle
**Rectangle:**	Left 278, Right 928, Top 972, Bottom 1100

Band Detection

**Automatic Detection:**	Yes
**Minimum Band Slope:**	184
**Median Filter:**	5
**Percentage Maximum Peak:**	0
**Maximum Peak Measure:**	Gel
**Edge Detection Method:**	Fixed Width
**Fixed Width pixels:**	17
**Band Positions Edited:**	Yes

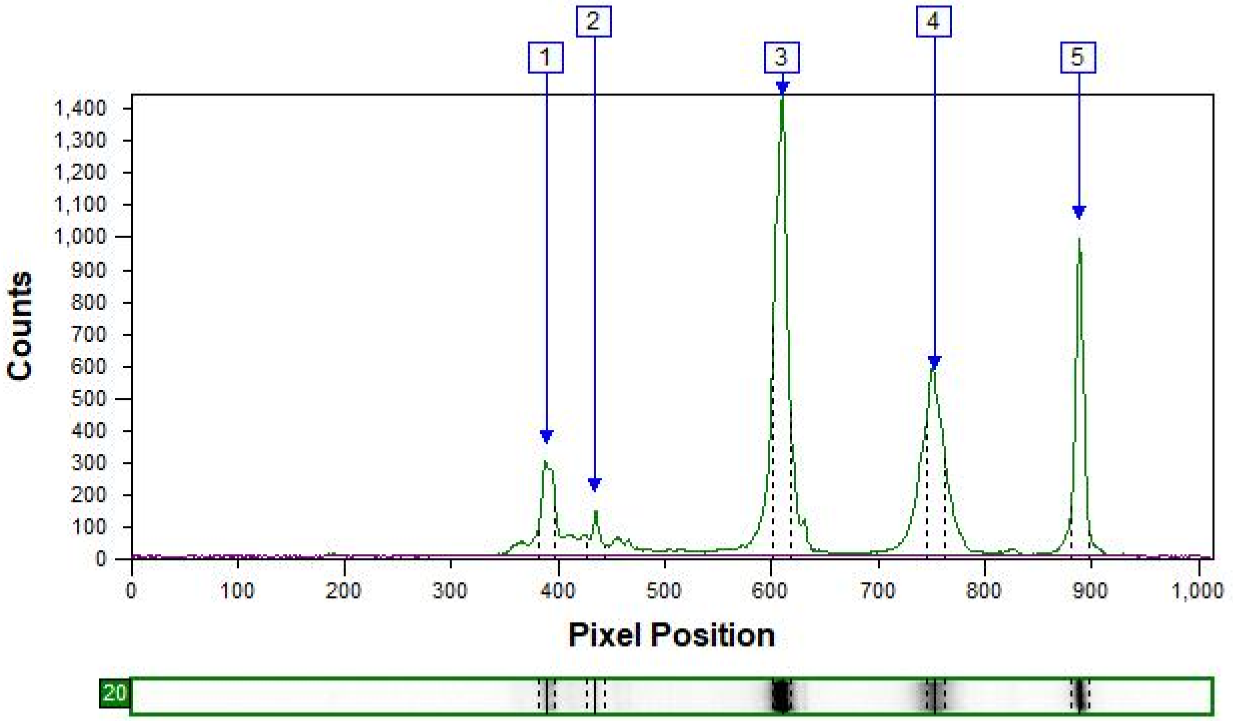

Band Table

Band No	Volume	Vol+BkGnd	Band %
1	122193.44	129054.82	9.511
2	40510.05	47371.43	3.153
3	570504.96	577366.34	44.407
4	265259.34	272120.72	20.647
5	286238.89	293100.27	22.280

ImageQuant TL

Lane Data Report: rw 2025_10_22 p6b timecourse t18h-[phosphor].gel, Lane 21

Summary

**Name:**	Lane 21

Background

**Background Type:**	Image Rectangle
**Rectangle:**	Left 278, Right 928, Top 972, Bottom 1100

Band Detection

**Automatic Detection:**	Yes
**Minimum Band Slope:**	184
**Median Filter:**	5
**Percentage Maximum Peak:**	0
**Maximum Peak Measure:**	Gel
**Edge Detection Method:**	Fixed Width
**Fixed Width pixels:**	17
**Band Positions Edited:**	Yes

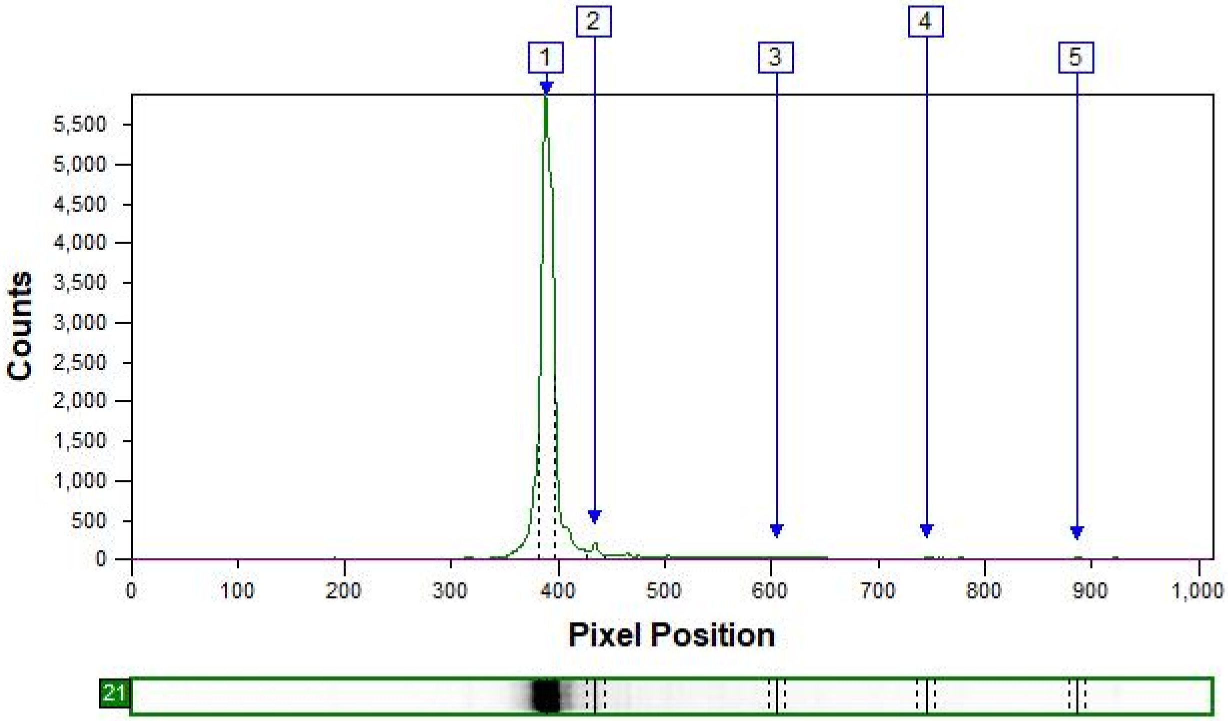

Band Table

Band No	Volume	Vol+BkGnd	Band %
1	2312285.27	2319146.65	96.267
2	63910.93	70772.31	2.661
3	12913.87	19775.25	0.538
4	6789.12	13650.50	0.283
5	6048.02	12909.40	0.252

ImageQuant TL

Lane Data Report: rw 2025_10_22 p6b timecourse t18h-[phosphor].gel, Lane 22

Summary

**Name:**	Lane 22

Background

**Background Type:**	Image Rectangle
**Rectangle:**	Left 278, Right 928, Top 972, Bottom 1100

Band Detection

**Automatic Detection:**	Yes
**Minimum Band Slope:**	184
**Median Filter:**	5
**Percentage Maximum Peak:**	0
**Maximum Peak Measure:**	Gel
**Edge Detection Method:**	Fixed Width
**Fixed Width pixels:**	17
**Band Positions Edited:**	Yes

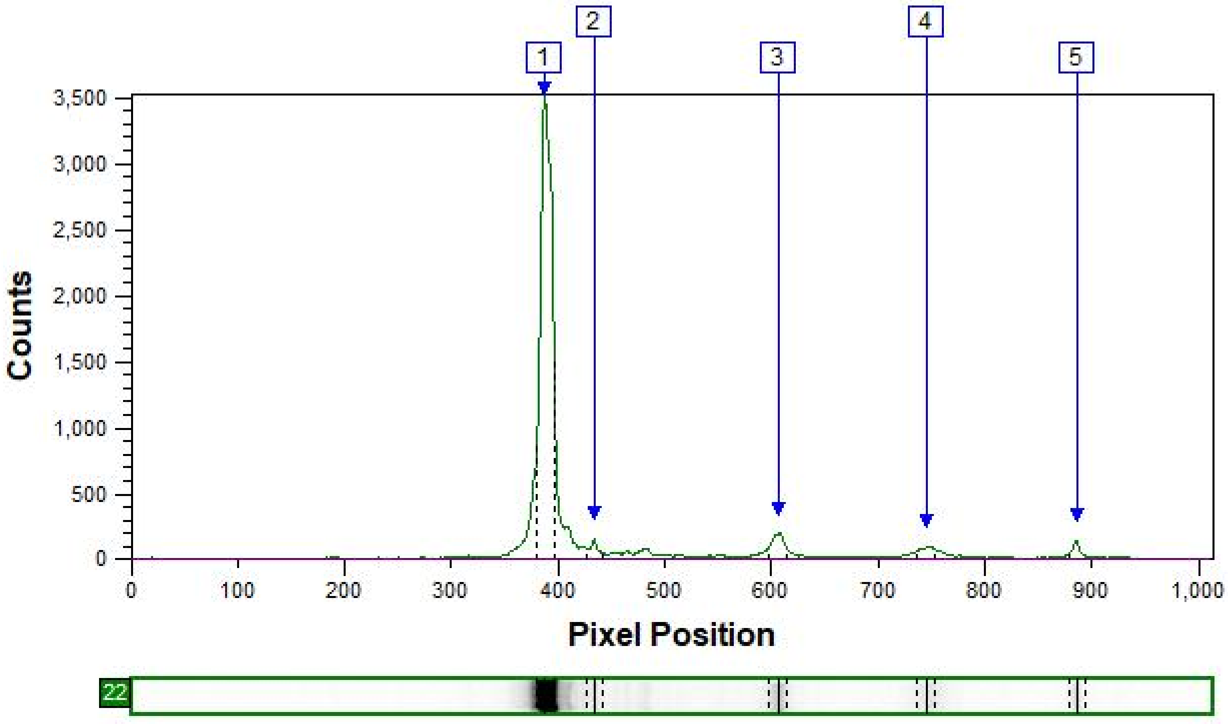

Band Table

Band No	Volume	Vol+BkGnd	Band %
1	1405277.29	1412138.67	87.394
2	44760.74	51622.13	2.784
3	79286.95	86148.33	4.931
4	40557.53	47418.91	2.522
5	38100.42	44961.80	2.369

IQTL Report version 8.1
Time of Report: 3:31 PM 10/22/2025 (Eastern Daylight Time)

ImageQuant TL

Lane Data Report: rw 2025_10_22 p6b timecourse t18h-[phosphor].gel, Lane 23

Summary

**Name:**	Lane 23

Background

**Background Type:**	Image Rectangle
**Rectangle:**	Left 278, Right 928, Top 972, Bottom 1100

Band Detection

**Automatic Detection:**	Yes
**Minimum Band Slope:**	184
**Median Filter:**	5
**Percentage Maximum Peak:**	0
**Maximum Peak Measure:**	Gel
**Edge Detection Method:**	Fixed Width
**Fixed Width pixels:**	17
**Band Positions Edited:**	Yes

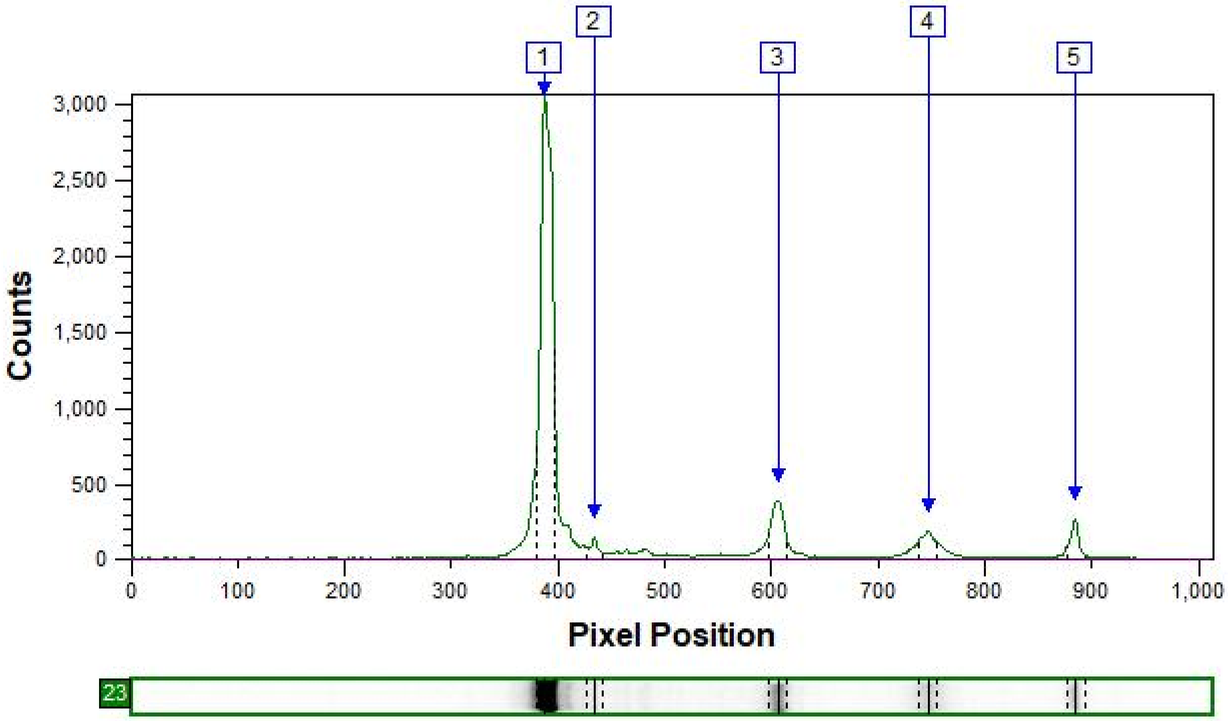

Band Table

Band No	Volume	Vol+BkGnd	Band %
1	1232103.27	1238964.65	77.138
2	44388.73	51250.12	2.779
3	162104.39	168965.77	10.149
4	80409.11	87270.49	5.034
5	78260.74	85122.13	4.900

ImageQuant TL

Lane Data Report: rw 2025_10_22 p6b timecourse t18h-[phosphor].gel, Lane 24

Summary

**Name:**	Lane 24

Background

**Background Type:**	Image Rectangle
**Rectangle:**	Left 278, Right 928, Top 972, Bottom 1100

Band Detection

**Automatic Detection:**	Yes
**Minimum Band Slope:**	184
**Median Filter:**	5
**Percentage Maximum Peak:**	0
**Maximum Peak Measure:**	Gel
**Edge Detection Method:**	Fixed Width
**Fixed Width pixels:**	17
**Band Positions Edited:**	Yes

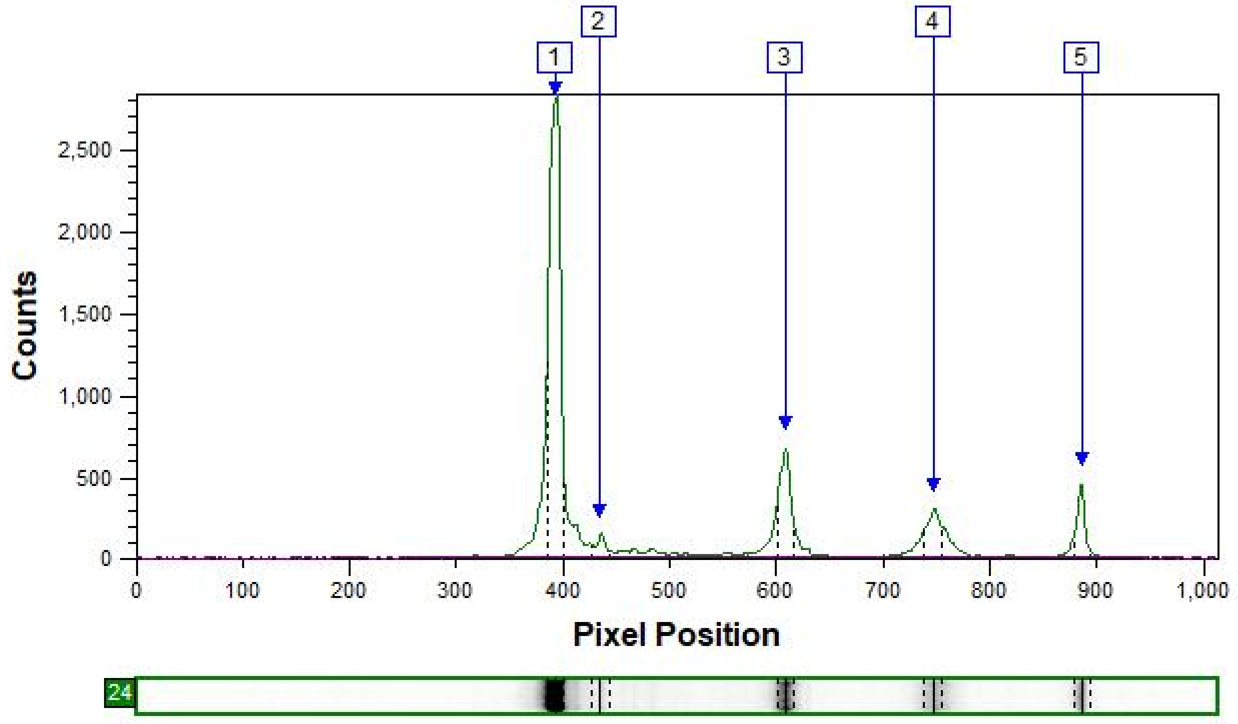

Band Table

Band No	Volume	Vol+BkGnd	Band %
1	1143430.50	1150291.88	66.437
2	48578.40	55439.78	2.823
3	264436.80	271298.18	15.365
4	131408.90	138270.28	7.635
5	133211.08	140072.46	7.740

ImageQuant TL

Lane Data Report: rw 2025_10_22 p6b timecourse t18h-[phosphor].gel, Lane 25

Summary

**Name:**	Lane 25

Background

**Background Type:**	Image Rectangle
**Rectangle:**	Left 278, Right 928, Top 972, Bottom 1100

Band Detection

**Automatic Detection:**	Yes
**Minimum Band Slope:**	184
**Median Filter:**	5
**Percentage Maximum Peak:**	0
**Maximum Peak Measure:**	Gel
**Edge Detection Method:**	Fixed Width
**Fixed Width pixels:**	17
**Band Positions Edited:**	Yes

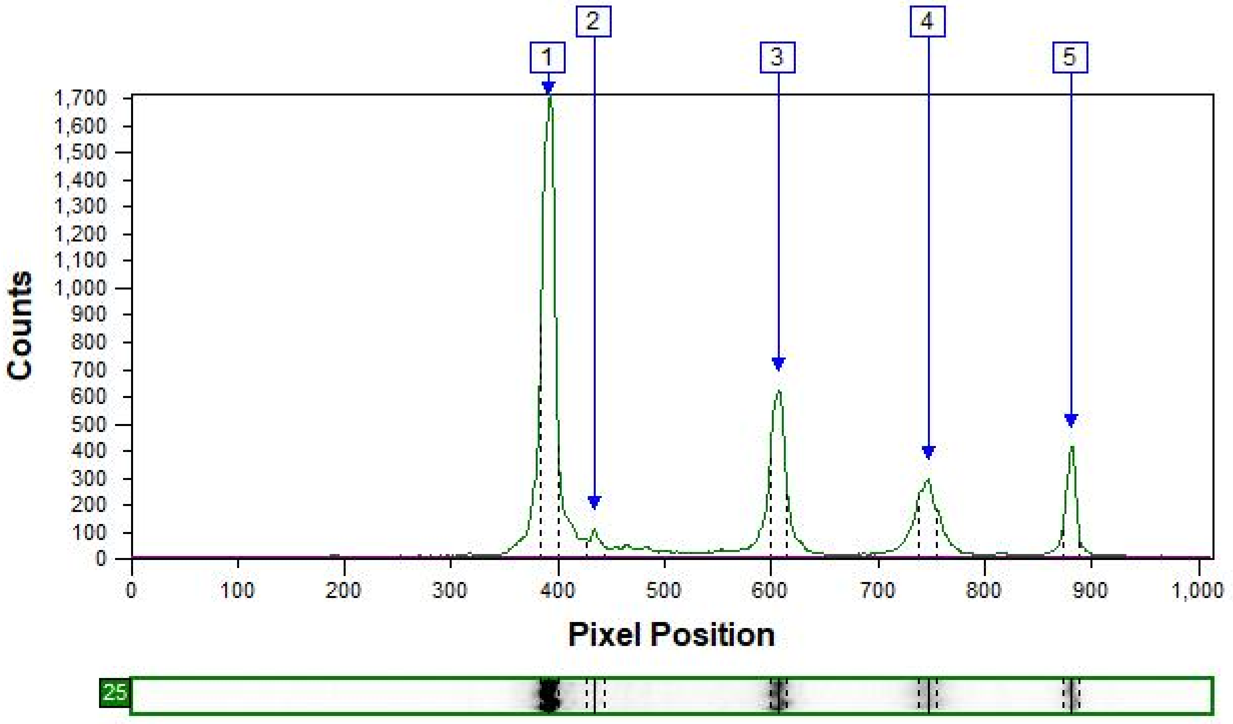

Band Table

Band No	Volume	Vol+BkGnd	Band %
1	713226.05	720087.43	55.089
2	37532.13	44393.51	2.899
3	270179.53	277040.91	20.868
4	132182.16	139043.54	10.210
5	141560.38	148421.76	10.934

ImageQuant TL

Lane Data Report: rw 2025_10_22 p6b timecourse t18h-[phosphor].gel, Lane 26

Summary

**Name:**	Lane 26

Background

**Background Type:**	Image Rectangle
**Rectangle:**	Left 278, Right 928, Top 972, Bottom 1100

Band Detection

**Automatic Detection:**	Yes
**Minimum Band Slope:**	184
**Median Filter:**	5
**Percentage Maximum Peak:**	0
**Maximum Peak Measure:**	Gel
**Edge Detection Method:**	Fixed Width
**Fixed Width pixels:**	17
**Band Positions Edited:**	Yes

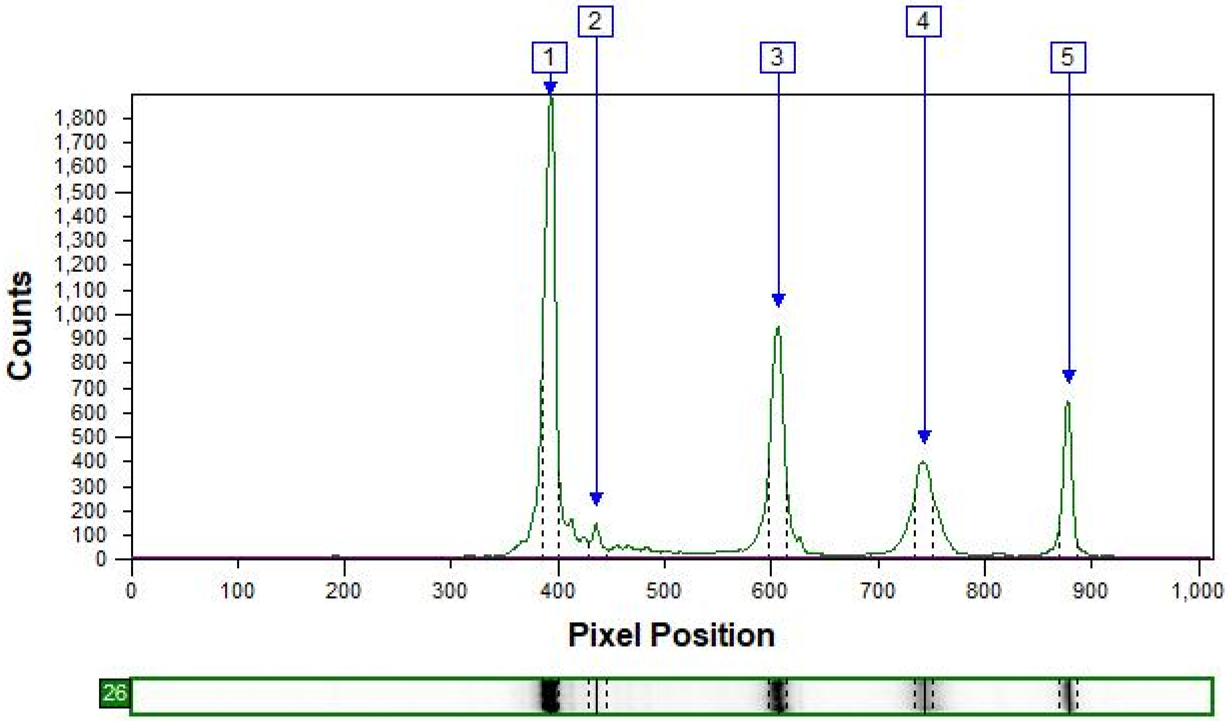

Band Table

Band No	Volume	Vol+BkGnd	Band %
1	704096.57	710957.95	46.434
2	42276.22	49137.60	2.788
3	385342.96	392204.34	25.413
4	189050.54	195911.92	12.468
5	195556.36	202417.74	12.897

ImageQuant TL

Lane Data Report: rw 2025_10_22 p6b timecourse t18h-[phosphor].gel, Lane 27

Summary

**Name:**	Lane 27

Background

**Background Type:**	Image Rectangle
**Rectangle:**	Left 278, Right 928, Top 972, Bottom 1100

Band Detection

**Automatic Detection:**	Yes
**Minimum Band Slope:**	184
**Median Filter:**	5
**Percentage Maximum Peak:**	0
**Maximum Peak Measure:**	Gel
**Edge Detection Method:**	Fixed Width
**Fixed Width pixels:**	17
**Band Positions Edited:**	Yes

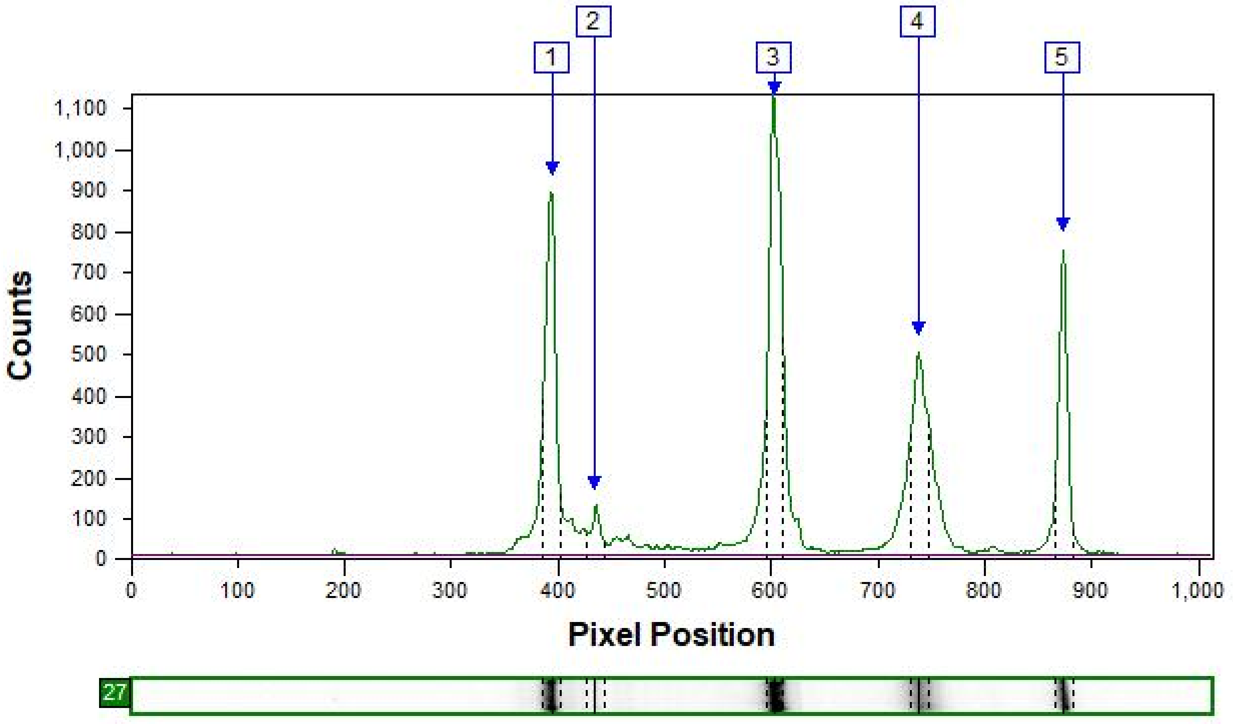

Band Table

Band No	Volume	Vol+BkGnd	Band %
1	315051.38	321912.76	25.073
2	38399.64	45261.02	3.056
3	452169.58	459030.96	35.986
4	218543.16	225404.55	17.393
5	232360.00	239221.38	18.492

ImageQuant TL

Lane Data Report: rw 2025_10_22 p6b timecourse t18h-[phosphor].gel, Lane 28

Summary

**Name:**	Lane 28

Background

**Background Type:**	Image Rectangle
**Rectangle:**	Left 278, Right 928, Top 972, Bottom 1100

Band Detection

**Automatic Detection:**	Yes
**Minimum Band Slope:**	184
**Median Filter:**	5
**Percentage Maximum Peak:**	0
**Maximum Peak Measure:**	Gel
**Edge Detection Method:**	Fixed Width
**Fixed Width pixels:**	17
**Band Positions Edited:**	Yes

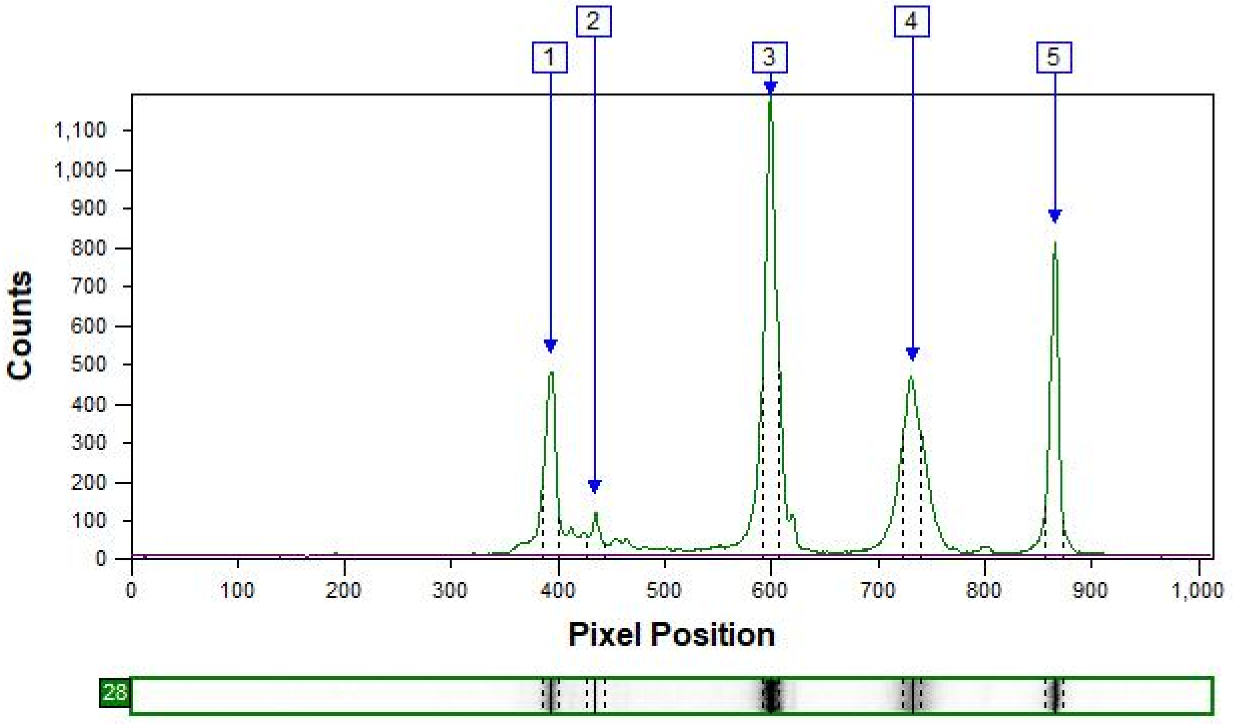

Band Table

Band No	Volume	Vol+BkGnd	Band %
1	172295.27	179157.36	15.482
2	34238.80	41103.00	3.077
3	451769.49	458621.47	40.594
4	213851.80	220702.59	19.216
5	240743.21	247619.77	21.632

ImageQuant TL

Lane Data Report: rw 2025_10_22 p6b timecourse t18h-[phosphor].gel, Lane 29

Summary

**Name:**	Lane 29

Background

**Background Type:**	Image Rectangle
**Rectangle:**	Left 278, Right 928, Top 972, Bottom 1100

Band Detection

**Automatic Detection:**	Yes
**Minimum Band Slope:**	184
**Median Filter:**	5
**Percentage Maximum Peak:**	0
**Maximum Peak Measure:**	Gel
**Edge Detection Method:**	Fixed Width
**Fixed Width pixels:**	17
**Band Positions Edited:**	Yes

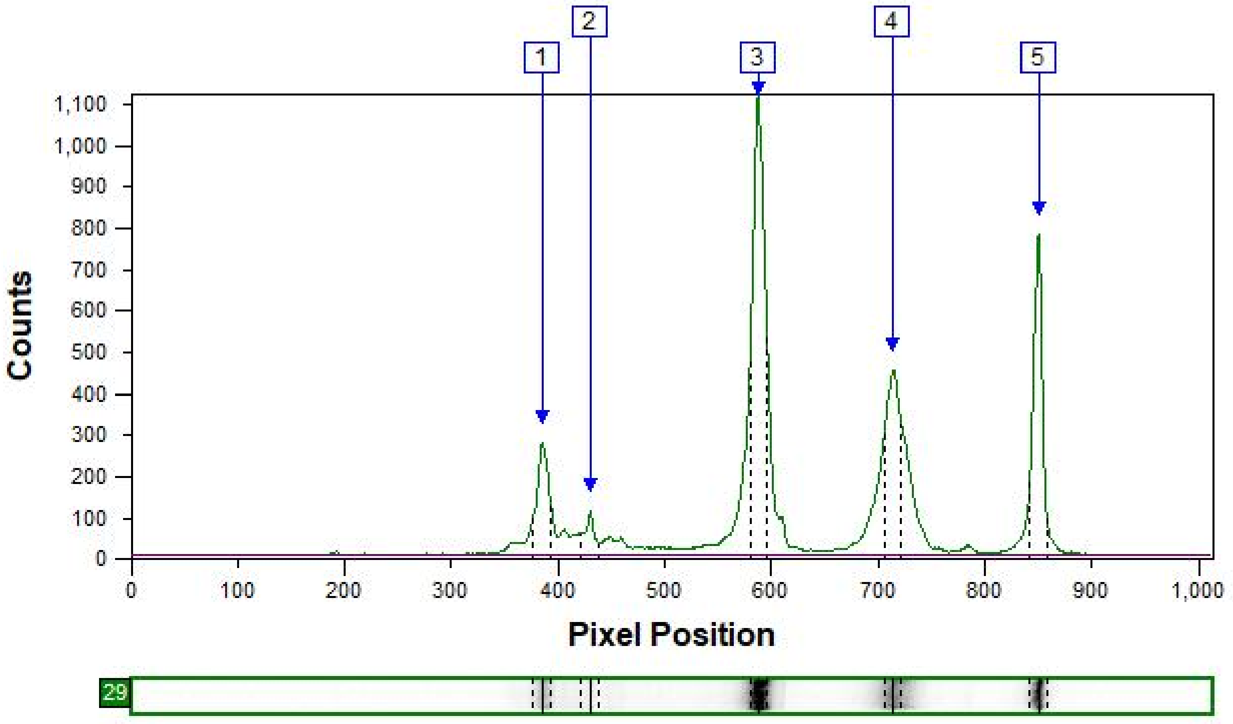

Band Table

Band No	Volume	Vol+BkGnd	Band %
1	107120.33	113974.04	10.271
2	33942.80	40770.52	3.255
3	450167.38	457010.40	43.164
4	208864.90	215729.42	20.027
5	242830.54	249703.28	23.284

ImageQuant TL

Lane Data Report: rw 2025_10_22 p6b timecourse t18h-[phosphor].gel, Lane 30

Summary

**Name:**	Lane 30

Background

**Background Type:**	Image Rectangle
**Rectangle:**	Left 278, Right 928, Top 972, Bottom 1100

Band Detection

**Automatic Detection:**	Yes
**Minimum Band Slope:**	184
**Median Filter:**	5
**Percentage Maximum Peak:**	0
**Maximum Peak Measure:**	Gel
**Edge Detection Method:**	Fixed Width
**Fixed Width pixels:**	17
**Band Positions Edited:**	Yes

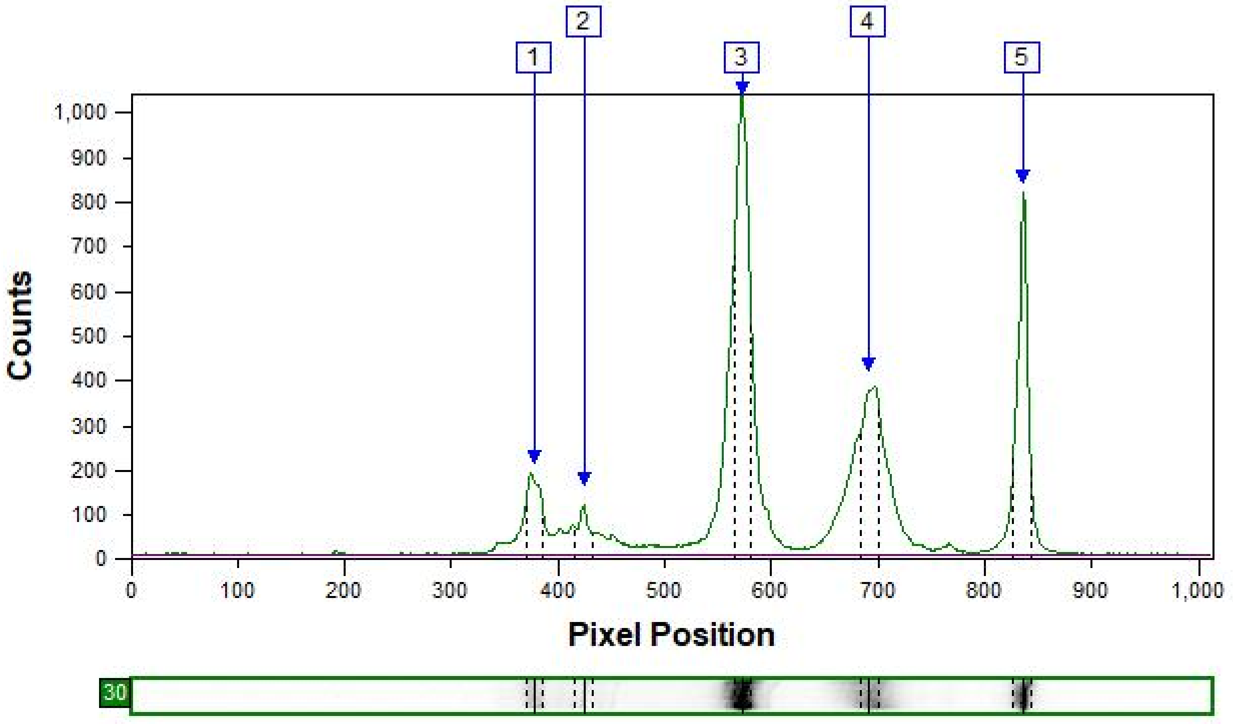

Band Table

Band No	Volume	Vol+BkGnd	Band %
1	83638.99	90487.10	8.059
2	38741.19	45552.84	3.733
3	457361.35	464167.21	44.068
4	188407.66	195283.00	18.153
5	269714.22	276638.64	25.987
