# Supplementary material for: PCanPIE: A group I intron platform for efficient circRNA synthesis at ambient temperatures
Source: RNA. Author manuscript; Available in PMC 2026 Nov 9. (PMC13580324; doi:10.1261/rna.081078.126)
Supplement: Supplementary file 1 [file NIHMS2209016-supplement-Data_source_files.zip › 14. Data source files/Fig. 3 Data/2025_10_22 P8 timecourse/rw 2025_10_22 p8 timecourse t18h-[phosphor]gel_AllLanes.pdf]

IQTL Report version 8.1
Time of Report: 3:42 PM 10/22/2025 (Eastern Daylight Time)

ImageQuant TL

Analysis Report: rw 2025_10_22 p8 timecourse t18h-[phosphor].gel

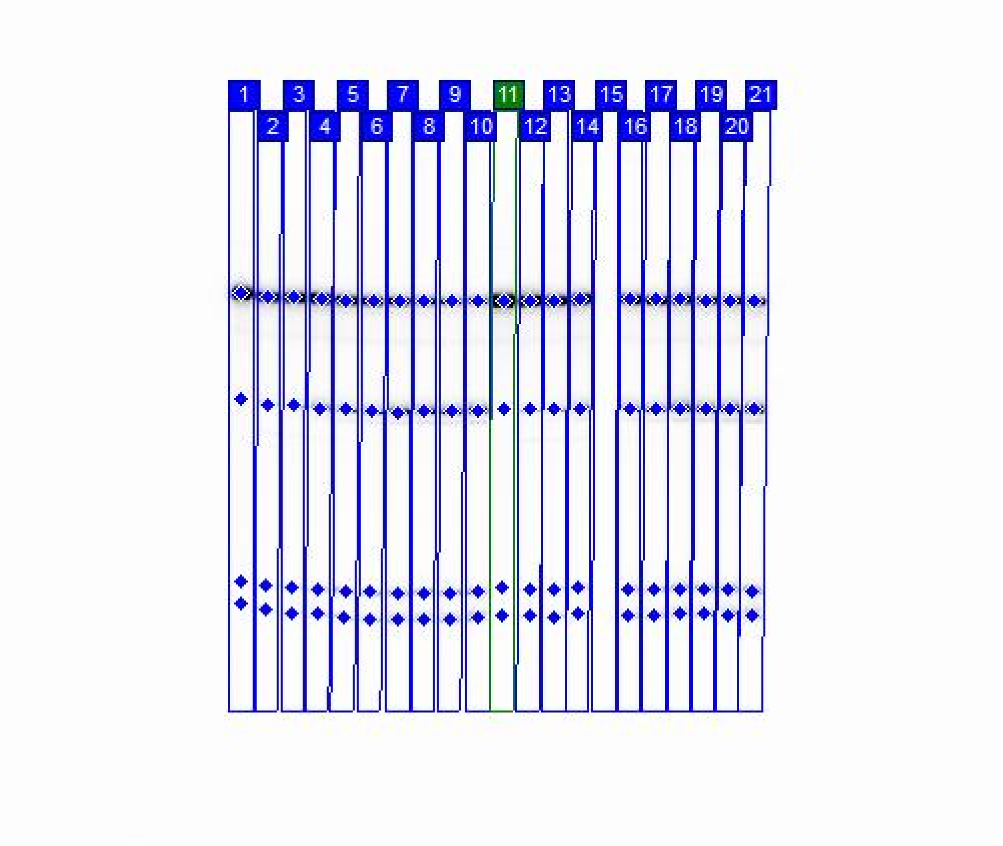

Lane Table

Lane	Bands	Band Volume	Lane Volume
Lane 1	4	1765862.53	2375618.83
Lane 2	4	1368250.00	1868918.19
Lane 3	4	1437364.71	1927051.47
Lane 4	4	1788541.37	2375731.56
Lane 5	4	1395426.86	1853996.95
Lane 6	4	1322653.30	1775181.15
Lane 7	4	1398795.90	1895023.69
Lane 8	4	1284769.72	1745813.46
Lane 9	4	1144634.27	1552415.27
Lane 10	4	1272587.33	1733824.97
Lane 11	4	2545992.77	3306266.31
Lane 12	4	1690666.49	2271110.94
Lane 13	4	1389039.92	1856485.99
Lane 14	4	1897867.01	2487022.83
Lane 15	0	0.00	93381.21
Lane 16	4	1475718.29	1954581.39
Lane 17	4	1338288.03	1812873.74
Lane 18	4	1791993.10	2449988.94
Lane 19	4	1469654.64	1992427.44
Lane 20	4	1477472.70	2014434.60
Lane 21	4	1505519.46	2053789.67

ImageQuant TL

Lane Data Report: rw 2025_10_22 p8 timecourse t18h-[phosphor].gel, Lane 1

Summary

**Name:**	Lane 1

Background

**Background Type:**	Image Rectangle
**Rectangle:**	Left 398, Right 1202, Top 1059, Bottom 1215

Band Detection

**Automatic Detection:**	No
**Edge Detection Method:**	Fixed Width
**Fixed Width pixels:**	17
**Band Positions Edited:**	Yes

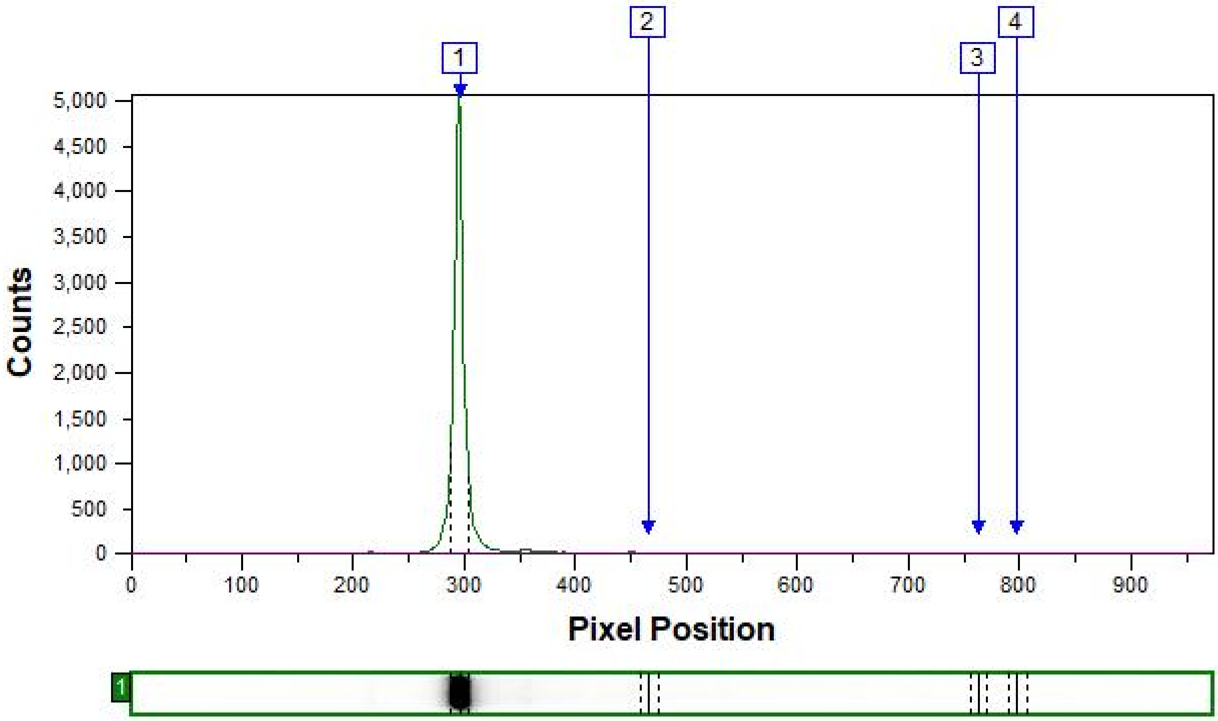

Band Table

Band No	Volume	Vol+BkGnd	Band %
1	1752247.94	1755553.07	99.229
2	6976.15	10284.80	0.395
3	2371.54	5650.57	0.134
4	4266.89	7550.21	0.242

ImageQuant TL

Lane Data Report: rw 2025_10_22 p8 timecourse t18h-[phosphor].gel, Lane 2

Summary

**Name:**	Lane 2

Background

**Background Type:**	Image Rectangle
**Rectangle:**	Left 398, Right 1202, Top 1059, Bottom 1215

Band Detection

**Automatic Detection:**	No
**Edge Detection Method:**	Fixed Width
**Fixed Width pixels:**	17
**Band Positions Edited:**	Yes

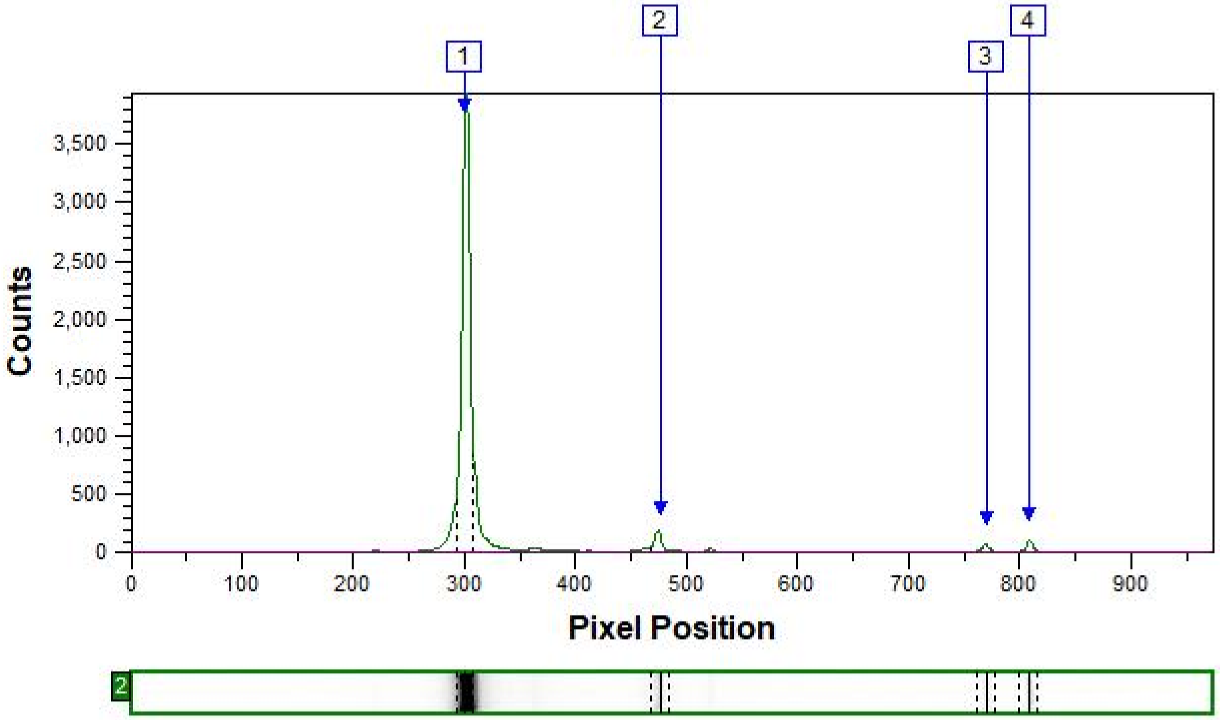

Band Table

Band No	Volume	Vol+BkGnd	Band %
1	1262984.52	1266287.00	92.307
2	55329.63	58633.95	4.044
3	21482.93	24786.90	1.570
4	28452.93	31762.53	2.080

ImageQuant TL

Lane Data Report: rw 2025_10_22 p8 timecourse t18h-[phosphor].gel, Lane 3

Summary

**Name:**	Lane 3

Background

**Background Type:**	Image Rectangle
**Rectangle:**	Left 398, Right 1202, Top 1059, Bottom 1215

Band Detection

**Automatic Detection:**	No
**Edge Detection Method:**	Fixed Width
**Fixed Width pixels:**	17
**Band Positions Edited:**	Yes

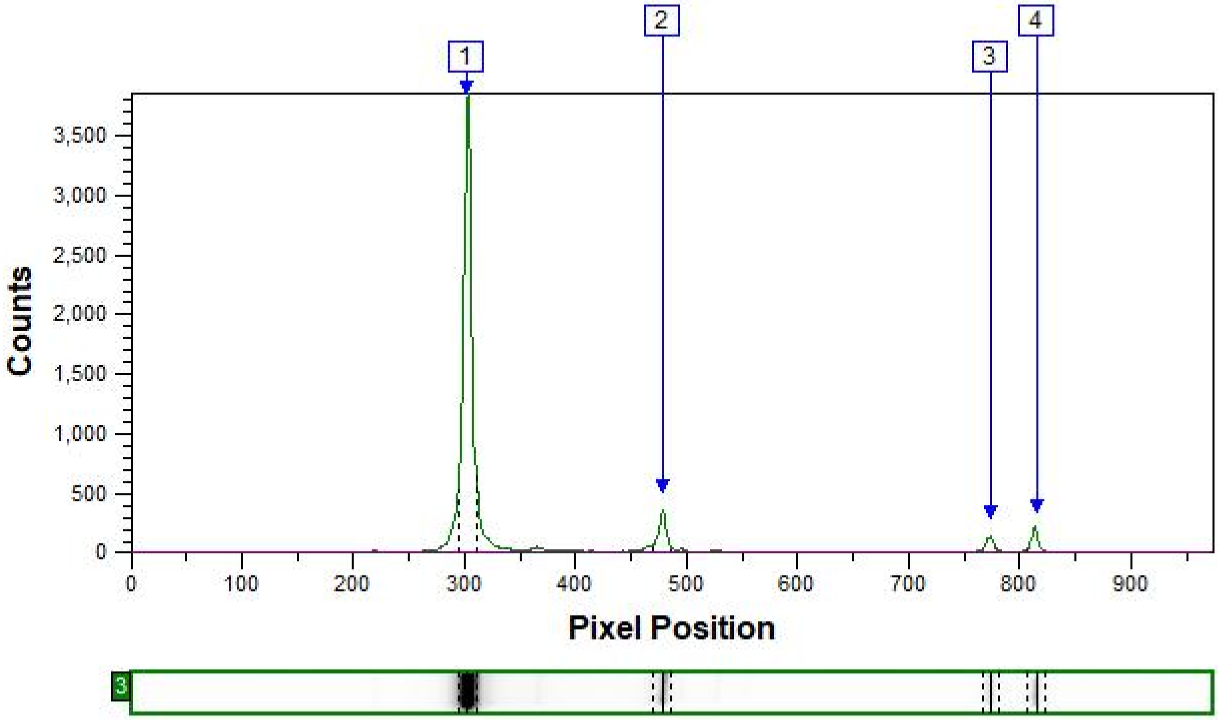

Band Table

Band No	Volume	Vol+BkGnd	Band %
1	1228467.42	1231770.01	85.467
2	107944.43	111247.02	7.510
3	43690.89	46993.48	3.040
4	57261.97	60564.56	3.984

Lane Data Report: rw 2025_10_22 p8 timecourse t18h-[phosphor].gel, Lane 4

Summary

**Name:**	Lane 4

Background

**Background Type:**	Image Rectangle
**Rectangle:**	Left 398, Right 1202, Top 1059, Bottom 1215

Band Detection

**Automatic Detection:**	Yes
**Minimum Band Slope:**	183
**Median Filter:**	5
**Percentage Maximum Peak:**	0
**Maximum Peak Measure:**	Gel
**Edge Detection Method:**	Fixed Width
**Fixed Width pixels:**	17
**Band Positions Edited:**	Yes

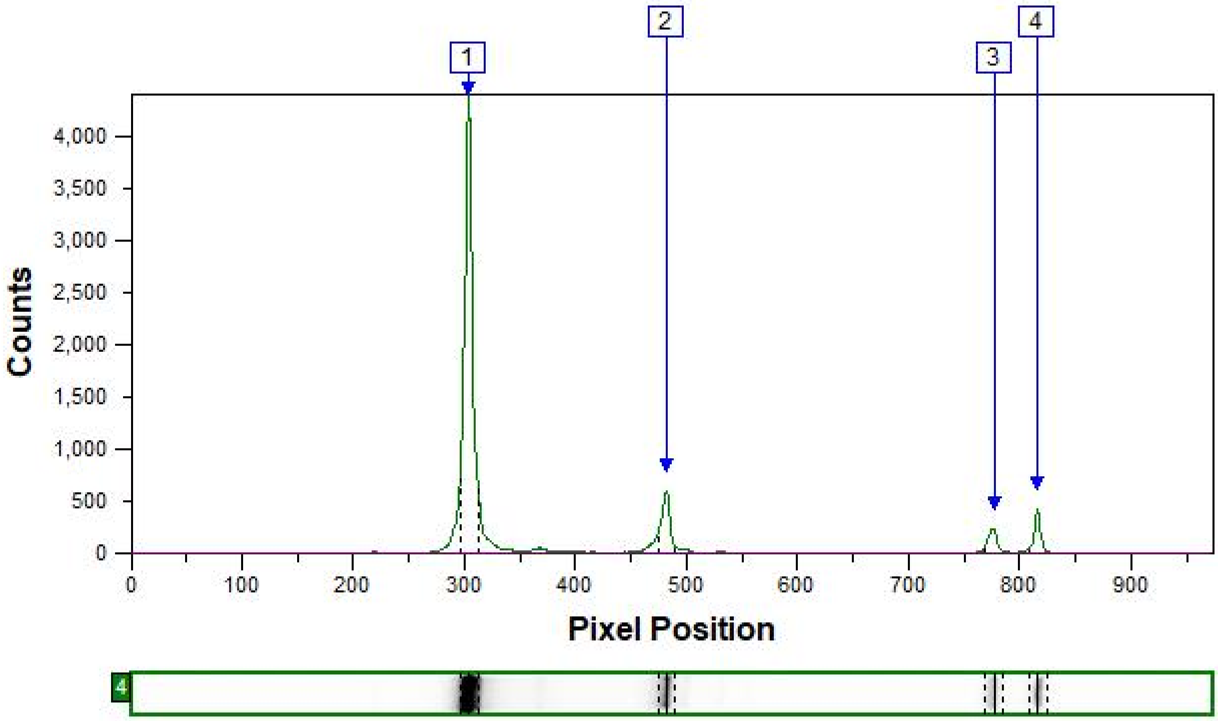

Band Table

Band No	Volume	Vol+BkGnd	Band %
1	1389911.25	1393213.85	77.712
2	201386.46	204689.06	11.260
3	87449.54	90752.14	4.889
4	109794.11	113096.70	6.139

ImageQuant TL

Lane Data Report: rw 2025_10_22 p8 timecourse t18h-[phosphor].gel, Lane 5

Summary

**Name:**	Lane 5

Background

**Background Type:**	Image Rectangle
**Rectangle:**	Left 398, Right 1202, Top 1059, Bottom 1215

Band Detection

**Automatic Detection:**	Yes
**Minimum Band Slope:**	183
**Median Filter:**	5
**Percentage Maximum Peak:**	0
**Maximum Peak Measure:**	Gel
**Edge Detection Method:**	Fixed Width
**Fixed Width pixels:**	17
**Band Positions Edited:**	Yes

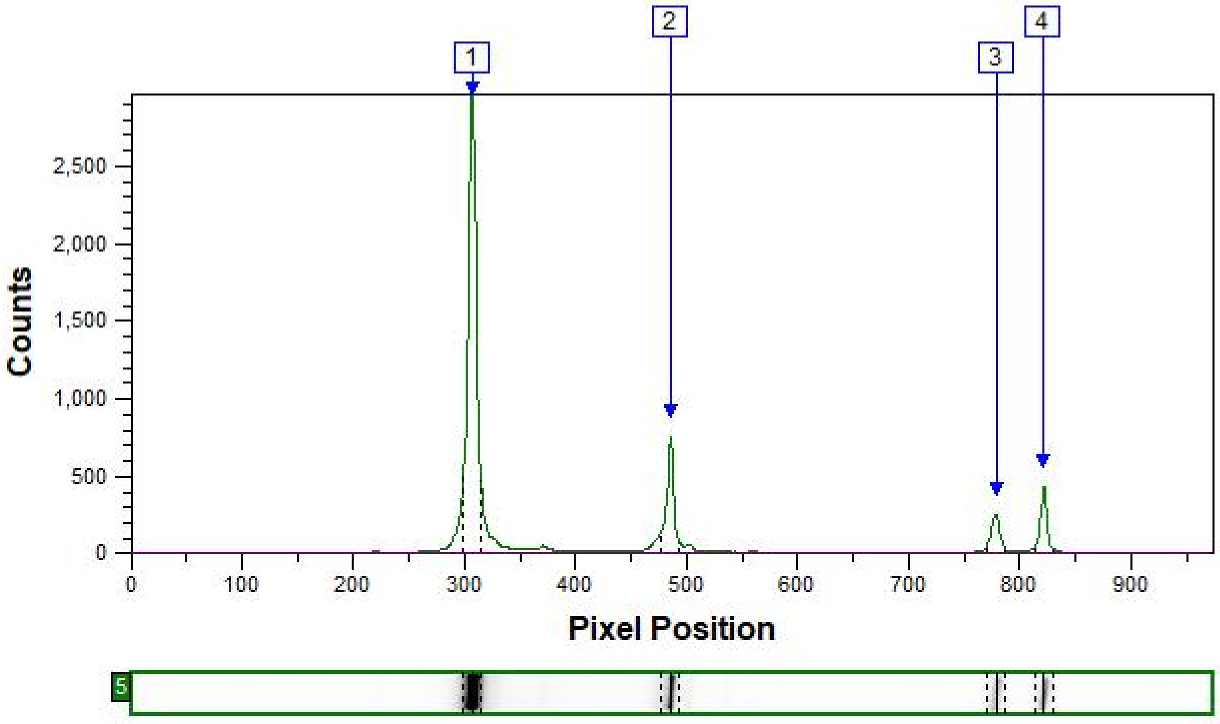

Band Table

Band No	Volume	Vol+BkGnd	Band %
1	976570.35	979872.94	69.984
2	216076.31	219378.90	15.485
3	90481.10	93783.70	6.484
4	112299.09	115601.69	8.048

ImageQuant TL

Lane Data Report: rw 2025_10_22 p8 timecourse t18h-[phosphor].gel, Lane 6

Summary

**Name:**	Lane 6

Background

**Background Type:**	Image Rectangle
**Rectangle:**	Left 398, Right 1202, Top 1059, Bottom 1215

Band Detection

**Automatic Detection:**	Yes
**Minimum Band Slope:**	183
**Median Filter:**	5
**Percentage Maximum Peak:**	0
**Maximum Peak Measure:**	Gel
**Edge Detection Method:**	Fixed Width
**Fixed Width pixels:**	17
**Band Positions Edited:**	Yes

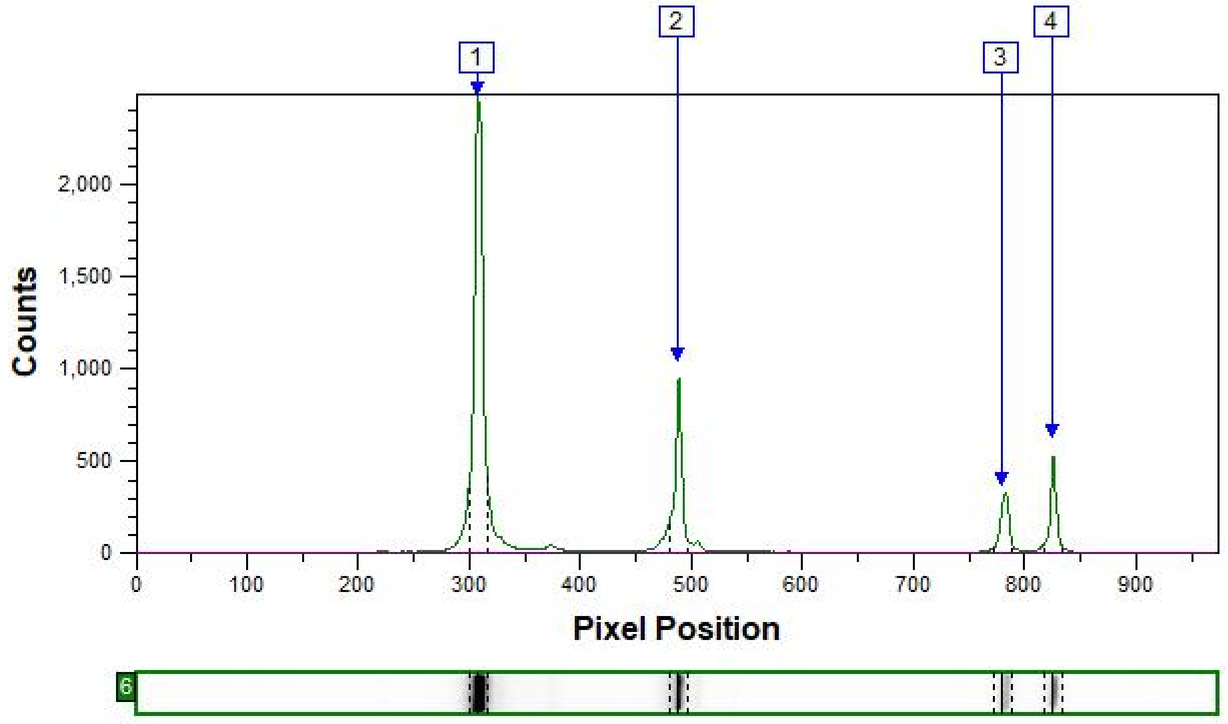

Band Table

Band No	Volume	Vol+BkGnd	Band %
1	838825.54	842128.13	63.420
2	250771.10	254073.69	18.960
3	106321.55	109624.14	8.039
4	126735.10	130037.69	9.582

ImageQuant TL

Lane Data Report: rw 2025_10_22 p8 timecourse t18h-[phosphor].gel, Lane 7

Summary

**Name:**	Lane 7

Background

**Background Type:**	Image Rectangle
**Rectangle:**	Left 398, Right 1202, Top 1059, Bottom 1215

Band Detection

**Automatic Detection:**	Yes
**Minimum Band Slope:**	183
**Median Filter:**	5
**Percentage Maximum Peak:**	0
**Maximum Peak Measure:**	Gel
**Edge Detection Method:**	Fixed Width
**Fixed Width pixels:**	17
**Band Positions Edited:**	No

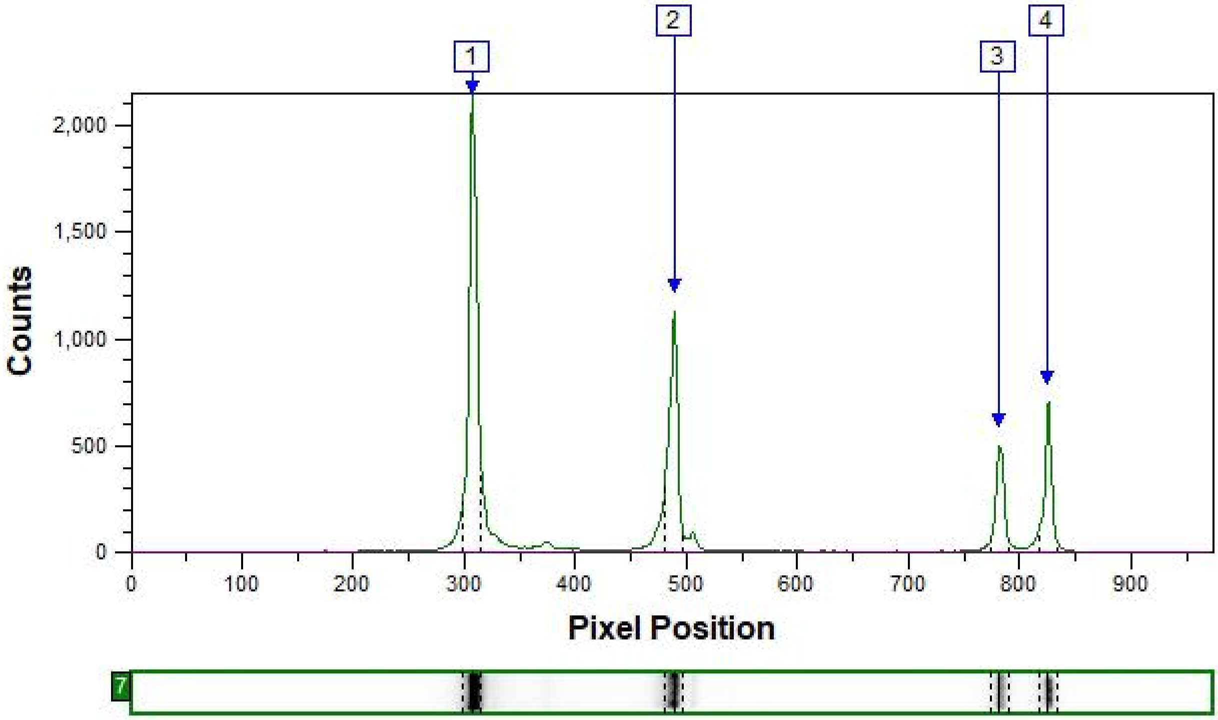

Band Table

Band No	Volume	Vol+BkGnd	Band %
1	674181.38	677483.97	48.197
2	372467.16	375769.75	26.628
3	161934.58	165237.17	11.577
4	190212.79	193515.38	13.598

Lane Data Report: rw 2025_10_22 p8 timecourse t18h-[phosphor].gel, Lane 8

Summary

**Name:**	Lane 8

Background

**Background Type:**	Image Rectangle
**Rectangle:**	Left 398, Right 1202, Top 1059, Bottom 1215

Band Detection

**Automatic Detection:**	Yes
**Minimum Band Slope:**	183
**Median Filter:**	5
**Percentage Maximum Peak:**	0
**Maximum Peak Measure:**	Gel
**Edge Detection Method:**	Fixed Width
**Fixed Width pixels:**	17
**Band Positions Edited:**	No

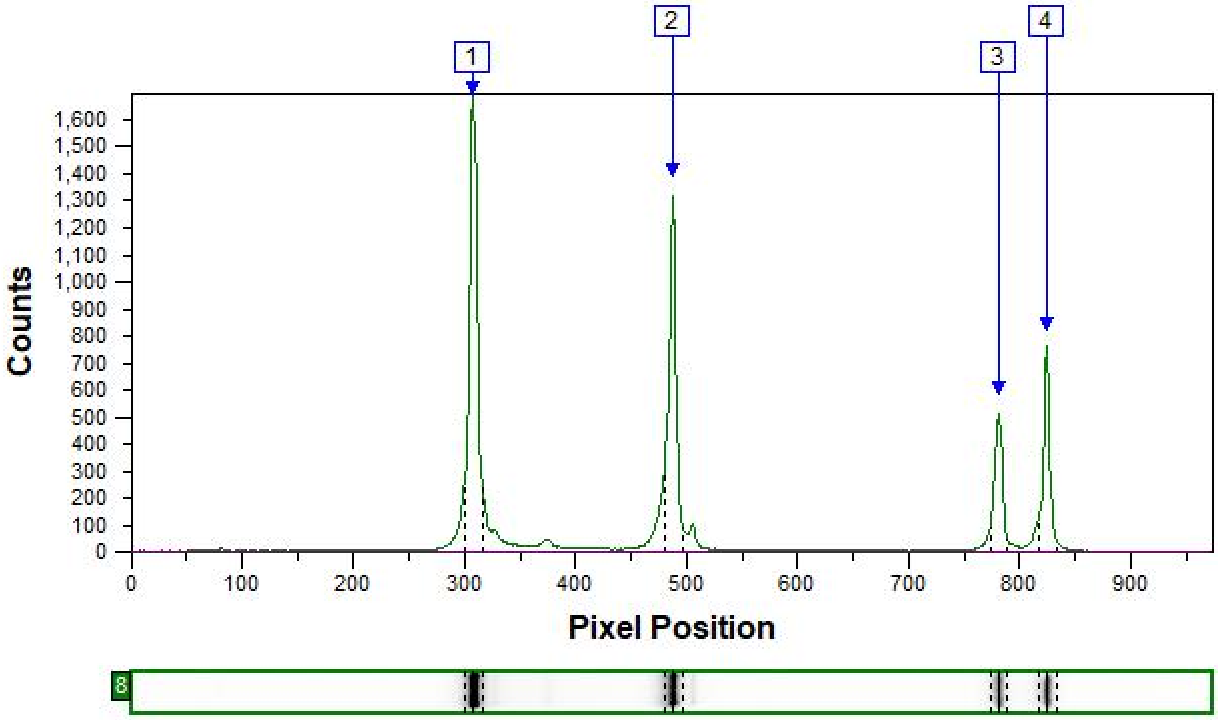

Band Table

Band No	Volume	Vol+BkGnd	Band %
1	533501.74	536804.33	41.525
2	387449.97	390752.56	30.157
3	168549.29	171851.89	13.119
4	195268.73	198571.32	15.199

ImageQuant TL

Lane Data Report: rw 2025_10_22 p8 timecourse t18h-[phosphor].gel, Lane 9

Summary

**Name:**	Lane 9

Background

**Background Type:**	Image Rectangle
**Rectangle:**	Left 398, Right 1202, Top 1059, Bottom 1215

Band Detection

**Automatic Detection:**	Yes
**Minimum Band Slope:**	183
**Median Filter:**	5
**Percentage Maximum Peak:**	0
**Maximum Peak Measure:**	Gel
**Edge Detection Method:**	Fixed Width
**Fixed Width pixels:**	17
**Band Positions Edited:**	No

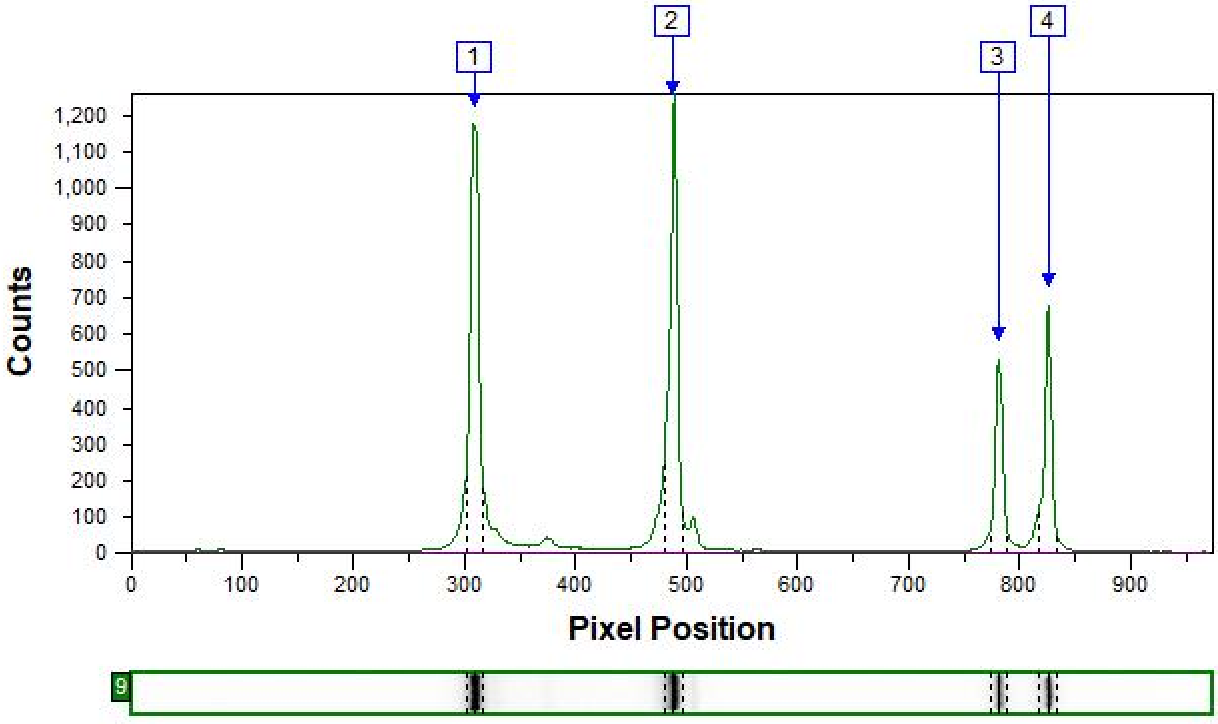

Band Table

Band No	Volume	Vol+BkGnd	Band %
1	416237.45	419540.04	36.364
2	382120.95	385423.54	33.384
3	161588.36	164890.95	14.117
4	184687.52	187990.11	16.135

ImageQuant TL

Lane Data Report: rw 2025_10_22 p8 timecourse t18h-[phosphor].gel, Lane 10

Summary

**Name:**	Lane 10

Background

**Background Type:**	Image Rectangle
**Rectangle:**	Left 398, Right 1202, Top 1059, Bottom 1215

Band Detection

**Automatic Detection:**	Yes
**Minimum Band Slope:**	183
**Median Filter:**	5
**Percentage Maximum Peak:**	0
**Maximum Peak Measure:**	Gel
**Edge Detection Method:**	Fixed Width
**Fixed Width pixels:**	17
**Band Positions Edited:**	No

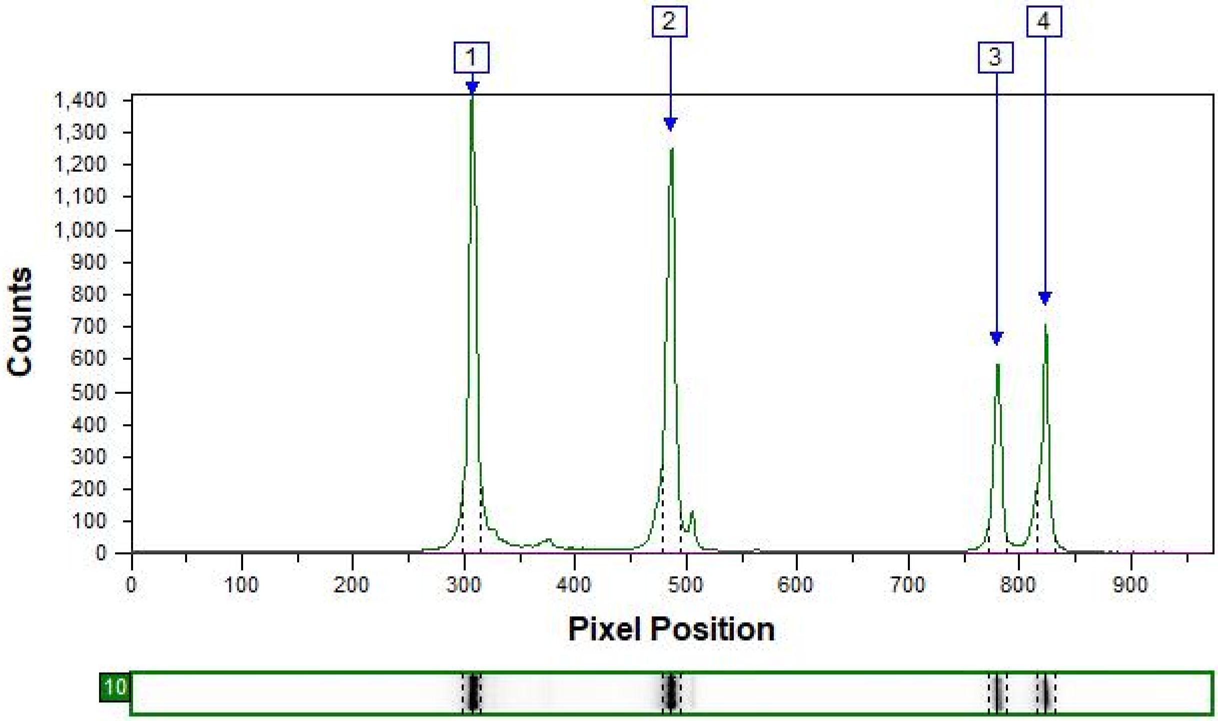

Band Table

Band No	Volume	Vol+BkGnd	Band %
1	442392.92	445695.51	34.763
2	440051.00	443353.59	34.579
3	187203.84	190506.43	14.710
4	202939.56	206242.16	15.947

ImageQuant TL

Lane Data Report: rw 2025_10_22 p8 timecourse t18h-[phosphor].gel, Lane 11

Summary

**Name:**	Lane 11

Background

**Background Type:**	Image Rectangle
**Rectangle:**	Left 398, Right 1202, Top 1059, Bottom 1215

Band Detection

**Automatic Detection:**	Yes
**Minimum Band Slope:**	183
**Median Filter:**	5
**Percentage Maximum Peak:**	0
**Maximum Peak Measure:**	Gel
**Edge Detection Method:**	Fixed Width
**Fixed Width pixels:**	17
**Band Positions Edited:**	Yes

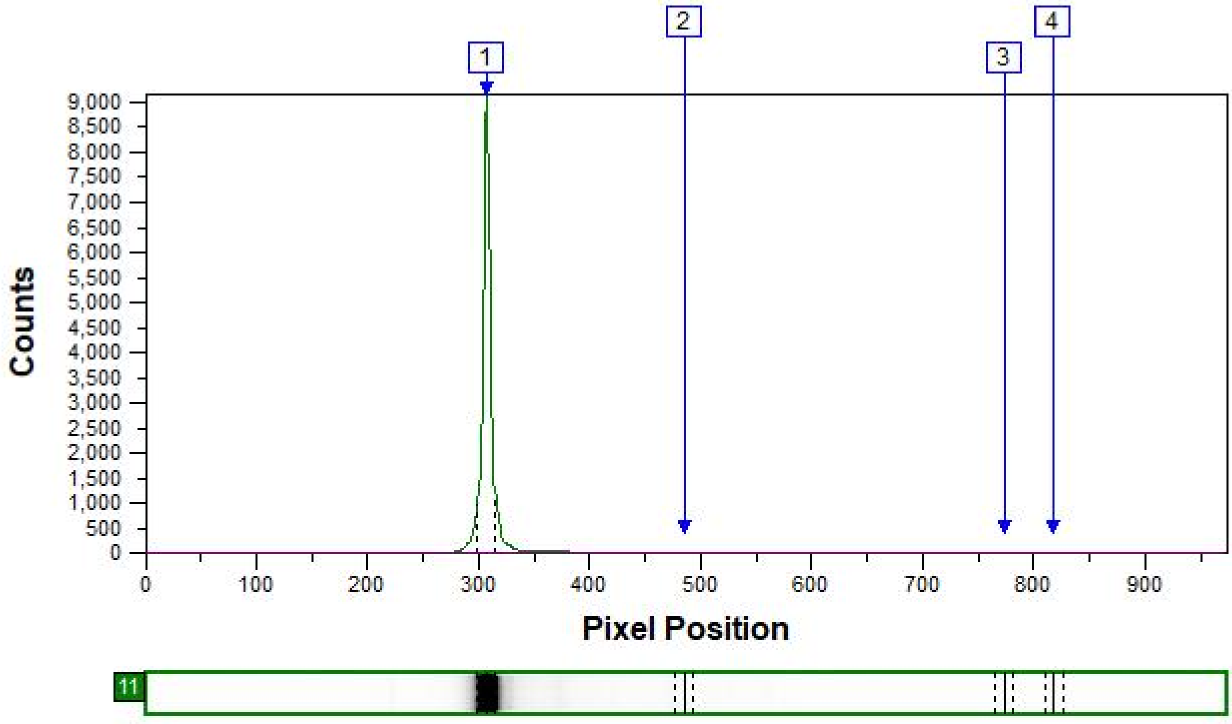

Band Table

Band No	Volume	Vol+BkGnd	Band %
1	2524122.46	2527425.05	99.141
2	12001.52	15304.11	0.471
3	3118.11	6420.70	0.122
4	6750.67	10053.26	0.265

ImageQuant TL

Lane Data Report: rw 2025_10_22 p8 timecourse t18h-[phosphor].gel, Lane 12

Summary

**Name:**	Lane 12

Background

**Background Type:**	Image Rectangle
**Rectangle:**	Left 398, Right 1202, Top 1059, Bottom 1215

Band Detection

**Automatic Detection:**	Yes
**Minimum Band Slope:**	183
**Median Filter:**	5
**Percentage Maximum Peak:**	0
**Maximum Peak Measure:**	Gel
**Edge Detection Method:**	Fixed Width
**Fixed Width pixels:**	17
**Band Positions Edited:**	Yes

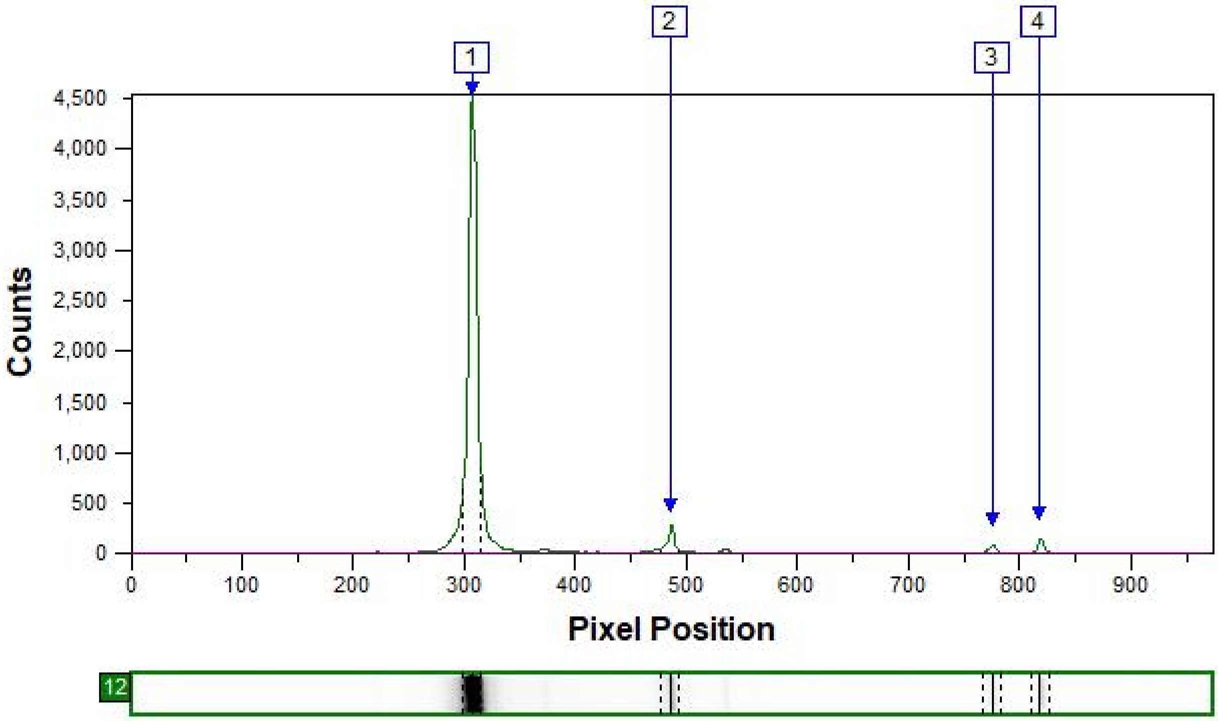

Band Table

Band No	Volume	Vol+BkGnd	Band %
1	1542798.72	1546101.31	91.254
2	82361.50	85664.09	4.872
3	28791.97	32094.56	1.703
4	36714.30	40016.89	2.172

Lane Data Report: rw 2025_10_22 p8 timecourse t18h-[phosphor].gel, Lane 13

Summary

**Name:**	Lane 13

Background

**Background Type:**	Image Rectangle
**Rectangle:**	Left 398, Right 1202, Top 1059, Bottom 1215

Band Detection

**Automatic Detection:**	Yes
**Minimum Band Slope:**	183
**Median Filter:**	5
**Percentage Maximum Peak:**	0
**Maximum Peak Measure:**	Gel
**Edge Detection Method:**	Fixed Width
**Fixed Width pixels:**	17
**Band Positions Edited:**	Yes

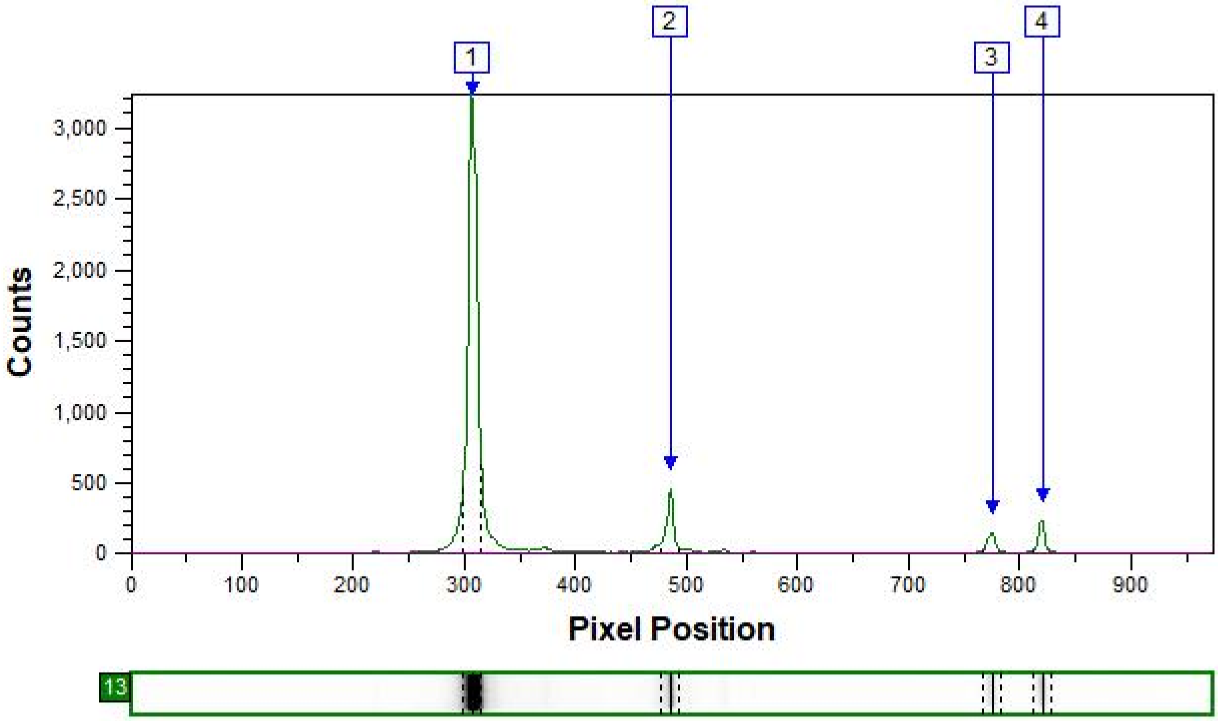

Band Table

Band No	Volume	Vol+BkGnd	Band %
1	1156671.05	1159973.64	83.271
2	125485.13	128787.72	9.034
3	48116.53	51419.12	3.464
4	58767.22	62069.82	4.231

ImageQuant TL

Lane Data Report: rw 2025_10_22 p8 timecourse t18h-[phosphor].gel, Lane 14

Summary

**Name:**	Lane 14

Background

**Background Type:**	Image Rectangle
**Rectangle:**	Left 398, Right 1202, Top 1059, Bottom 1215

Band Detection

**Automatic Detection:**	Yes
**Minimum Band Slope:**	183
**Median Filter:**	5
**Percentage Maximum Peak:**	0
**Maximum Peak Measure:**	Gel
**Edge Detection Method:**	Fixed Width
**Fixed Width pixels:**	17
**Band Positions Edited:**	Yes

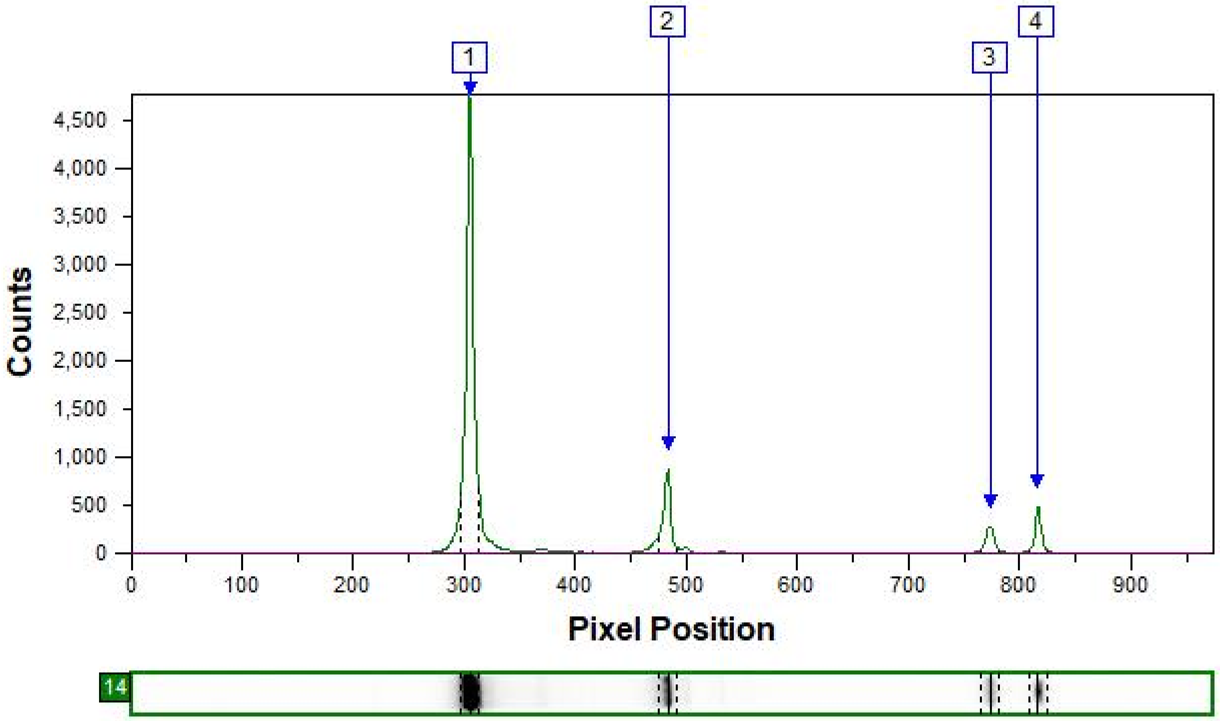

Band Table

Band No	Volume	Vol+BkGnd	Band %
1	1422968.62	1426271.22	74.977
2	249309.48	252612.07	13.136
3	99818.49	103121.08	5.260
4	125770.42	129073.02	6.627

ImageQuant TL

Lane Data Report: rw 2025_10_22 p8 timecourse t18h-[phosphor].gel, Lane 15

Summary

**Name:**	Lane 15

Background

**Background Type:**	Image Rectangle
**Rectangle:**	Left 398, Right 1202, Top 1059, Bottom 1215

Band Detection

**Automatic Detection:**	Yes
**Minimum Band Slope:**	183
**Median Filter:**	5
**Percentage Maximum Peak:**	0
**Maximum Peak Measure:**	Gel
**Edge Detection Method:**	Fixed Width
**Fixed Width pixels:**	17
**Band Positions Edited:**	No

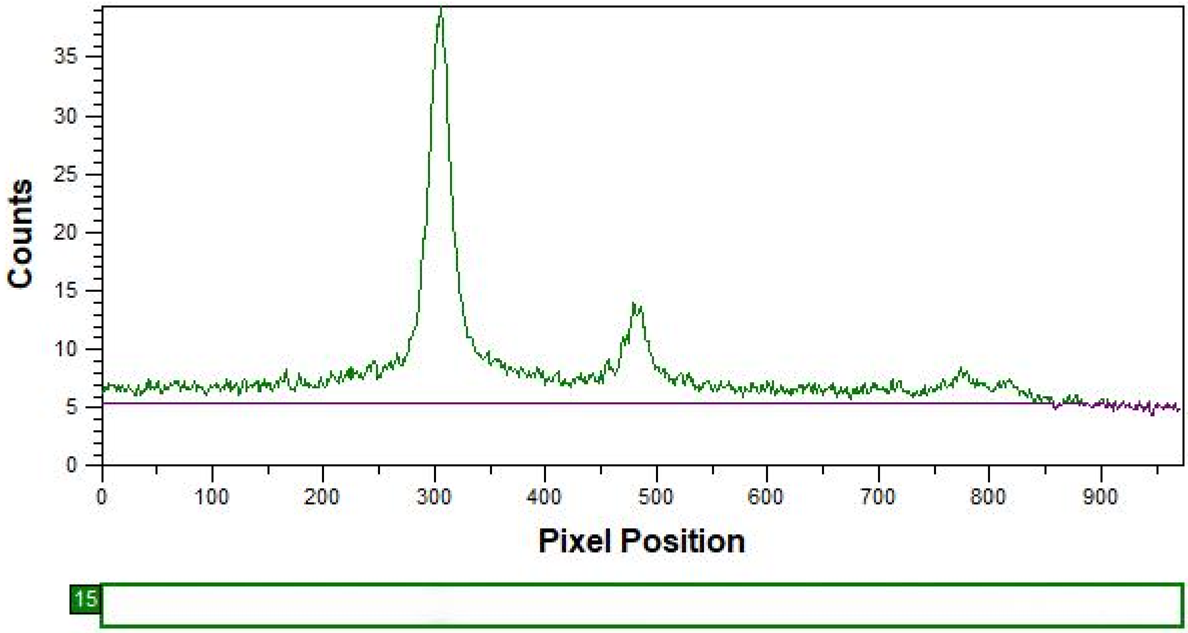

Band Table

Lane Data Report: rw 2025_10_22 p8 timecourse t18h-[phosphor].gel, Lane 16

Summary

**Name:**	Lane 16

Background

**Background Type:**	Image Rectangle
**Rectangle:**	Left 398, Right 1202, Top 1059, Bottom 1215

Band Detection

**Automatic Detection:**	Yes
**Minimum Band Slope:**	183
**Median Filter:**	5
**Percentage Maximum Peak:**	0
**Maximum Peak Measure:**	Gel
**Edge Detection Method:**	Fixed Width
**Fixed Width pixels:**	17
**Band Positions Edited:**	Yes

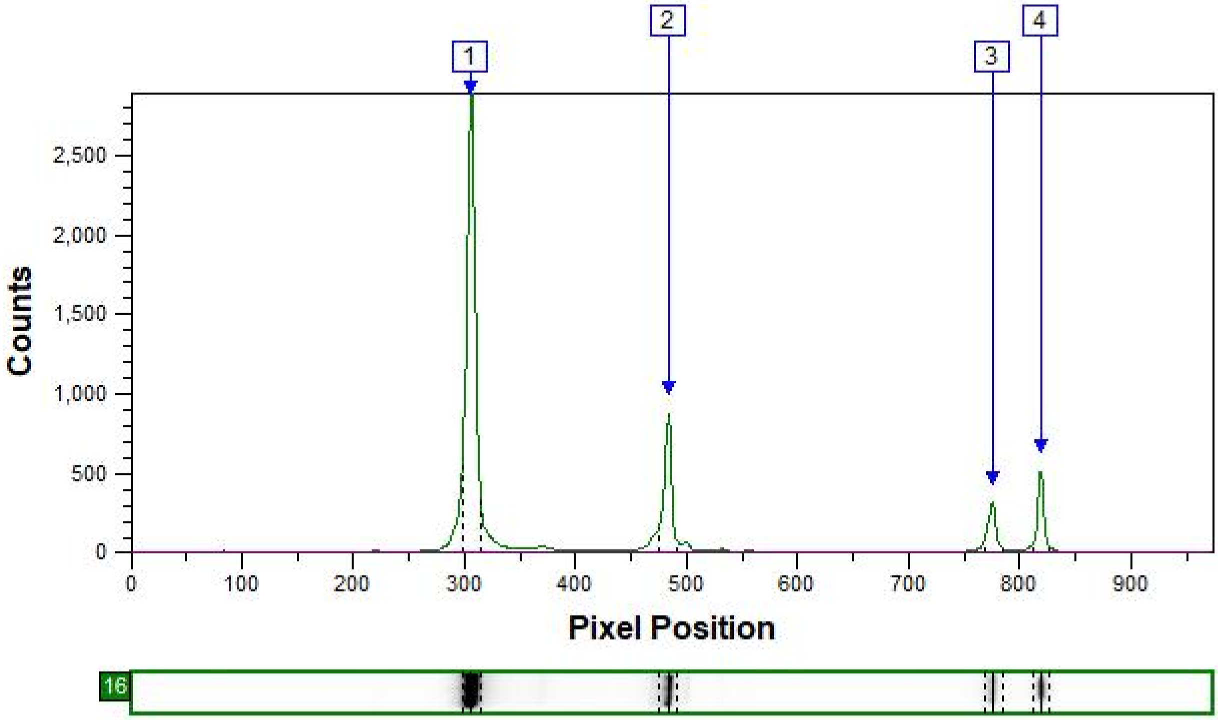

Band Table

Band No	Volume	Vol+BkGnd	Band %
1	992422.34	995724.93	67.250
2	256384.97	259687.57	17.374
3	101240.96	104543.55	6.860
4	125670.02	128972.61	8.516

IQTL Report version 8.1
Time of Report: 3:42 PM 10/22/2025 (Eastern Daylight Time)

ImageQuant TL

Lane Data Report: rw 2025_10_22 p8 timecourse t18h-[phosphor].gel, Lane 17

Summary

**Name:**	Lane 17

Background

**Background Type:**	Image Rectangle
**Rectangle:**	Left 398, Right 1202, Top 1059, Bottom 1215

Band Detection

**Automatic Detection:**	Yes
**Minimum Band Slope:**	183
**Median Filter:**	5
**Percentage Maximum Peak:**	0
**Maximum Peak Measure:**	Gel
**Edge Detection Method:**	Fixed Width
**Fixed Width pixels:**	17
**Band Positions Edited:**	No

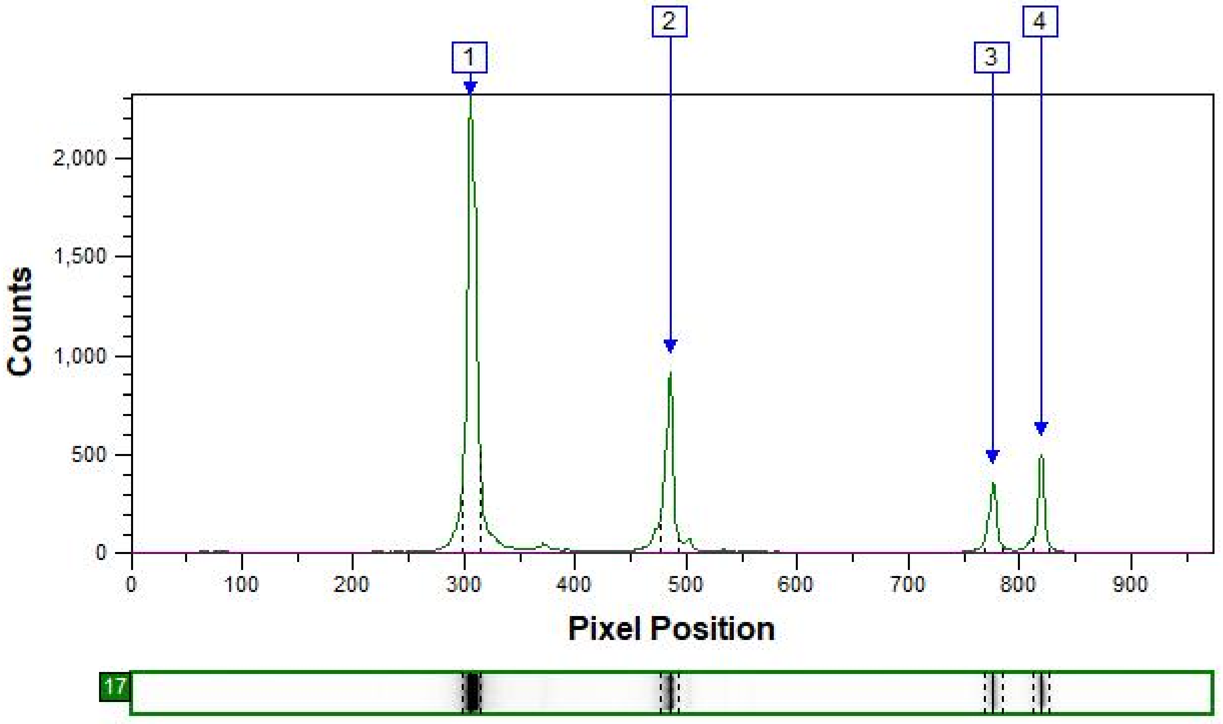

Band Table

Band No	Volume	Vol+BkGnd	Band %
1	813581.31	816883.90	60.793
2	282207.06	285509.65	21.087
3	113176.65	116479.24	8.457
4	129323.01	132625.60	9.663

ImageQuant TL

Lane Data Report: rw 2025_10_22 p8 timecourse t18h-[phosphor].gel, Lane 18

Summary

**Name:**	Lane 18

Background

**Background Type:**	Image Rectangle
**Rectangle:**	Left 398, Right 1202, Top 1059, Bottom 1215

Band Detection

**Automatic Detection:**	Yes
**Minimum Band Slope:**	183
**Median Filter:**	5
**Percentage Maximum Peak:**	0
**Maximum Peak Measure:**	Gel
**Edge Detection Method:**	Fixed Width
**Fixed Width pixels:**	17
**Band Positions Edited:**	No

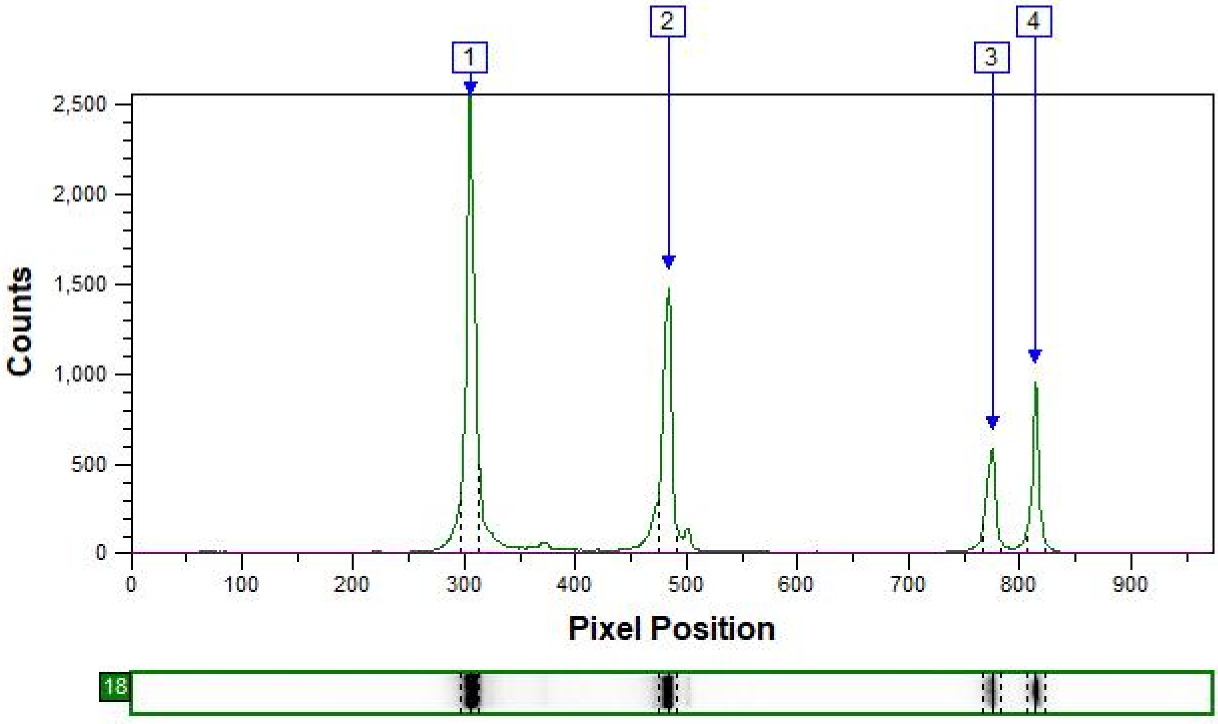

Band Table

Band No	Volume	Vol+BkGnd	Band %
1	824858.57	828161.16	46.030
2	506690.95	509993.54	28.275
3	207981.44	211284.03	11.606
4	252462.15	255764.74	14.088

ImageQuant TL

Lane Data Report: rw 2025_10_22 p8 timecourse t18h-[phosphor].gel, Lane 19

Summary

**Name:**	Lane 19

Background

**Background Type:**	Image Rectangle
**Rectangle:**	Left 398, Right 1202, Top 1059, Bottom 1215

Band Detection

**Automatic Detection:**	Yes
**Minimum Band Slope:**	183
**Median Filter:**	5
**Percentage Maximum Peak:**	0
**Maximum Peak Measure:**	Gel
**Edge Detection Method:**	Fixed Width
**Fixed Width pixels:**	17
**Band Positions Edited:**	No

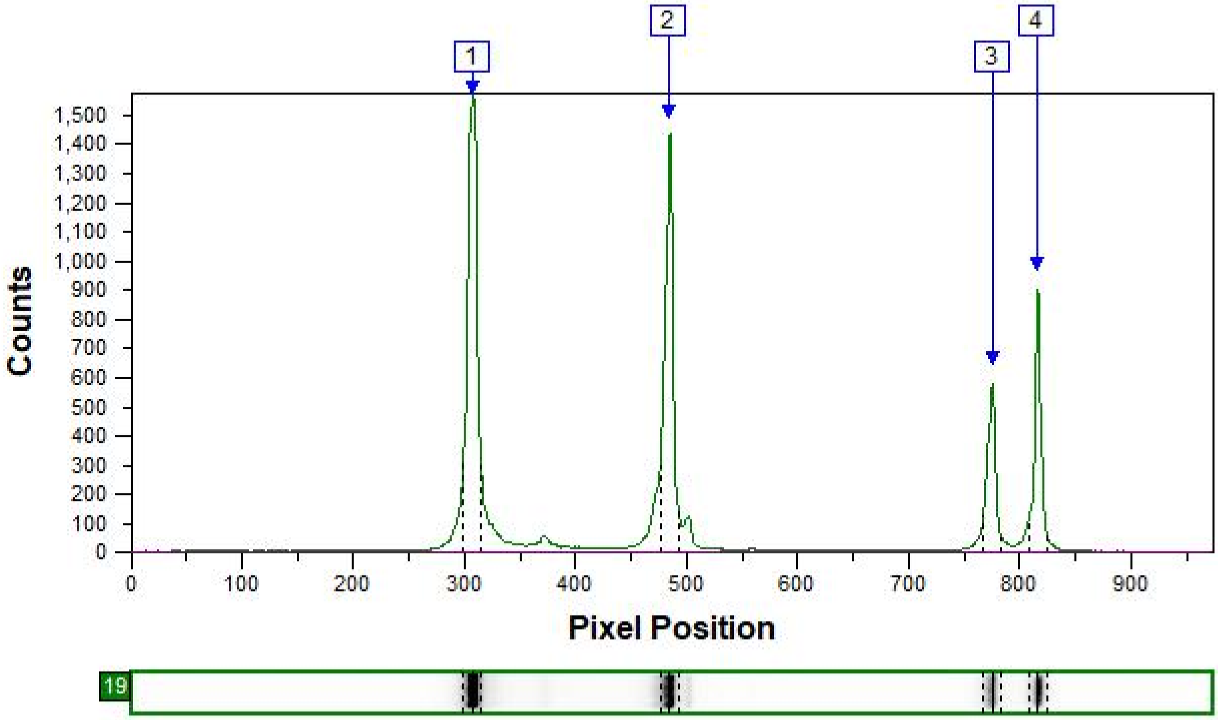

Band Table

Band No	Volume	Vol+BkGnd	Band %
1	591807.39	595109.98	40.268
2	462770.05	466072.64	31.488
3	189672.83	192975.42	12.906
4	225404.38	228706.97	15.337

Lane Data Report: rw 2025_10_22 p8 timecourse t18h-[phosphor].gel, Lane 20

Summary

**Name:**	Lane 20

Background

**Background Type:**	Image Rectangle
**Rectangle:**	Left 398, Right 1202, Top 1059, Bottom 1215

Band Detection

**Automatic Detection:**	Yes
**Minimum Band Slope:**	183
**Median Filter:**	5
**Percentage Maximum Peak:**	0
**Maximum Peak Measure:**	Gel
**Edge Detection Method:**	Fixed Width
**Fixed Width pixels:**	17
**Band Positions Edited:**	No

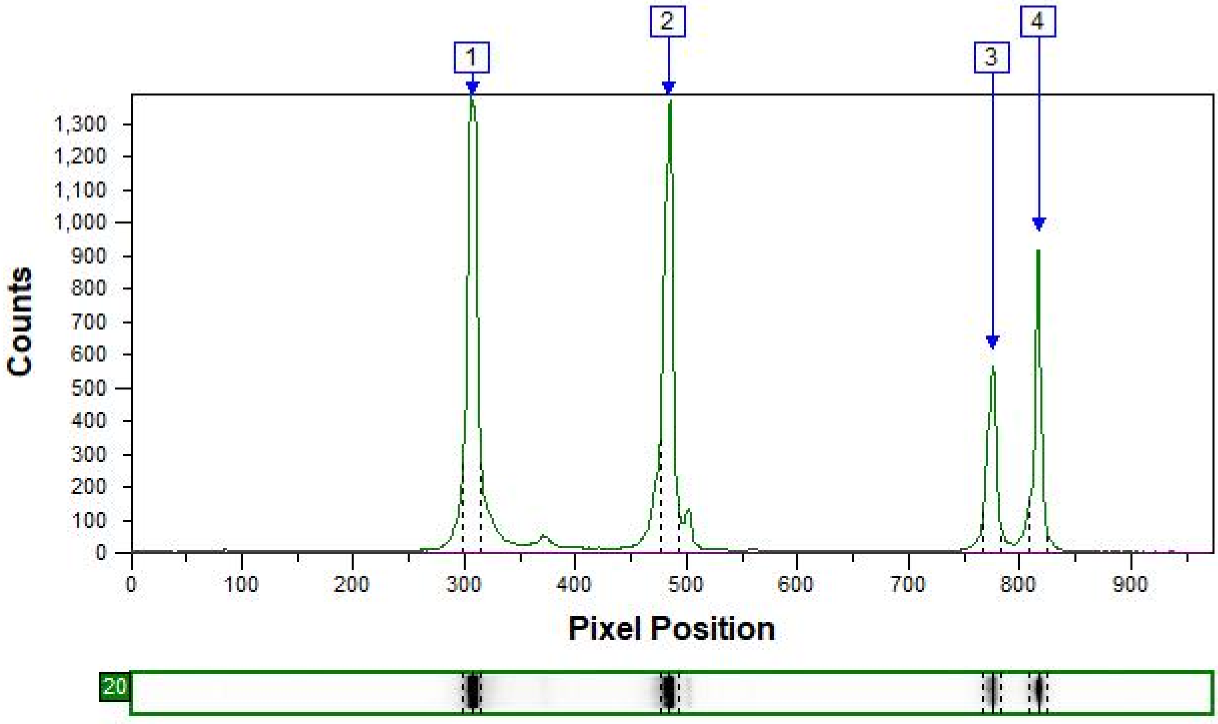

Band Table

Band No	Volume	Vol+BkGnd	Band %
1	542188.97	545491.57	36.697
2	494392.62	497695.21	33.462
3	205459.07	208761.67	13.906
4	235432.03	238734.62	15.935

IQTL Report version 8.1
Time of Report: 3:42 PM 10/22/2025 (Eastern Daylight Time)

Lane Data Report: rw 2025_10_22 p8 timecourse t18h-[phosphor].gel, Lane 21

Summary

**Name:**	Lane 21

Background

**Background Type:**	Image Rectangle
**Rectangle:**	Left 398, Right 1202, Top 1059, Bottom 1215

Band Detection

**Automatic Detection:**	Yes
**Minimum Band Slope:**	183
**Median Filter:**	5
**Percentage Maximum Peak:**	0
**Maximum Peak Measure:**	Gel
**Edge Detection Method:**	Fixed Width
**Fixed Width pixels:**	17
**Band Positions Edited:**	No

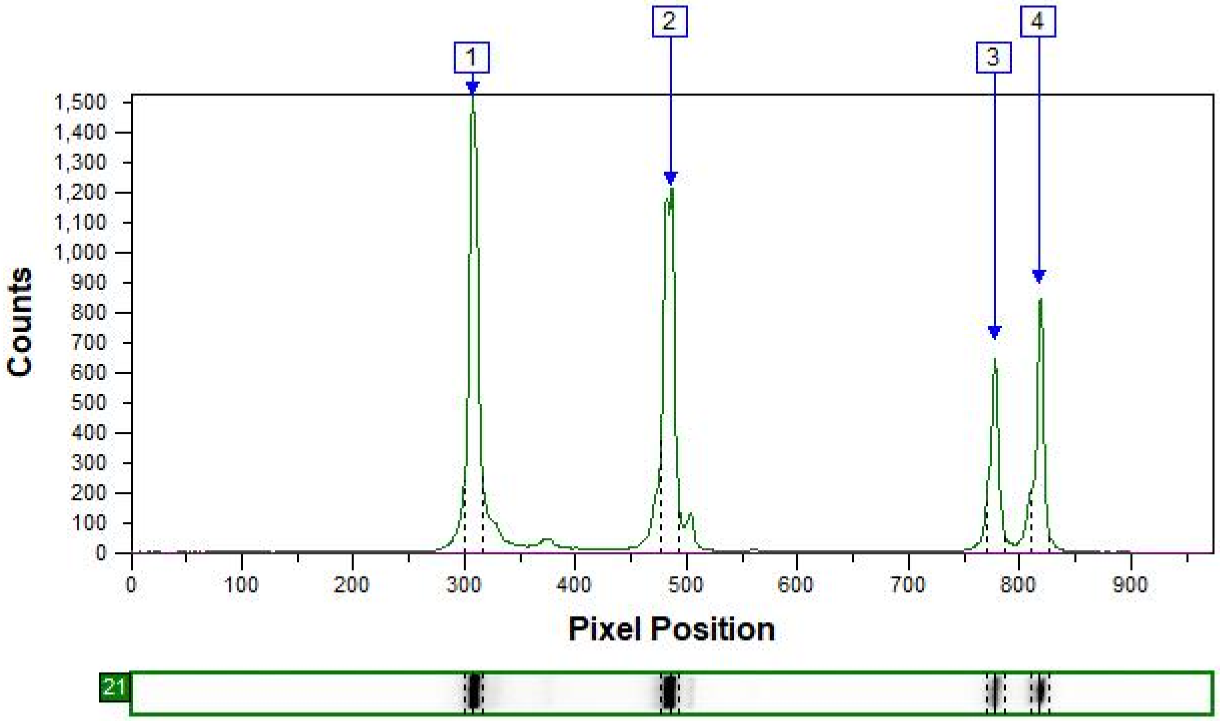

Band Table

Band No	Volume	Vol+BkGnd	Band %
1	533326.32	536628.91	35.425
2	517097.09	520399.68	34.347
3	215539.17	218841.76	14.317
4	239556.88	242859.47	15.912
