# Supplementary material for: PCanPIE: A group I intron platform for efficient circRNA synthesis at ambient temperatures
Source: RNA. Author manuscript; Available in PMC 2026 Nov 9. (PMC13580324; doi:10.1261/rna.081078.126)
Supplement: Supplementary file 1 [file NIHMS2209016-supplement-Data_source_files.zip › 14. Data source files/Fig. 3 Data/2025_10_23 P5 timecourse/RW 2025_10_23 P5 timecourse analysis-[Phosphor]gel_AllLanes.pdf]

ImageQuant TL

Analysis Report: rw 2025_10_23 p5 timecourse analysis-[phosphor].gel

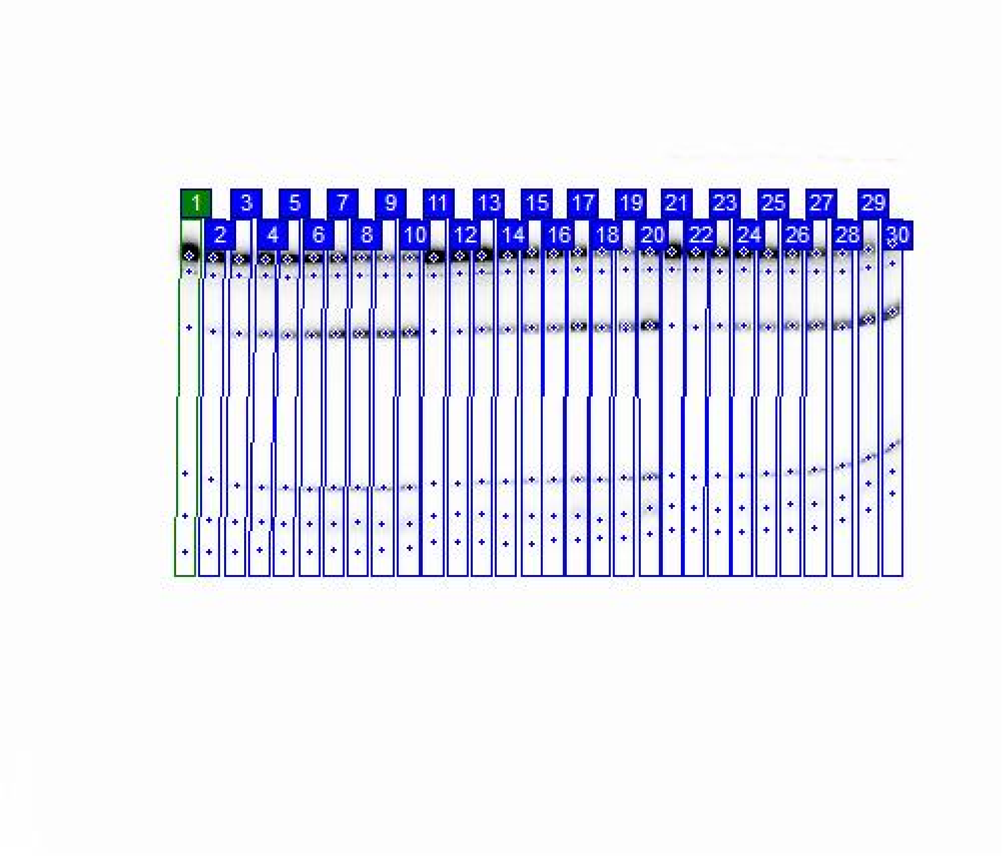

Lane Table

Lane	Bands	Band Volume	Lane Volume
Lane 1	6	1699932.05	3021701.15
Lane 2	6	1150755.06	1871669.73
Lane 3	6	1070865.83	1689239.52
Lane 4	6	1544976.94	2380597.03
Lane 5	6	1204258.33	1834646.59
Lane 6	6	1402421.73	2157743.02
Lane 7	6	1418309.74	2095437.39
Lane 8	6	1210434.35	1849610.71
Lane 9	6	1093504.89	1622853.28
Lane 10	6	1275361.80	1889327.85
Lane 11	6	1785955.71	2740144.97
Lane 12	6	1419203.26	2192308.09
Lane 13	6	2160398.28	3235687.68
Lane 14	6	1135796.56	1781039.58
Lane 15	6	1226678.67	1960900.33
Lane 16	6	1192807.90	1845356.76
Lane 17	6	1419156.32	2204323.20
Lane 18	6	842736.68	1270201.26
Lane 19	7	894606.43	1314798.89
Lane 20	6	1741383.39	2641870.61
Lane 21	6	1627172.94	2622370.71
Lane 22	6	1214126.42	1969543.54
Lane 23	6	1053353.52	1715059.35

ImageQuant TL

Lane	Bands	Band Volume	Lane Volume
Lane 24	6	1246760.70	2014243.87
Lane 25	6	1214261.61	1935155.77
Lane 26	6	1166314.59	1899775.28
Lane 27	6	1172151.78	1880135.24
Lane 28	6	920043.72	1572630.48
Lane 29	6	1148006.43	1970322.47
Lane 30	6	1027087.42	1880591.89

ImageQuant TL

Lane Data Report: rw 2025_10_23 p5 timecourse analysis-[phosphor].gel, Lane 1

Summary

**Name:**	Lane 1

Background

**Background Type:**	Image Rectangle
**Rectangle:**	Left 313, Right 1075, Top 987, Bottom 1077

Band Detection

**Automatic Detection:**	Yes
**Minimum Band Slope:**	182
**Median Filter:**	5
**Percentage Maximum Peak:**	0
**Maximum Peak Measure:**	Gel
**Edge Detection Method:**	Fixed Width
**Fixed Width pixels:**	17
**Band Positions Edited:**	Yes

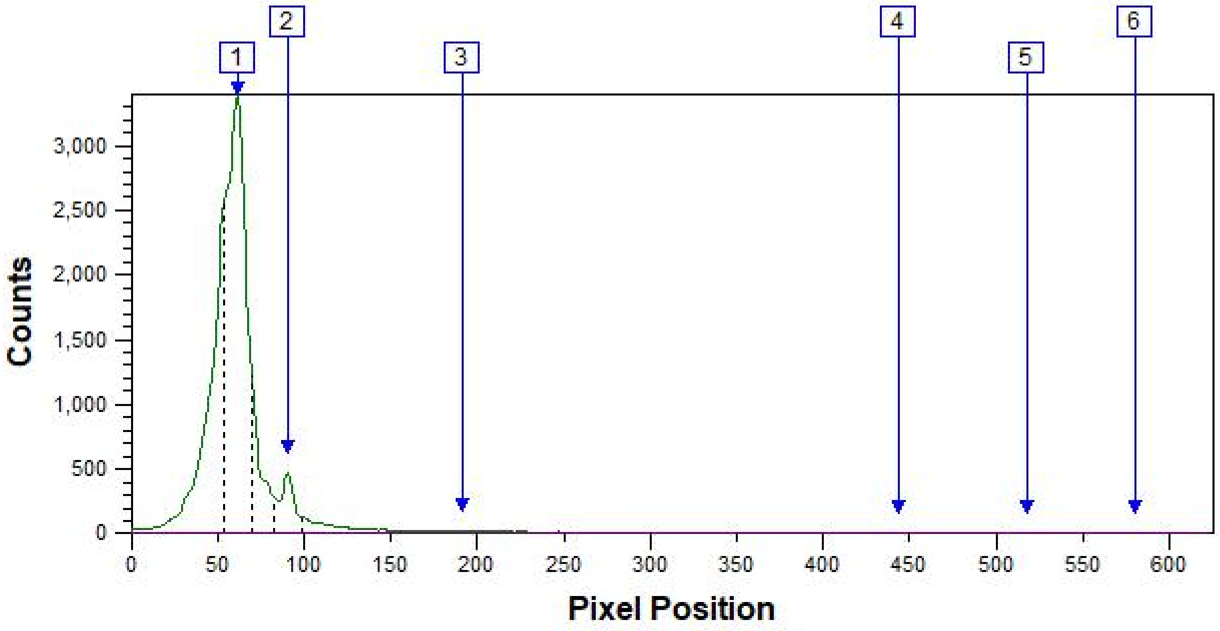

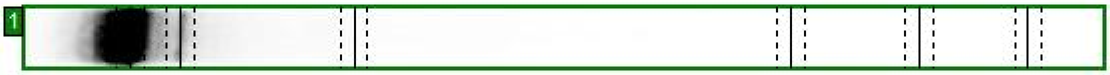

Band Table

Band No	Volume	Vol+BkGnd	Band %
1	1517828.81	1520018.53	89.288
2	168485.09	170675.13	9.911
3	11358.86	13550.86	0.668
4	1534.22	3726.81	0.090
5	408.48	2603.36	0.024
6	316.58	2511.11	0.019

ImageQuant TL

Lane Data Report: rw 2025_10_23 p5 timecourse analysis-[phosphor].gel, Lane 2

Summary

**Name:**	Lane 2

Background

**Background Type:**	Image Rectangle
**Rectangle:**	Left 313, Right 1075, Top 987, Bottom 1077

Band Detection

**Automatic Detection:**	Yes
**Minimum Band Slope:**	182
**Median Filter:**	5
**Percentage Maximum Peak:**	0
**Maximum Peak Measure:**	Gel
**Edge Detection Method:**	Fixed Width
**Fixed Width pixels:**	17
**Band Positions Edited:**	Yes

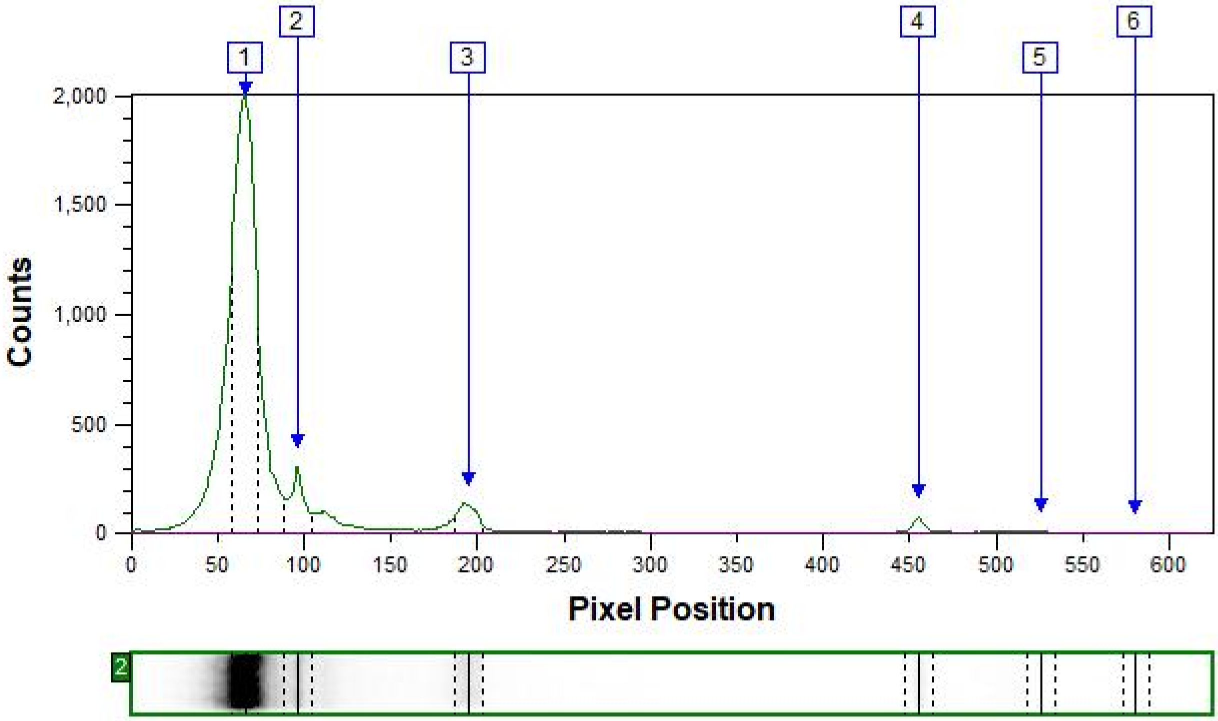

Band Table

Band No	Volume	Vol+BkGnd	Band %
1	958184.47	960374.19	83.266
2	104584.55	106774.48	9.088
3	62247.44	64438.58	5.409
4	21382.04	23573.30	1.858
5	4197.34	6386.74	0.365
6	159.22	2320.17	0.014

ImageQuant TL

Lane Data Report: rw 2025_10_23 p5 timecourse analysis-[phosphor].gel, Lane 3

Summary

**Name:**	Lane 3

Background

**Background Type:**	Image Rectangle
**Rectangle:**	Left 313, Right 1075, Top 987, Bottom 1077

Band Detection

**Automatic Detection:**	Yes
**Minimum Band Slope:**	182
**Median Filter:**	5
**Percentage Maximum Peak:**	0
**Maximum Peak Measure:**	Gel
**Edge Detection Method:**	Fixed Width
**Fixed Width pixels:**	17
**Band Positions Edited:**	Yes

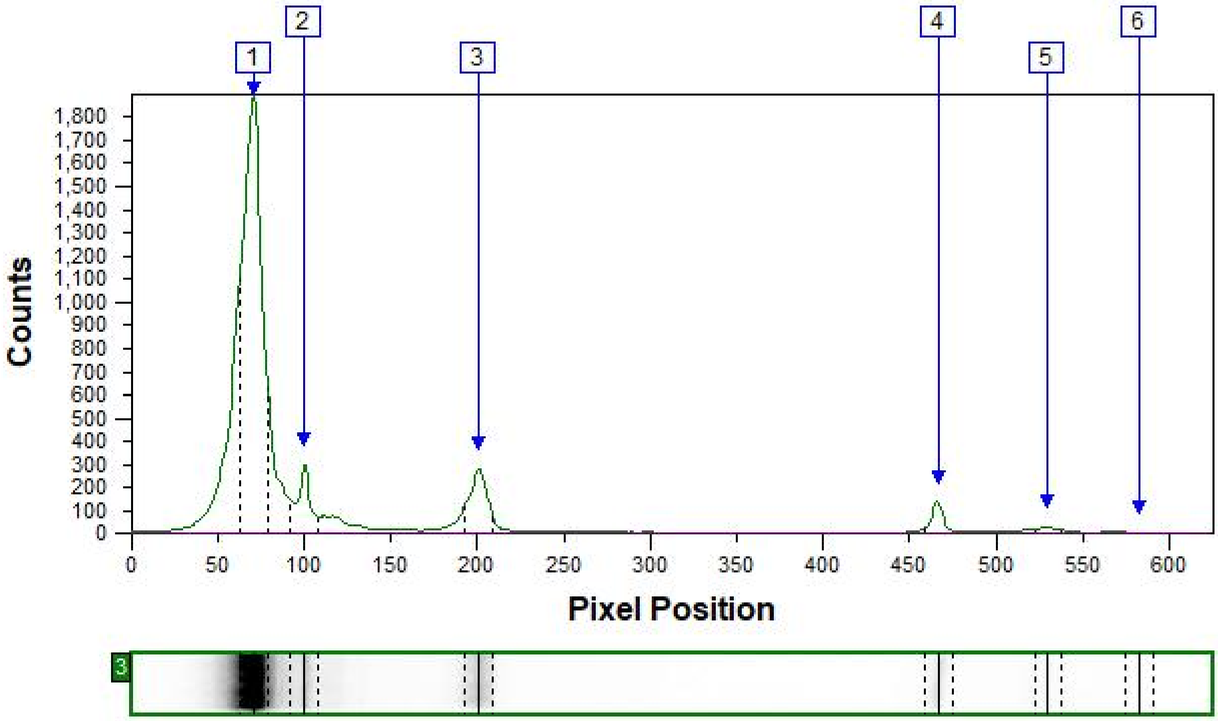

Band Table

Band No	Volume	Vol+BkGnd	Band %
1	812977.52	815166.51	75.918
2	93287.50	95476.48	8.711
3	112101.71	114290.69	10.468
4	38903.71	41092.69	3.633
5	12600.21	14789.19	1.177
6	995.18	3184.16	0.093

ImageQuant TL

Lane Data Report: rw 2025_10_23 p5 timecourse analysis-[phosphor].gel, Lane 4

Summary

**Name:**	Lane 4

Background

**Background Type:**	Image Rectangle
**Rectangle:**	Left 313, Right 1075, Top 987, Bottom 1077

Band Detection

**Automatic Detection:**	Yes
**Minimum Band Slope:**	182
**Median Filter:**	5
**Percentage Maximum Peak:**	0
**Maximum Peak Measure:**	Gel
**Edge Detection Method:**	Fixed Width
**Fixed Width pixels:**	17
**Band Positions Edited:**	Yes

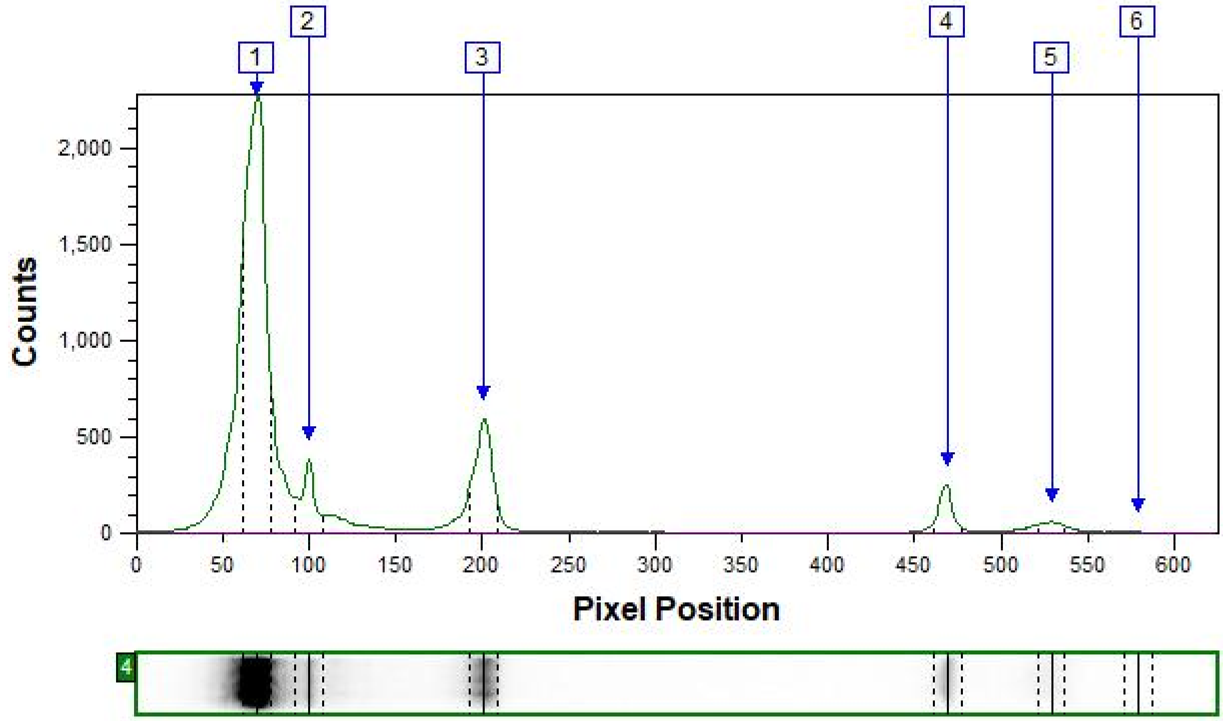

Band Table

Band No	Volume	Vol+BkGnd	Band %
1	1062890.74	1065079.72	68.797
2	124207.76	126396.74	8.039
3	241140.14	243329.12	15.608
4	81585.65	83774.63	5.281
5	30642.35	32831.33	1.983
6	4510.30	6699.28	0.292

ImageQuant TL

Lane Data Report: rw 2025_10_23 p5 timecourse analysis-[phosphor].gel, Lane 5

Summary

**Name:**	Lane 5

Background

**Background Type:**	Image Rectangle
**Rectangle:**	Left 313, Right 1075, Top 987, Bottom 1077

Band Detection

**Automatic Detection:**	Yes
**Minimum Band Slope:**	182
**Median Filter:**	5
**Percentage Maximum Peak:**	0
**Maximum Peak Measure:**	Gel
**Edge Detection Method:**	Fixed Width
**Fixed Width pixels:**	17
**Band Positions Edited:**	Yes

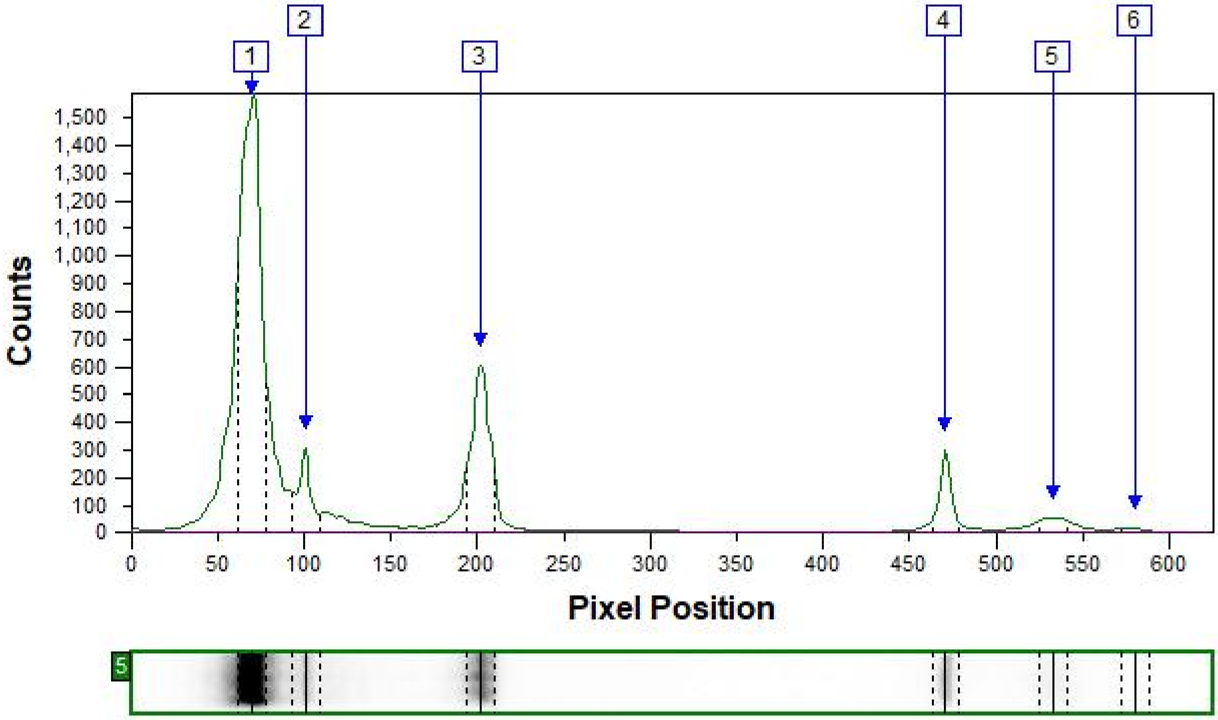

Band Table

Band No	Volume	Vol+BkGnd	Band %
1	744789.27	746978.25	61.846
2	95116.51	97305.49	7.898
3	243756.83	245945.81	20.241
4	84302.08	86491.06	7.000
5	28729.87	30918.85	2.386
6	7563.77	9752.76	0.628

Lane Data Report: rw 2025_10_23 p5 timecourse analysis-[phosphor].gel, Lane 6

Summary

**Name:**	Lane 6

Background

**Background Type:**	Image Rectangle
**Rectangle:**	Left 313, Right 1075, Top 987, Bottom 1077

Band Detection

**Automatic Detection:**	Yes
**Minimum Band Slope:**	182
**Median Filter:**	5
**Percentage Maximum Peak:**	0
**Maximum Peak Measure:**	Gel
**Edge Detection Method:**	Fixed Width
**Fixed Width pixels:**	17
**Band Positions Edited:**	Yes

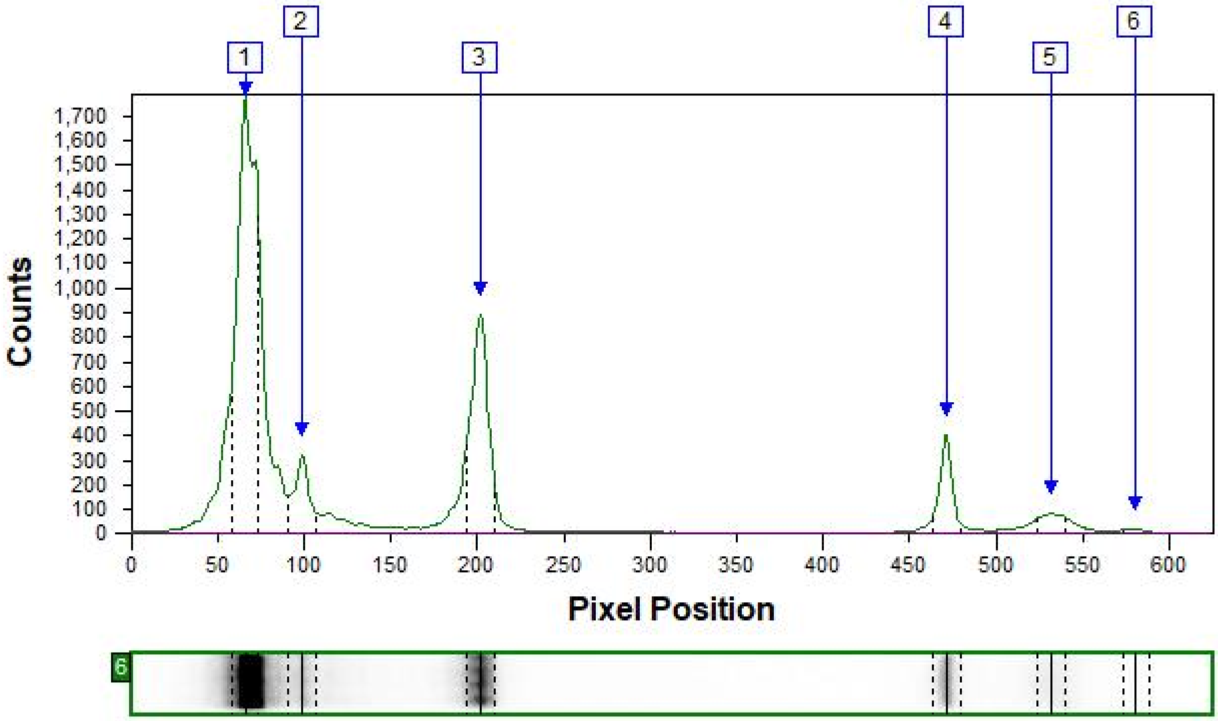

Band Table

Band No	Volume	Vol+BkGnd	Band %
1	768764.43	770953.41	54.817
2	112846.11	115035.09	8.047
3	350392.38	352581.36	24.985
4	119810.39	121999.37	8.543
5	42353.33	44542.31	3.020
6	8255.10	10444.08	0.589

ImageQuant TL

Lane Data Report: rw 2025_10_23 p5 timecourse analysis-[phosphor].gel, Lane 7

Summary

**Name:**	Lane 7

Background

**Background Type:**	Image Rectangle
**Rectangle:**	Left 313, Right 1075, Top 987, Bottom 1077

Band Detection

**Automatic Detection:**	Yes
**Minimum Band Slope:**	182
**Median Filter:**	5
**Percentage Maximum Peak:**	0
**Maximum Peak Measure:**	Gel
**Edge Detection Method:**	Fixed Width
**Fixed Width pixels:**	17
**Band Positions Edited:**	Yes

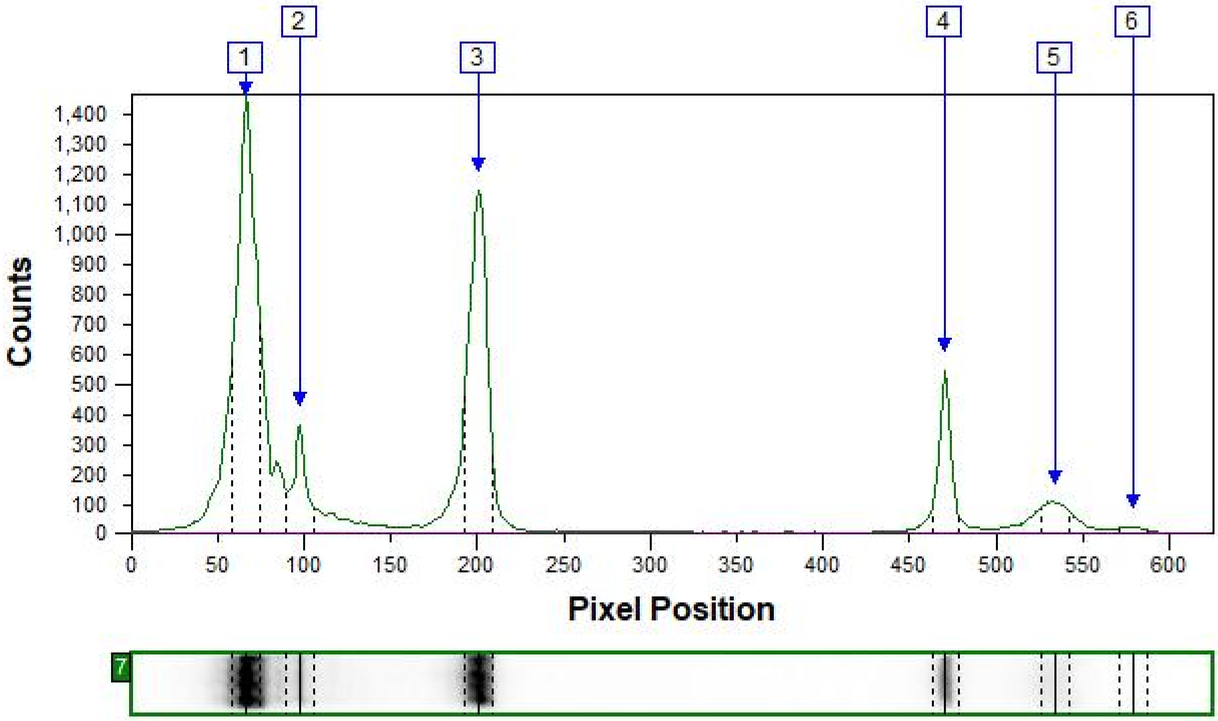

Band Table

Band No	Volume	Vol+BkGnd	Band %
1	610050.64	612239.62	43.013
2	111755.59	113944.57	7.879
3	473073.70	475262.69	33.355
4	157168.24	159357.22	11.081
5	55371.40	57560.38	3.904
6	10890.17	13079.15	0.768

ImageQuant TL

Lane Data Report: rw 2025_10_23 p5 timecourse analysis-[phosphor].gel, Lane 8

Summary

**Name:**	Lane 8

Background

**Background Type:**	Image Rectangle
**Rectangle:**	Left 313, Right 1075, Top 987, Bottom 1077

Band Detection

**Automatic Detection:**	Yes
**Minimum Band Slope:**	182
**Median Filter:**	5
**Percentage Maximum Peak:**	0
**Maximum Peak Measure:**	Gel
**Edge Detection Method:**	Fixed Width
**Fixed Width pixels:**	17
**Band Positions Edited:**	Yes

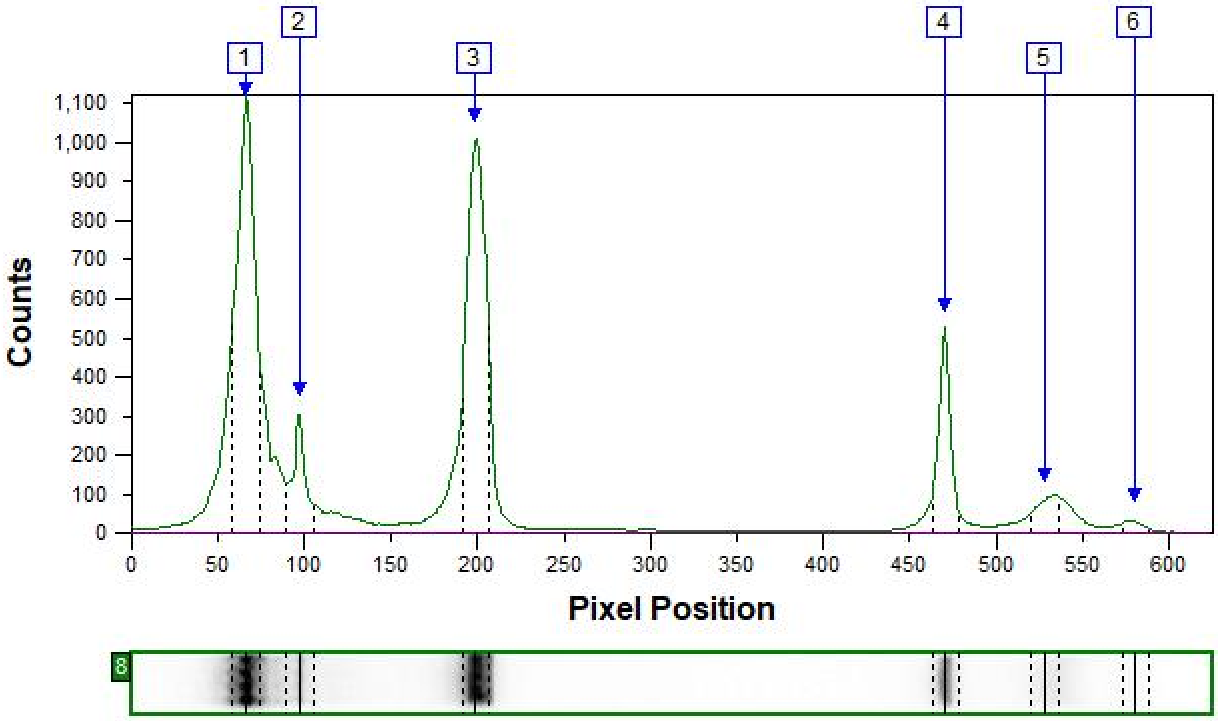

Band Table

Band No	Volume	Vol+BkGnd	Band %
1	465159.97	467348.95	38.429
2	92081.73	94270.71	7.607
3	442542.81	444731.79	36.561
4	152681.76	154870.74	12.614
5	44742.67	46931.65	3.696
6	13225.41	15414.39	1.093

ImageQuant TL

Lane Data Report: rw 2025_10_23 p5 timecourse analysis-[phosphor].gel, Lane 9

Summary

**Name:**	Lane 9

Background

**Background Type:**	Image Rectangle
**Rectangle:**	Left 313, Right 1075, Top 987, Bottom 1077

Band Detection

**Automatic Detection:**	Yes
**Minimum Band Slope:**	182
**Median Filter:**	5
**Percentage Maximum Peak:**	0
**Maximum Peak Measure:**	Gel
**Edge Detection Method:**	Fixed Width
**Fixed Width pixels:**	17
**Band Positions Edited:**	Yes

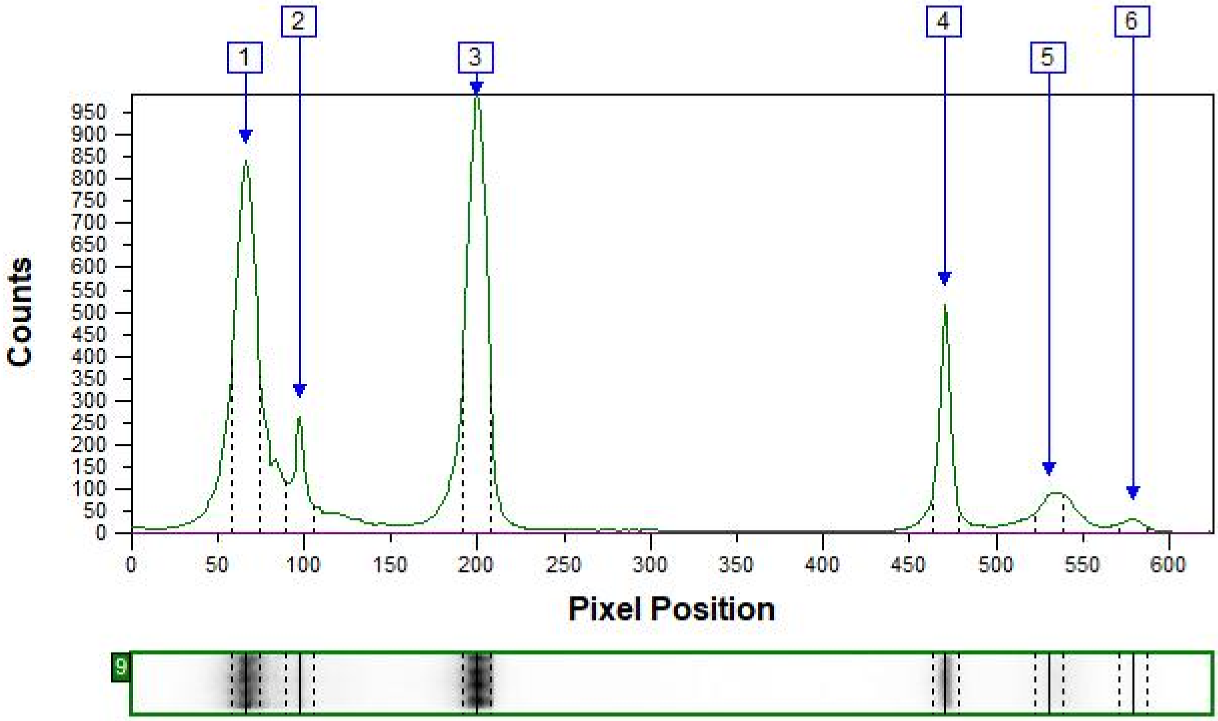

Band Table

Band No	Volume	Vol+BkGnd	Band %
1	374081.08	376270.06	34.209
2	81014.62	83203.60	7.409
3	435196.00	437384.99	39.798
4	145870.22	148059.20	13.340
5	43463.72	45652.71	3.975
6	13879.24	16068.23	1.269

ImageQuant TL

Lane Data Report: rw 2025_10_23 p5 timecourse analysis-[phosphor].gel, Lane 10

Summary

**Name:**	Lane 10

Background

**Background Type:**	Image Rectangle
**Rectangle:**	Left 313, Right 1075, Top 987, Bottom 1077

Band Detection

**Automatic Detection:**	Yes
**Minimum Band Slope:**	182
**Median Filter:**	5
**Percentage Maximum Peak:**	0
**Maximum Peak Measure:**	Gel
**Edge Detection Method:**	Fixed Width
**Fixed Width pixels:**	17
**Band Positions Edited:**	Yes

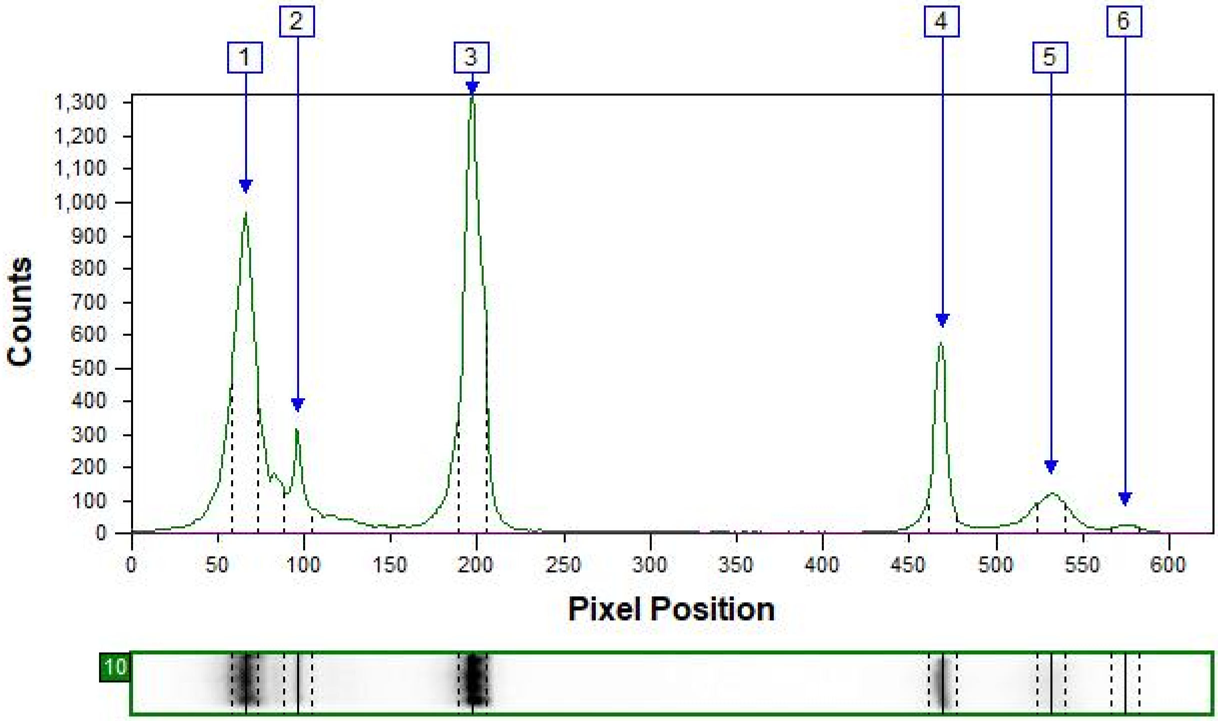

Band Table

Band No	Volume	Vol+BkGnd	Band %
1	413676.01	415864.99	32.436
2	93366.47	95555.45	7.321
3	517166.67	519355.65	40.551
4	177910.35	180099.34	13.950
5	60615.52	62804.50	4.753
6	12626.78	14815.77	0.990

Lane Data Report: rw 2025_10_23 p5 timecourse analysis-[phosphor].gel, Lane 11

Summary

**Name:**	Lane 11

Background

**Background Type:**	Image Rectangle
**Rectangle:**	Left 313, Right 1075, Top 987, Bottom 1077

Band Detection

**Automatic Detection:**	Yes
**Minimum Band Slope:**	182
**Median Filter:**	5
**Percentage Maximum Peak:**	0
**Maximum Peak Measure:**	Gel
**Edge Detection Method:**	Fixed Width
**Fixed Width pixels:**	17
**Band Positions Edited:**	Yes

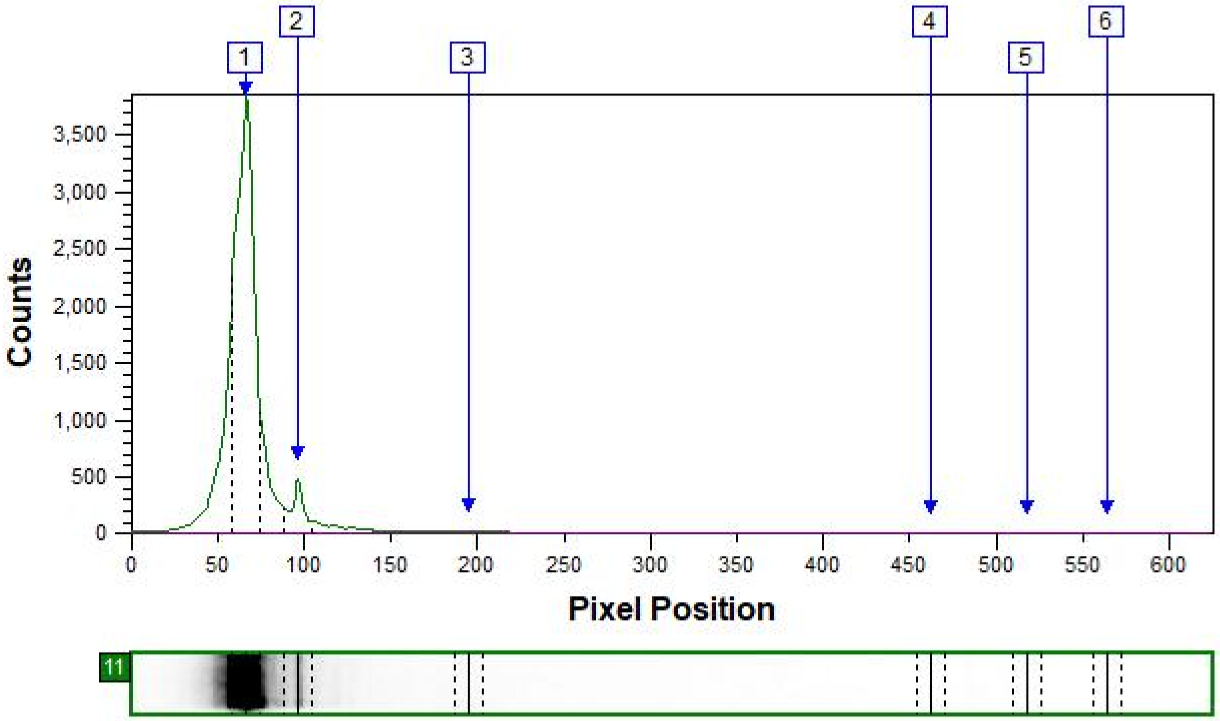

Band Table

Band No	Volume	Vol+BkGnd	Band %
1	1623336.86	1625525.84	90.895
2	144976.70	147165.69	8.118
3	14402.63	16591.62	0.806
4	1671.76	3860.74	0.094
5	1180.51	3369.49	0.066
6	387.25	2576.23	0.022

ImageQuant TL

Lane Data Report: rw 2025_10_23 p5 timecourse analysis-[phosphor].gel, Lane 12

Summary

**Name:**	Lane 12

Background

**Background Type:**	Image Rectangle
**Rectangle:**	Left 313, Right 1075, Top 987, Bottom 1077

Band Detection

**Automatic Detection:**	Yes
**Minimum Band Slope:**	182
**Median Filter:**	5
**Percentage Maximum Peak:**	0
**Maximum Peak Measure:**	Gel
**Edge Detection Method:**	Fixed Width
**Fixed Width pixels:**	17
**Band Positions Edited:**	Yes

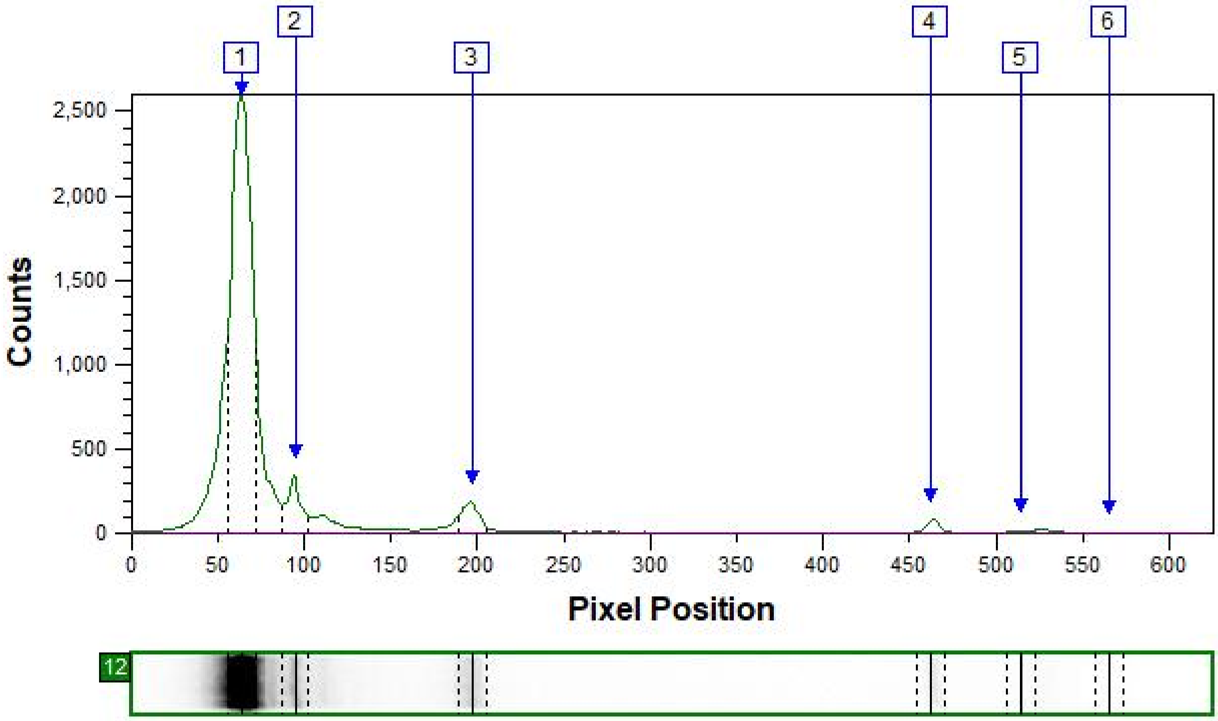

Band Table

Band No	Volume	Vol+BkGnd	Band %
1	1181006.79	1183195.78	83.216
2	119448.62	121637.60	8.417
3	81060.50	83249.48	5.712
4	27396.67	29585.66	1.930
5	8211.28	10400.27	0.579
6	2079.38	4268.37	0.147

ImageQuant TL

Lane Data Report: rw 2025_10_23 p5 timecourse analysis-[phosphor].gel, Lane 13

Summary

**Name:**	Lane 13

Background

**Background Type:**	Image Rectangle
**Rectangle:**	Left 313, Right 1075, Top 987, Bottom 1077

Band Detection

**Automatic Detection:**	Yes
**Minimum Band Slope:**	182
**Median Filter:**	5
**Percentage Maximum Peak:**	0
**Maximum Peak Measure:**	Gel
**Edge Detection Method:**	Fixed Width
**Fixed Width pixels:**	17
**Band Positions Edited:**	Yes

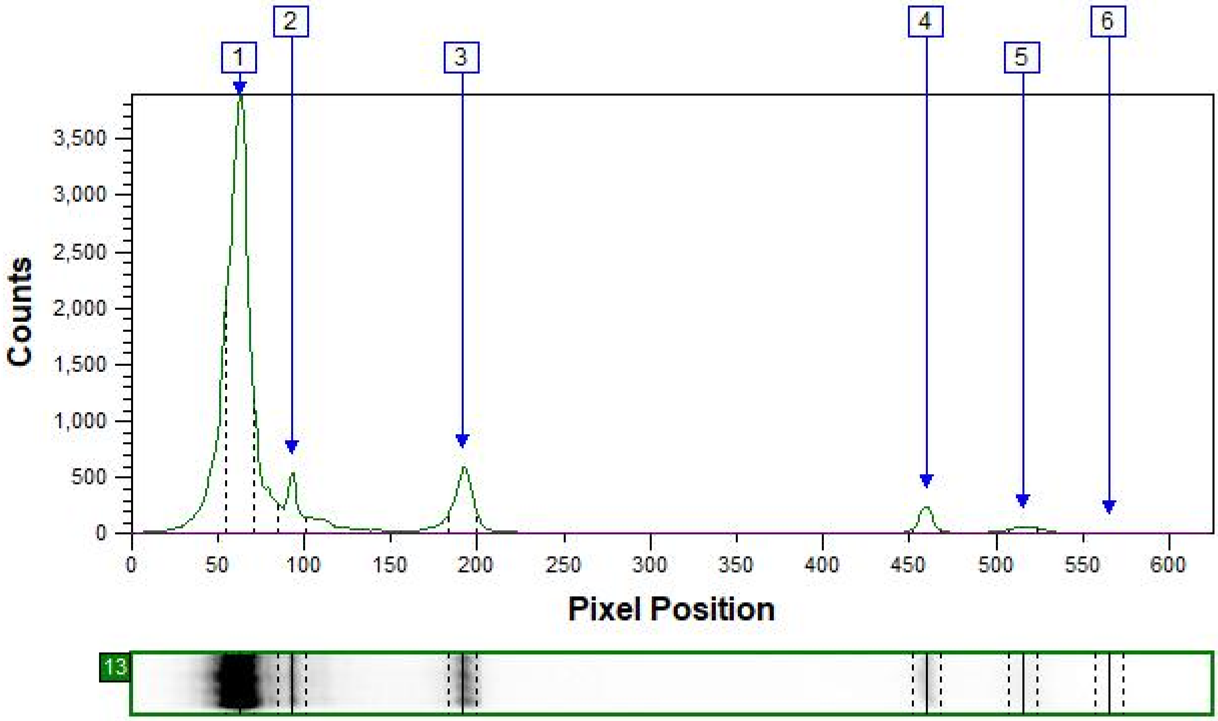

Band Table

Band No	Volume	Vol+BkGnd	Band %
1	1642603.56	1644792.54	76.032
2	172294.32	174483.30	7.975
3	231199.80	233388.78	10.702
4	78395.63	80584.61	3.629
5	33001.03	35190.01	1.528
6	2903.93	5092.92	0.134

ImageQuant TL

Lane Data Report: rw 2025_10_23 p5 timecourse analysis-[phosphor].gel, Lane 14

Summary

**Name:**	Lane 14

Background

**Background Type:**	Image Rectangle
**Rectangle:**	Left 313, Right 1075, Top 987, Bottom 1077

Band Detection

**Automatic Detection:**	Yes
**Minimum Band Slope:**	182
**Median Filter:**	5
**Percentage Maximum Peak:**	0
**Maximum Peak Measure:**	Gel
**Edge Detection Method:**	Fixed Width
**Fixed Width pixels:**	17
**Band Positions Edited:**	Yes

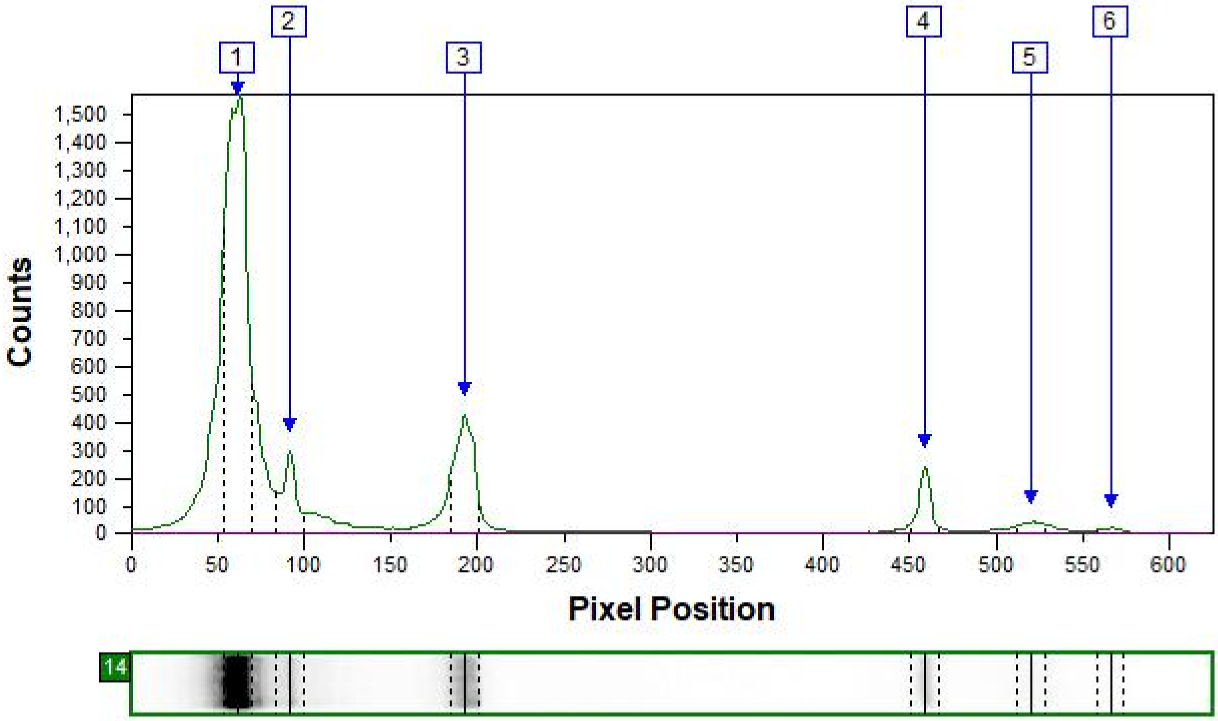

Band Table

Band No	Volume	Vol+BkGnd	Band %
1	756979.00	759167.99	66.647
2	97002.49	99191.47	8.540
3	186508.64	188697.62	16.421
4	66573.43	68762.42	5.861
5	19784.73	21973.71	1.742
6	8948.27	11137.25	0.788

ImageQuant TL

Lane Data Report: rw 2025_10_23 p5 timecourse analysis-[phosphor].gel, Lane 15

Summary

**Name:**	Lane 15

Background

**Background Type:**	Image Rectangle
**Rectangle:**	Left 313, Right 1075, Top 987, Bottom 1077

Band Detection

**Automatic Detection:**	Yes
**Minimum Band Slope:**	182
**Median Filter:**	5
**Percentage Maximum Peak:**	0
**Maximum Peak Measure:**	Gel
**Edge Detection Method:**	Fixed Width
**Fixed Width pixels:**	17
**Band Positions Edited:**	Yes

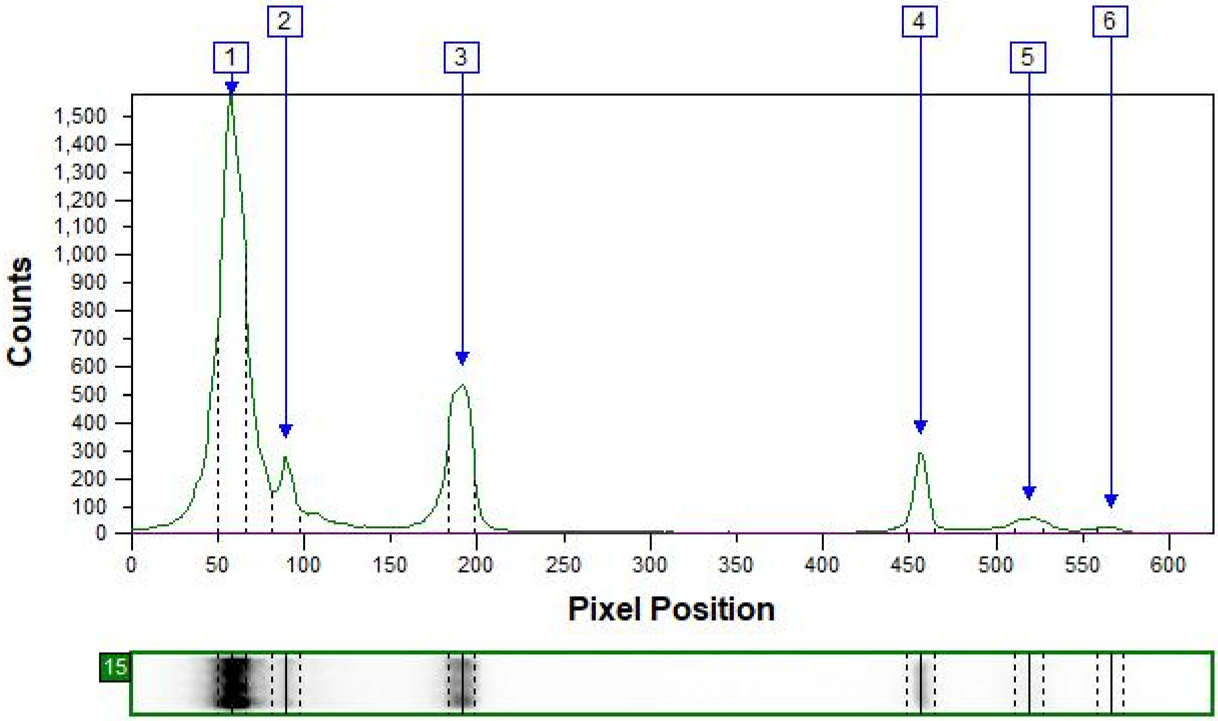

Band Table

Band No	Volume	Vol+BkGnd	Band %
1	719370.50	721559.48	58.644
2	106551.05	108740.04	8.686
3	263154.77	265343.75	21.453
4	97226.59	99415.57	7.926
5	29281.51	31470.49	2.387
6	11094.25	13283.23	0.904

Lane Data Report: rw 2025_10_23 p5 timecourse analysis-[phosphor].gel, Lane 16

Summary

**Name:**	Lane 16

Background

**Background Type:**	Image Rectangle
**Rectangle:**	Left 313, Right 1075, Top 987, Bottom 1077

Band Detection

**Automatic Detection:**	Yes
**Minimum Band Slope:**	182
**Median Filter:**	5
**Percentage Maximum Peak:**	0
**Maximum Peak Measure:**	Gel
**Edge Detection Method:**	Fixed Width
**Fixed Width pixels:**	17
**Band Positions Edited:**	Yes

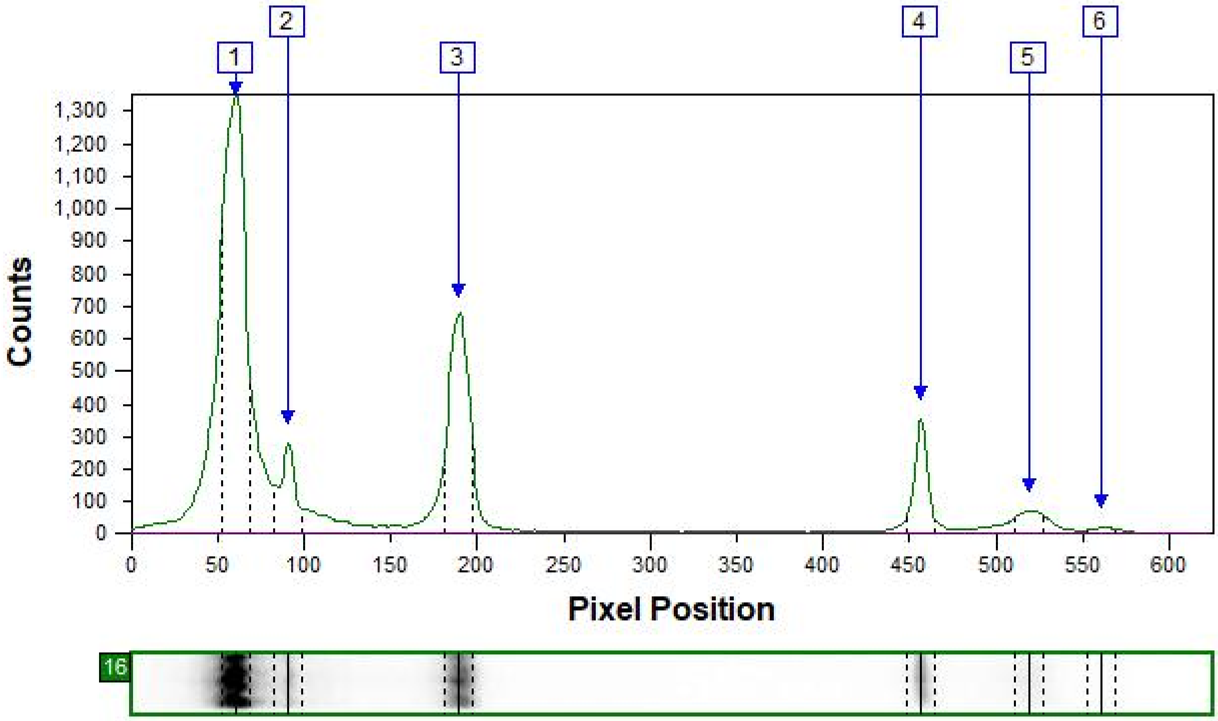

Band Table

Band No	Volume	Vol+BkGnd	Band %
1	641151.77	643340.75	53.751
2	100380.17	102569.15	8.415
3	299454.21	301643.20	25.105
4	108033.91	110222.90	9.057
5	36123.86	38312.84	3.028
6	7663.98	9852.97	0.643

ImageQuant TL

Lane Data Report: rw 2025_10_23 p5 timecourse analysis-[phosphor].gel, Lane 17

Summary

**Name:**	Lane 17

Background

**Background Type:**	Image Rectangle
**Rectangle:**	Left 313, Right 1075, Top 987, Bottom 1077

Band Detection

**Automatic Detection:**	Yes
**Minimum Band Slope:**	182
**Median Filter:**	5
**Percentage Maximum Peak:**	0
**Maximum Peak Measure:**	Gel
**Edge Detection Method:**	Fixed Width
**Fixed Width pixels:**	17
**Band Positions Edited:**	Yes

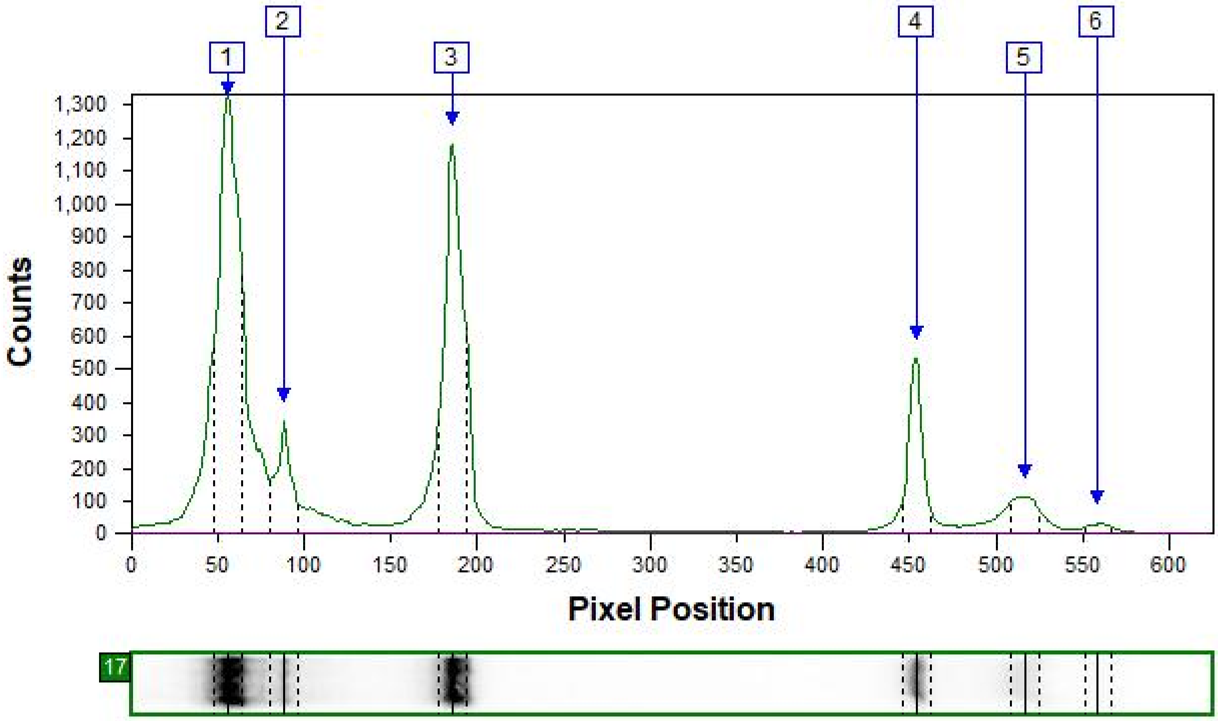

Band Table

Band No	Volume	Vol+BkGnd	Band %
1	589927.70	592116.68	41.569
2	117631.04	119820.03	8.289
3	464849.97	467038.95	32.755
4	173991.64	176180.63	12.260
5	58986.50	61175.48	4.156
6	13769.47	15958.45	0.970

IQTL Report version 8.1
Time of Report: 5:13 PM 10/23/2025 (Eastern Daylight Time)

ImageQuant TL

Lane Data Report: rw 2025_10_23 p5 timecourse analysis-[phosphor].gel, Lane 18

Summary

**Name:**	Lane 18

Background

**Background Type:**	Image Rectangle
**Rectangle:**	Left 313, Right 1075, Top 987, Bottom 1077

Band Detection

**Automatic Detection:**	Yes
**Minimum Band Slope:**	182
**Median Filter:**	5
**Percentage Maximum Peak:**	0
**Maximum Peak Measure:**	Gel
**Edge Detection Method:**	Fixed Width
**Fixed Width pixels:**	17
**Band Positions Edited:**	Yes

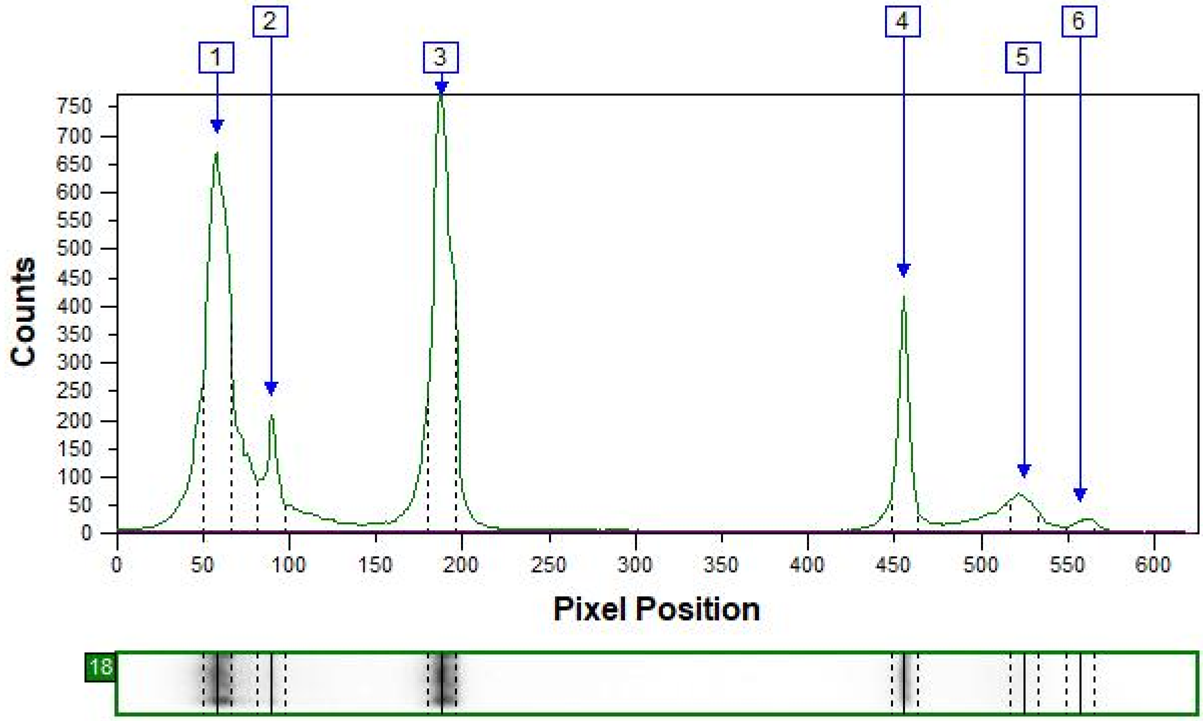

Band Table

Band No	Volume	Vol+BkGnd	Band %
1	302562.28	304751.26	35.902
2	66911.77	69100.75	7.940
3	316412.00	318600.99	37.546
4	113933.72	116122.70	13.519
5	32881.99	35070.97	3.902
6	10034.92	12223.91	1.191

Lane Data Report: rw 2025_10_23 p5 timecourse analysis-[phosphor].gel, Lane 19

Summary

**Name:**	Lane 19

Background

**Background Type:**	Image Rectangle
**Rectangle:**	Left 313, Right 1075, Top 987, Bottom 1077

Band Detection

**Automatic Detection:**	Yes
**Minimum Band Slope:**	182
**Median Filter:**	5
**Percentage Maximum Peak:**	0
**Maximum Peak Measure:**	Gel
**Edge Detection Method:**	Fixed Width
**Fixed Width pixels:**	17
**Band Positions Edited:**	Yes

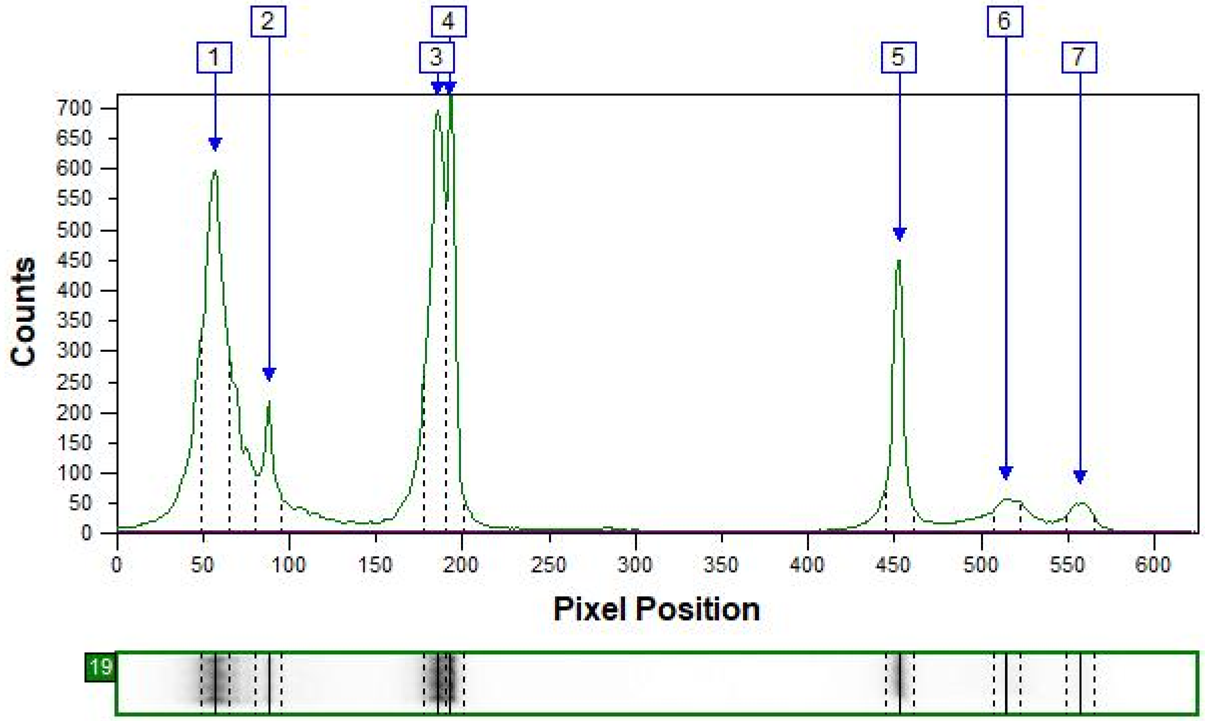

Band Table

Band No	Volume	Vol+BkGnd	Band %
1	261899.08	264088.06	29.275
2	68516.48	70705.47	7.659
3	223608.58	225250.32	24.995
4	164485.40	165990.32	18.386
5	124683.69	126872.67	13.937
6	27916.42	30105.41	3.121
7	23496.77	25685.75	2.626

ImageQuant TL

Lane Data Report: rw 2025_10_23 p5 timecourse analysis-[phosphor].gel, Lane 20

Summary

**Name:**	Lane 20

Background

**Background Type:**	Image Rectangle
**Rectangle:**	Left 313, Right 1075, Top 987, Bottom 1077

Band Detection

**Automatic Detection:**	Yes
**Minimum Band Slope:**	182
**Median Filter:**	5
**Percentage Maximum Peak:**	0
**Maximum Peak Measure:**	Gel
**Edge Detection Method:**	Fixed Width
**Fixed Width pixels:**	17
**Band Positions Edited:**	Yes

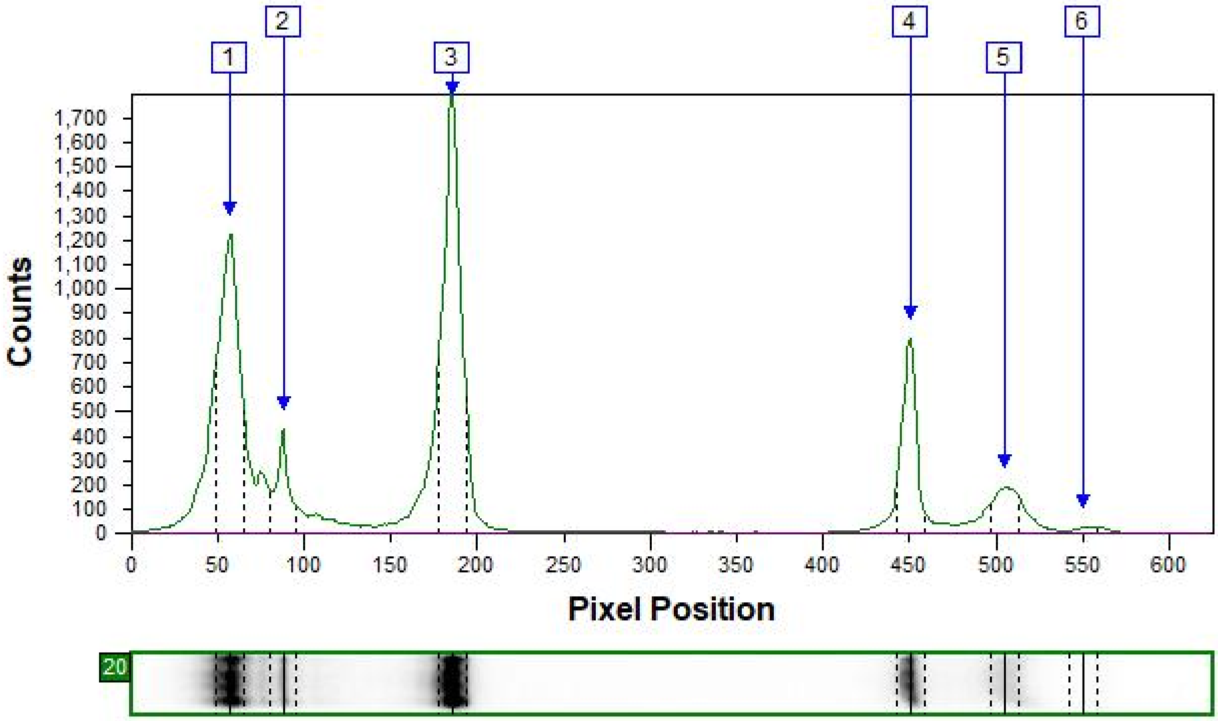

Band Table

Band No	Volume	Vol+BkGnd	Band %
1	541958.65	544147.63	31.122
2	131115.27	133304.26	7.529
3	691833.63	694022.61	39.729
4	268367.58	270556.56	15.411
5	96062.94	98251.92	5.516
6	12045.32	14234.30	0.692

ImageQuant TL

Lane Data Report: rw 2025_10_23 p5 timecourse analysis-[phosphor].gel, Lane 21

Summary

**Name:**	Lane 21

Background

**Background Type:**	Image Rectangle
**Rectangle:**	Left 313, Right 1075, Top 987, Bottom 1077

Band Detection

**Automatic Detection:**	Yes
**Minimum Band Slope:**	182
**Median Filter:**	5
**Percentage Maximum Peak:**	0
**Maximum Peak Measure:**	Gel
**Edge Detection Method:**	Fixed Width
**Fixed Width pixels:**	17
**Band Positions Edited:**	Yes

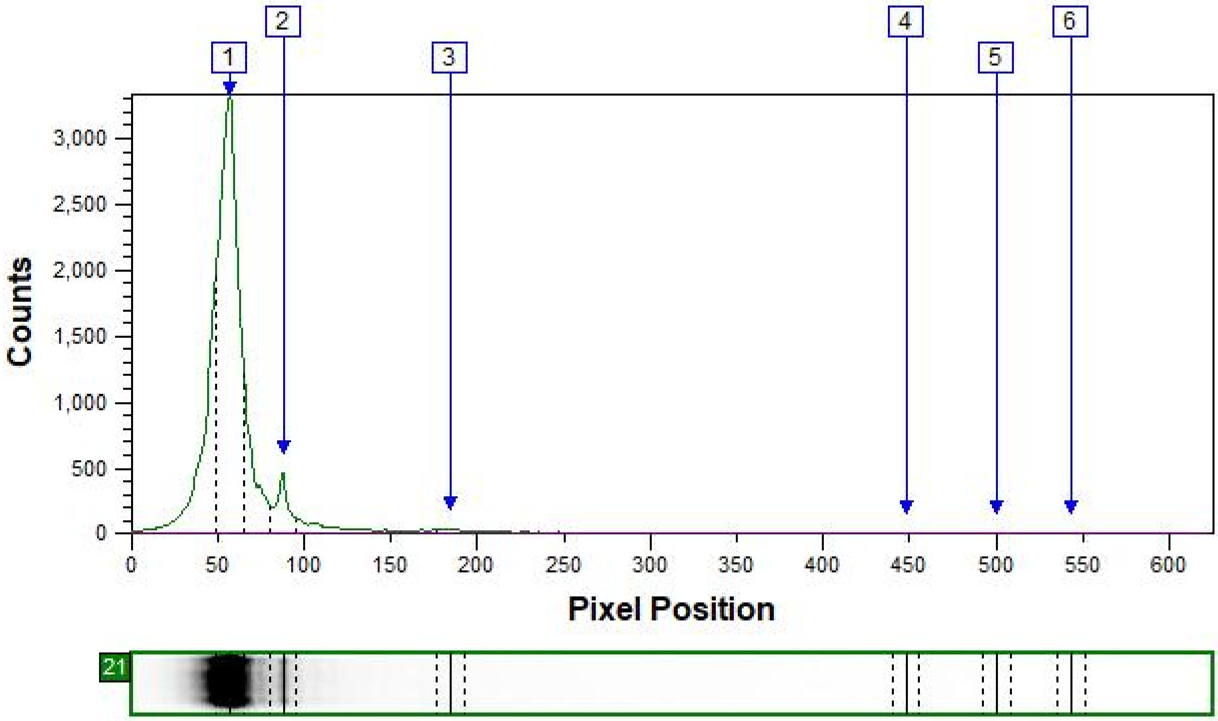

Band Table

Band No	Volume	Vol+BkGnd	Band %
1	1461811.96	1464000.94	89.838
2	142894.53	145083.51	8.782
3	16531.87	18720.85	1.016
4	3317.58	5506.56	0.204
5	2041.30	4230.28	0.125
6	575.71	2764.69	0.035

ImageQuant TL

Lane Data Report: rw 2025_10_23 p5 timecourse analysis-[phosphor].gel, Lane 22

Summary

**Name:**	Lane 22

Background

**Background Type:**	Image Rectangle
**Rectangle:**	Left 313, Right 1075, Top 987, Bottom 1077

Band Detection

**Automatic Detection:**	Yes
**Minimum Band Slope:**	182
**Median Filter:**	5
**Percentage Maximum Peak:**	0
**Maximum Peak Measure:**	Gel
**Edge Detection Method:**	Fixed Width
**Fixed Width pixels:**	17
**Band Positions Edited:**	Yes

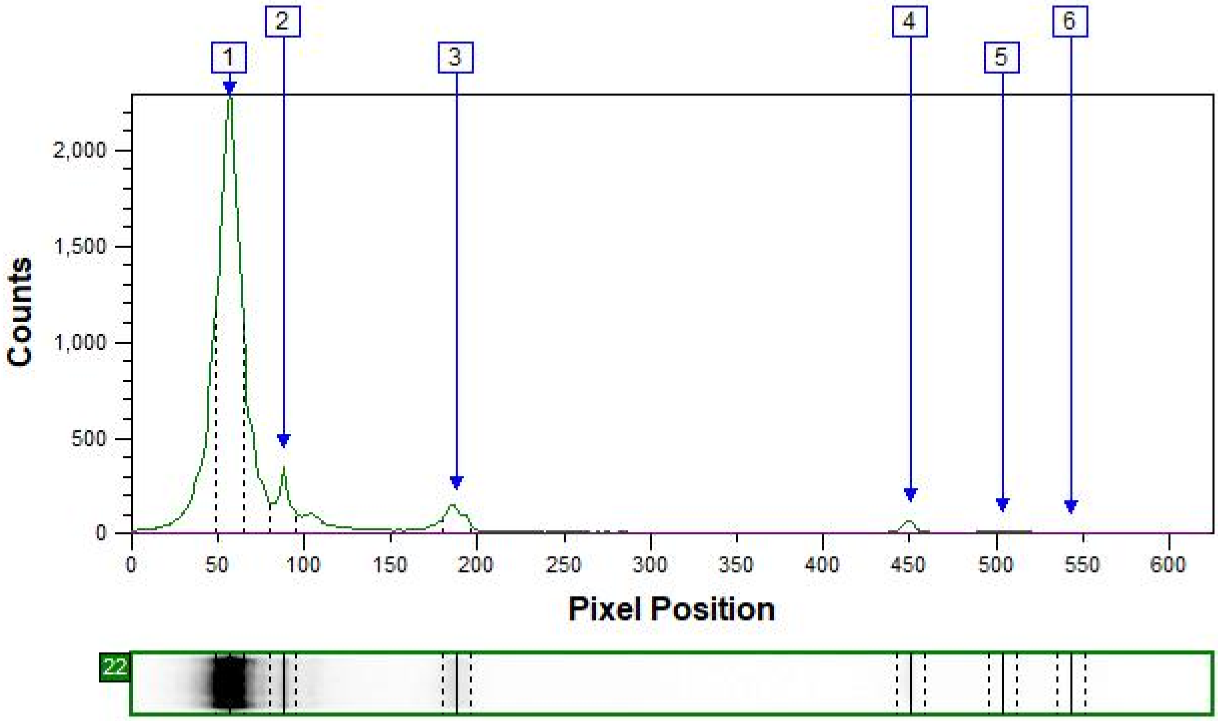

Band Table

Band No	Volume	Vol+BkGnd	Band %
1	1008110.23	1010299.21	83.032
2	112823.24	115012.22	9.293
3	63754.10	65943.08	5.251
4	20870.13	23059.11	1.719
5	7210.79	9399.77	0.594
6	1357.93	3546.92	0.112

ImageQuant TL

Lane Data Report: rw 2025_10_23 p5 timecourse analysis-[phosphor].gel, Lane 23

Summary

**Name:**	Lane 23

Background

**Background Type:**	Image Rectangle
**Rectangle:**	Left 313, Right 1075, Top 987, Bottom 1077

Band Detection

**Automatic Detection:**	Yes
**Minimum Band Slope:**	182
**Median Filter:**	5
**Percentage Maximum Peak:**	0
**Maximum Peak Measure:**	Gel
**Edge Detection Method:**	Fixed Width
**Fixed Width pixels:**	17
**Band Positions Edited:**	Yes

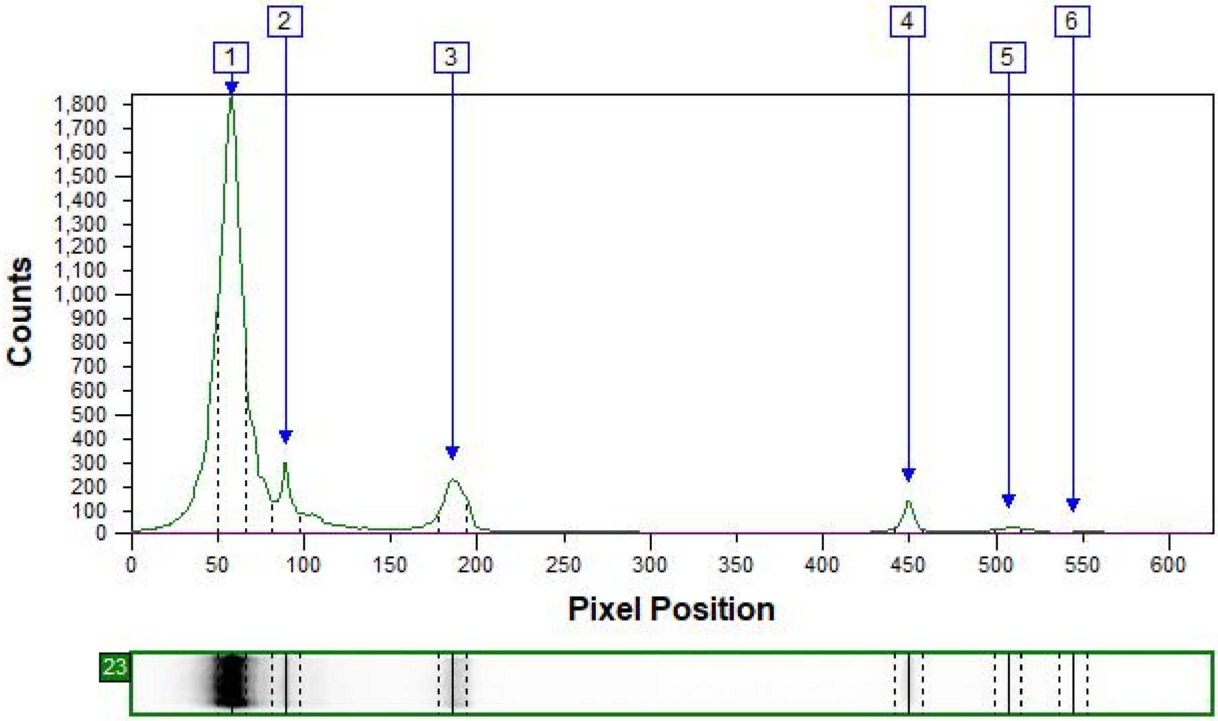

Band Table

Band No	Volume	Vol+BkGnd	Band %
1	802707.33	804896.31	76.205
2	94526.83	96715.81	8.974
3	102136.26	104325.24	9.696
4	39053.59	41242.57	3.708
5	11847.58	14036.56	1.125
6	3081.94	5270.93	0.293

ImageQuant TL

Lane Data Report: rw 2025_10_23 p5 timecourse analysis-[phosphor].gel, Lane 24

Summary

**Name:**	Lane 24

Background

**Background Type:**	Image Rectangle
**Rectangle:**	Left 313, Right 1075, Top 987, Bottom 1077

Band Detection

**Automatic Detection:**	Yes
**Minimum Band Slope:**	182
**Median Filter:**	5
**Percentage Maximum Peak:**	0
**Maximum Peak Measure:**	Gel
**Edge Detection Method:**	Fixed Width
**Fixed Width pixels:**	17
**Band Positions Edited:**	Yes

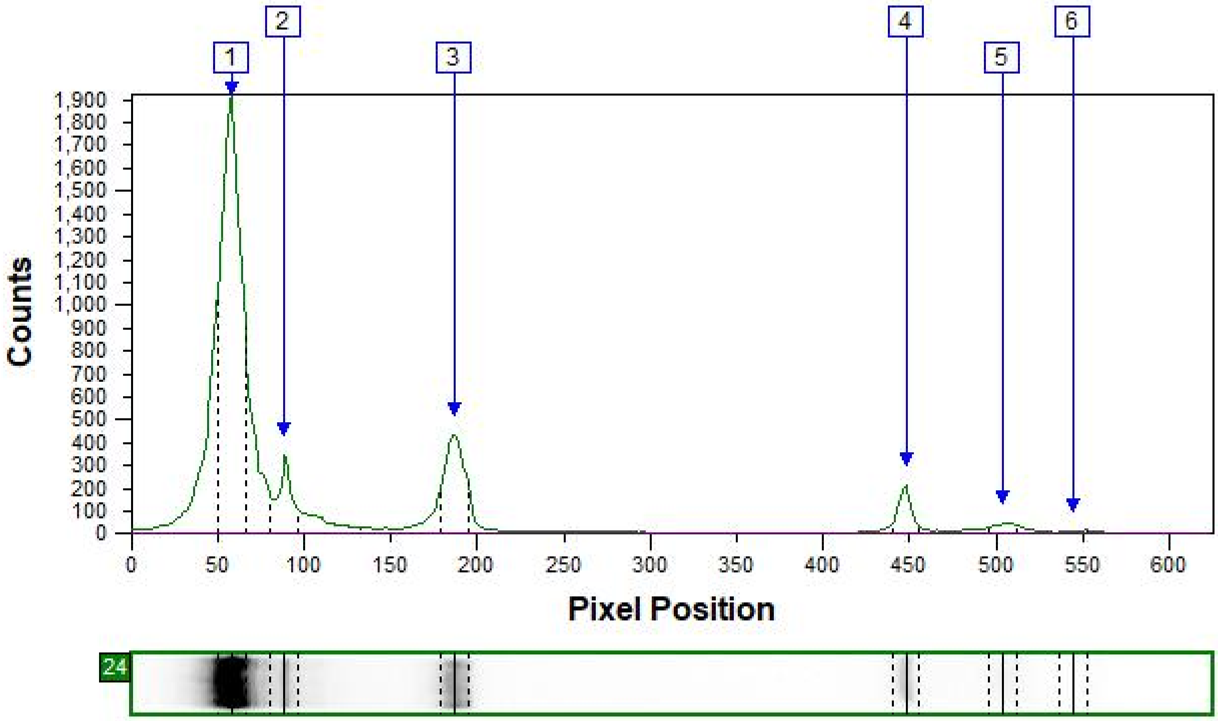

Band Table

Band No	Volume	Vol+BkGnd	Band %
1	847589.64	849778.62	67.983
2	112202.70	114391.68	9.000
3	193293.91	195482.89	15.504
4	66537.39	68726.37	5.337
5	21700.73	23889.71	1.741
6	5436.35	7625.33	0.436

ImageQuant TL

Lane Data Report: rw 2025_10_23 p5 timecourse analysis-[phosphor].gel, Lane 25

Summary

**Name:**	Lane 25

Background

**Background Type:**	Image Rectangle
**Rectangle:**	Left 313, Right 1075, Top 987, Bottom 1077

Band Detection

**Automatic Detection:**	Yes
**Minimum Band Slope:**	182
**Median Filter:**	5
**Percentage Maximum Peak:**	0
**Maximum Peak Measure:**	Gel
**Edge Detection Method:**	Fixed Width
**Fixed Width pixels:**	17
**Band Positions Edited:**	Yes

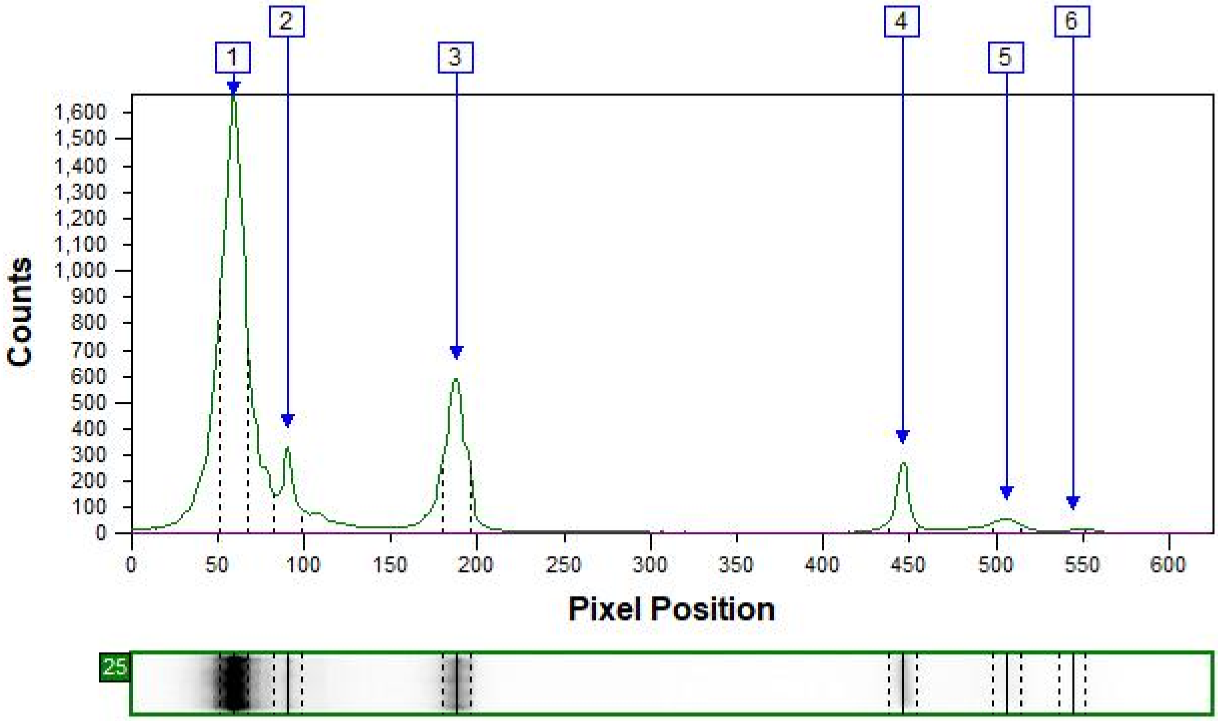

Band Table

Band No	Volume	Vol+BkGnd	Band %
1	742113.74	744302.72	61.116
2	106639.91	108828.89	8.782
3	246297.03	248486.01	20.284
4	84452.97	86641.95	6.955
5	27294.37	29483.35	2.248
6	7463.61	9652.59	0.615

ImageQuant TL

Lane Data Report: rw 2025_10_23 p5 timecourse analysis-[phosphor].gel, Lane 26

Summary

**Name:**	Lane 26

Background

**Background Type:**	Image Rectangle
**Rectangle:**	Left 313, Right 1075, Top 987, Bottom 1077

Band Detection

**Automatic Detection:**	Yes
**Minimum Band Slope:**	182
**Median Filter:**	5
**Percentage Maximum Peak:**	0
**Maximum Peak Measure:**	Gel
**Edge Detection Method:**	Fixed Width
**Fixed Width pixels:**	17
**Band Positions Edited:**	Yes

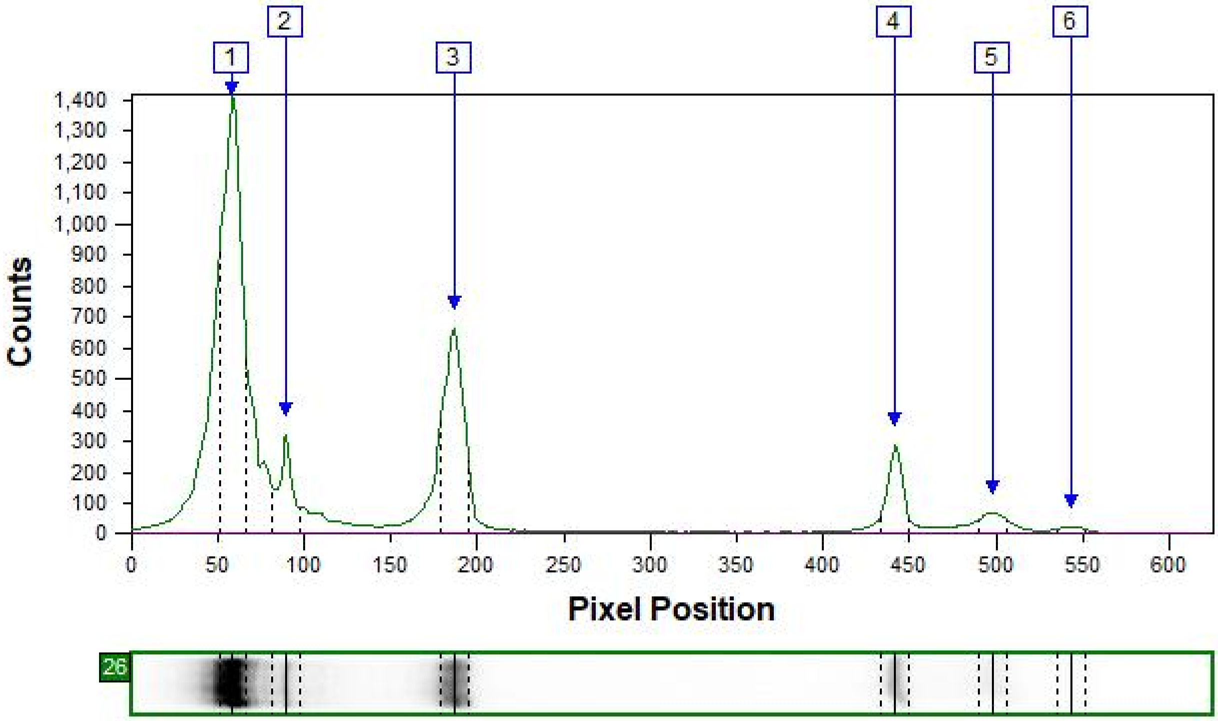

Band Table

Band No	Volume	Vol+BkGnd	Band %
1	632859.63	635048.61	54.261
2	101876.96	104065.94	8.735
3	288336.88	290525.87	24.722
4	99407.91	101596.89	8.523
5	33256.42	35445.40	2.851
6	10576.79	12765.77	0.907

ImageQuant TL

Lane Data Report: rw 2025_10_23 p5 timecourse analysis-[phosphor].gel, Lane 27

Summary

**Name:**	Lane 27

Background

**Background Type:**	Image Rectangle
**Rectangle:**	Left 313, Right 1075, Top 987, Bottom 1077

Band Detection

**Automatic Detection:**	Yes
**Minimum Band Slope:**	182
**Median Filter:**	5
**Percentage Maximum Peak:**	0
**Maximum Peak Measure:**	Gel
**Edge Detection Method:**	Fixed Width
**Fixed Width pixels:**	17
**Band Positions Edited:**	Yes

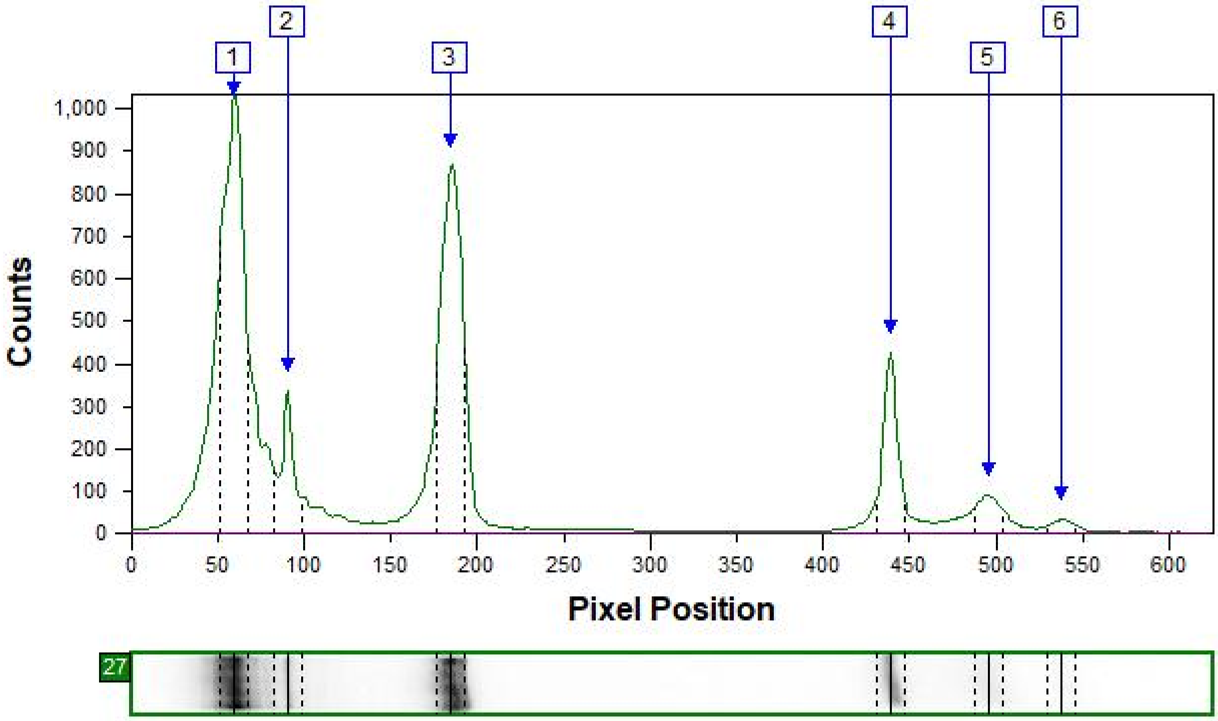

Band Table

Band No	Volume	Vol+BkGnd	Band %
1	472212.29	474401.27	40.286
2	101200.89	103389.87	8.634
3	400648.39	402837.37	34.181
4	140150.91	142339.89	11.957
5	43374.84	45563.83	3.700
6	14564.46	16753.44	1.243

ImageQuant TL

Lane Data Report: rw 2025_10_23 p5 timecourse analysis-[phosphor].gel, Lane 28

Summary

**Name:**	Lane 28

Background

**Background Type:**	Image Rectangle
**Rectangle:**	Left 313, Right 1075, Top 987, Bottom 1077

Band Detection

**Automatic Detection:**	Yes
**Minimum Band Slope:**	182
**Median Filter:**	5
**Percentage Maximum Peak:**	0
**Maximum Peak Measure:**	Gel
**Edge Detection Method:**	Fixed Width
**Fixed Width pixels:**	17
**Band Positions Edited:**	Yes

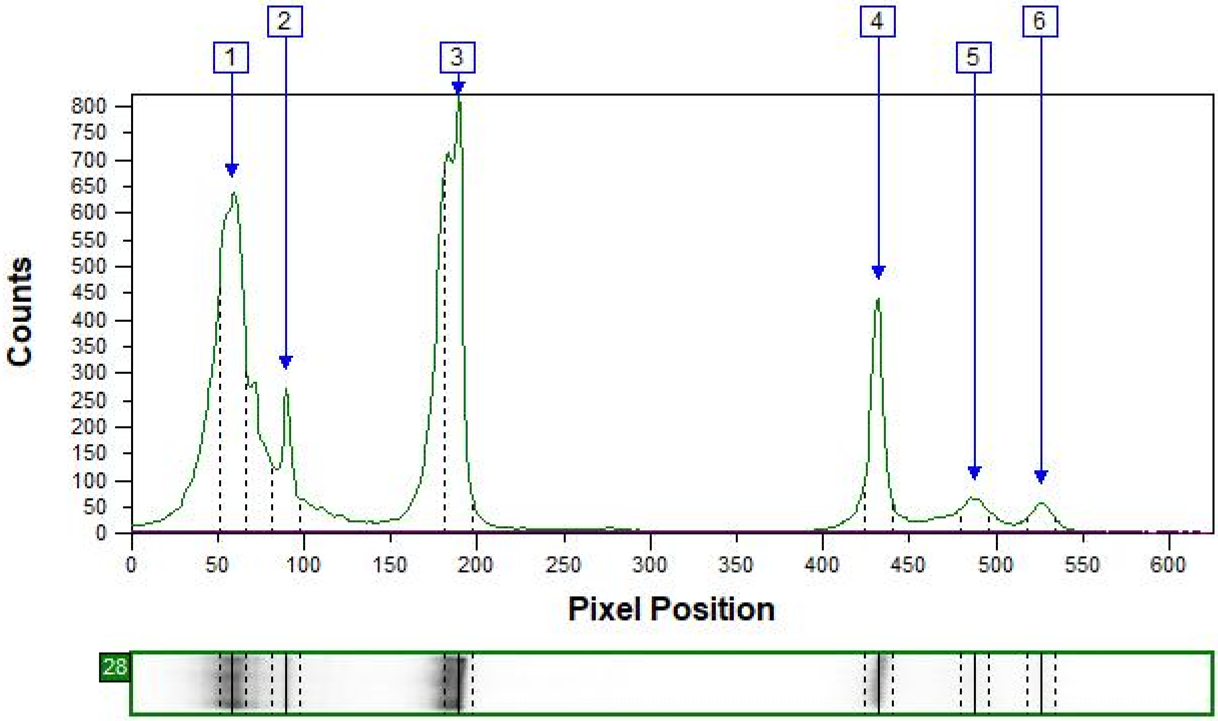

Band Table

Band No	Volume	Vol+BkGnd	Band %
1	317602.64	319792.00	34.520
2	84122.99	86312.68	9.143
3	322143.64	324332.69	35.014
4	139270.35	141464.98	15.137
5	32159.61	34350.12	3.495
6	24744.49	26935.75	2.689

ImageQuant TL

Lane Data Report: rw 2025_10_23 p5 timecourse analysis-[phosphor].gel, Lane 29

Summary

**Name:**	Lane 29

Background

**Background Type:**	Image Rectangle
**Rectangle:**	Left 313, Right 1075, Top 987, Bottom 1077

Band Detection

**Automatic Detection:**	Yes
**Minimum Band Slope:**	182
**Median Filter:**	5
**Percentage Maximum Peak:**	0
**Maximum Peak Measure:**	Gel
**Edge Detection Method:**	Fixed Width
**Fixed Width pixels:**	17
**Band Positions Edited:**	Yes

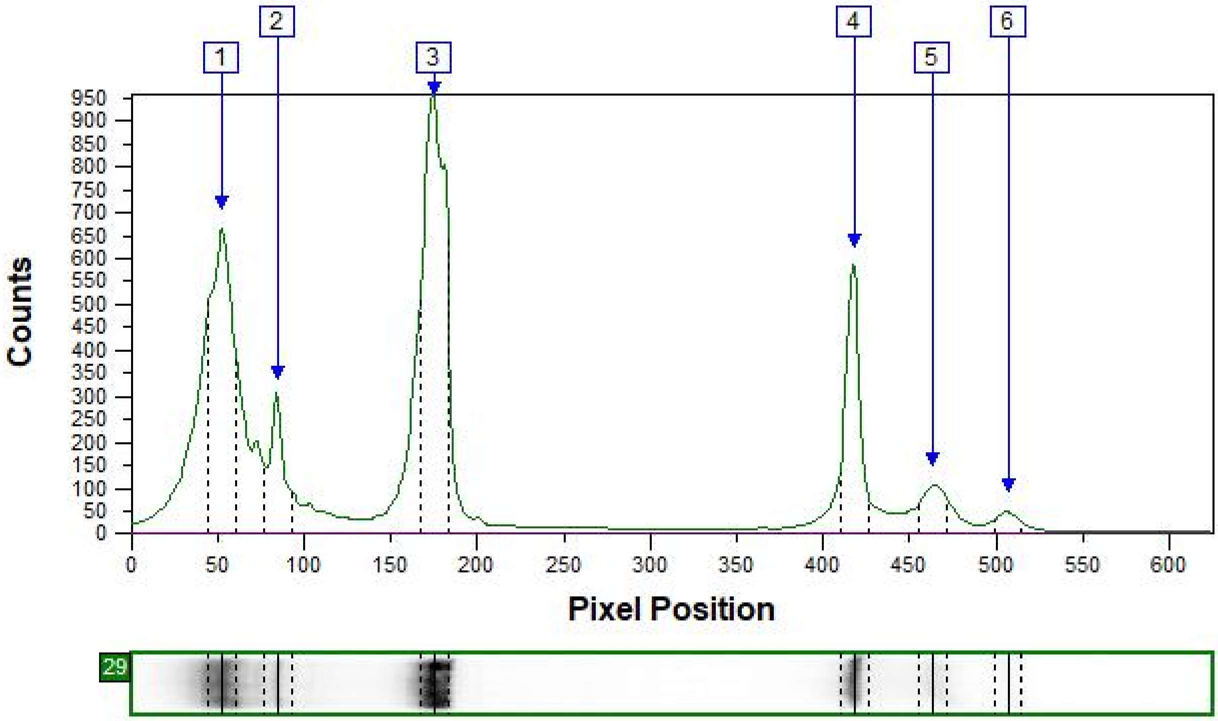

Band Table

Band No	Volume	Vol+BkGnd	Band %
1	319298.73	321489.04	27.813
2	100395.46	102586.23	8.745
3	461459.68	463650.98	40.197
4	193193.27	195387.11	16.829
5	52197.36	54387.75	4.547
6	21461.93	23652.70	1.869

ImageQuant TL

Lane Data Report: rw 2025_10_23 p5 timecourse analysis-[phosphor].gel, Lane 30

Summary

**Name:**	Lane 30

Background

**Background Type:**	Image Rectangle
**Rectangle:**	Left 313, Right 1075, Top 987, Bottom 1077

Band Detection

**Automatic Detection:**	Yes
**Minimum Band Slope:**	182
**Median Filter:**	5
**Percentage Maximum Peak:**	0
**Maximum Peak Measure:**	Gel
**Edge Detection Method:**	Fixed Width
**Fixed Width pixels:**	17
**Band Positions Edited:**	Yes

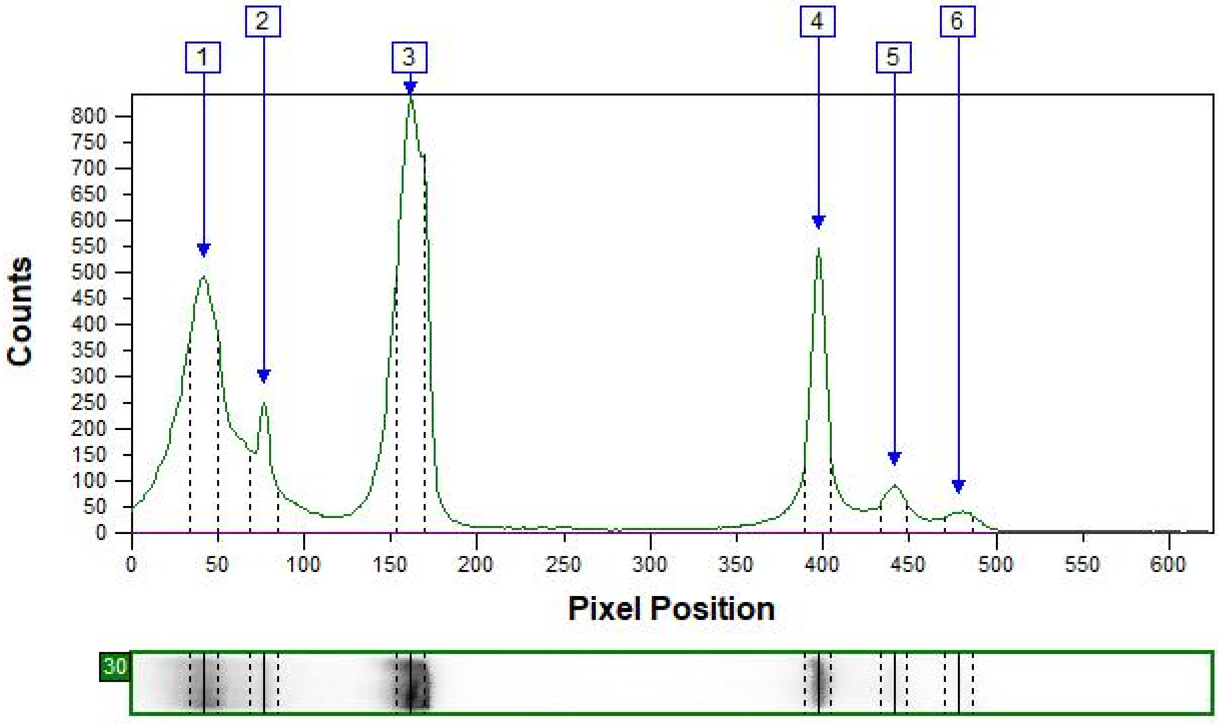

Band Table

Band No	Volume	Vol+BkGnd	Band %
1	256154.04	258358.46	24.940
2	97409.34	99599.89	9.484
3	412014.89	414204.77	40.115
4	198521.04	200709.79	19.329
5	42536.02	44726.17	4.141
6	20452.10	22642.74	1.991
