# Supplementary material for: PCanPIE: A group I intron platform for efficient circRNA synthesis at ambient temperatures
Source: RNA. Author manuscript; Available in PMC 2026 Nov 9. (PMC13580324; doi:10.1261/rna.081078.126)
Supplement: Supplementary file 1 [file NIHMS2209016-supplement-Data_source_files.zip › 14. Data source files/Fig. 3 Data/2025_10_23 P8_P6b timecourse/RW 2025_10_23 P8_P6b-[Phosphor]tif_AllLanes.pdf]

IQTL Report version 8.1
Time of Report: 5:06 PM 10/23/2025 (Eastern Daylight Time)

ImageQuant TL

Analysis Report: rw 2025_10_23 p8_p6b-[phosphor].tif

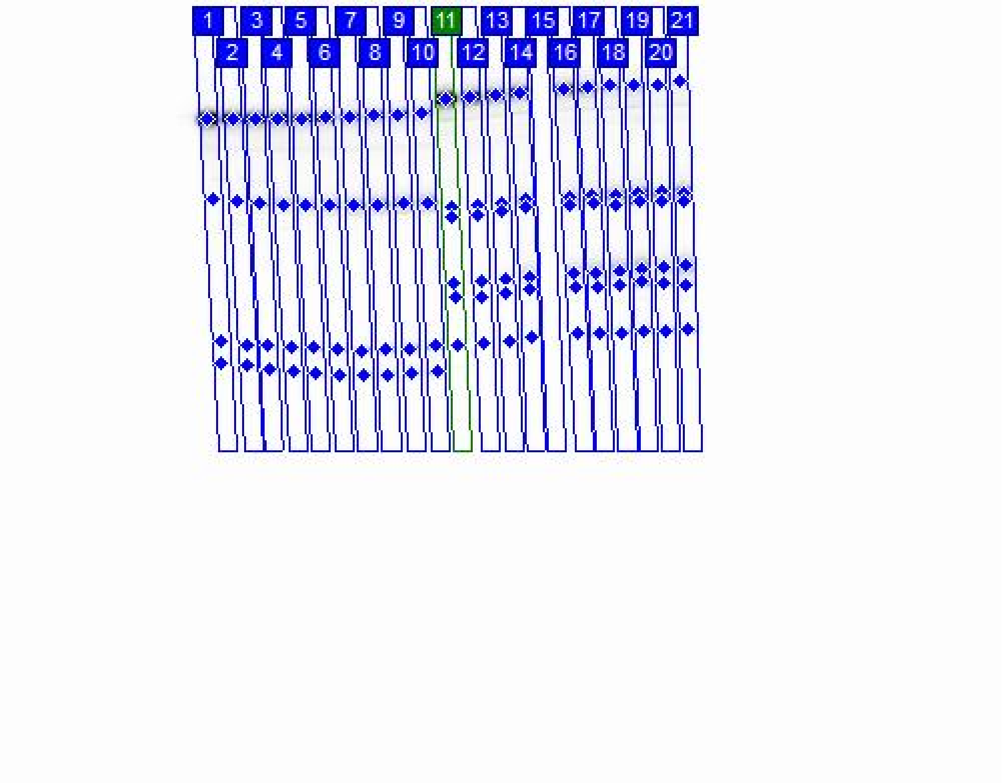

Lane Table

Lane	Bands	Band Volume	Lane Volume
Lane 1	4	10018189.00	12366817.00
Lane 2	4	6035817.92	7751871.46
Lane 3	4	6229894.00	7976554.00
Lane 4	4	6599453.00	8440261.00
Lane 5	4	5412259.00	6911481.00
Lane 6	4	5592563.00	7191705.00
Lane 7	4	5369673.00	7055942.00
Lane 8	4	4973796.00	6496301.00
Lane 9	4	4816227.00	6265698.00
Lane 10	4	4901954.00	6541062.00
Lane 11	6	6114355.00	8639781.00
Lane 12	6	3909951.00	5716013.00
Lane 13	6	3404872.00	4991511.00
Lane 14	6	4340306.00	6264912.00
Lane 15	0	0.00	129665.00
Lane 16	6	3787312.00	5683025.00
Lane 17	6	4179302.00	6183745.00
Lane 18	6	3583059.00	5432832.00
Lane 19	6	5138313.00	7409069.00
Lane 20	6	2973334.00	4258144.00
Lane 21	6	3477531.00	5248211.00

ImageQuant TL

Lane Data Report: rw 2025_10_23 p8_p6b-[phosphor].tif, Lane 1

Summary

**Name:**	Lane 1

Background

**Background Type:**	Image Rectangle
**Rectangle:**	Left 429, Right 1125, Top 691, Bottom 783

Band Detection

**Automatic Detection:**	Yes
**Minimum Band Slope:**	182
**Median Filter:**	5
**Percentage Maximum Peak:**	0
**Maximum Peak Measure:**	Gel
**Edge Detection Method:**	Fixed Width
**Fixed Width pixels:**	17
**Band Positions Edited:**	Yes

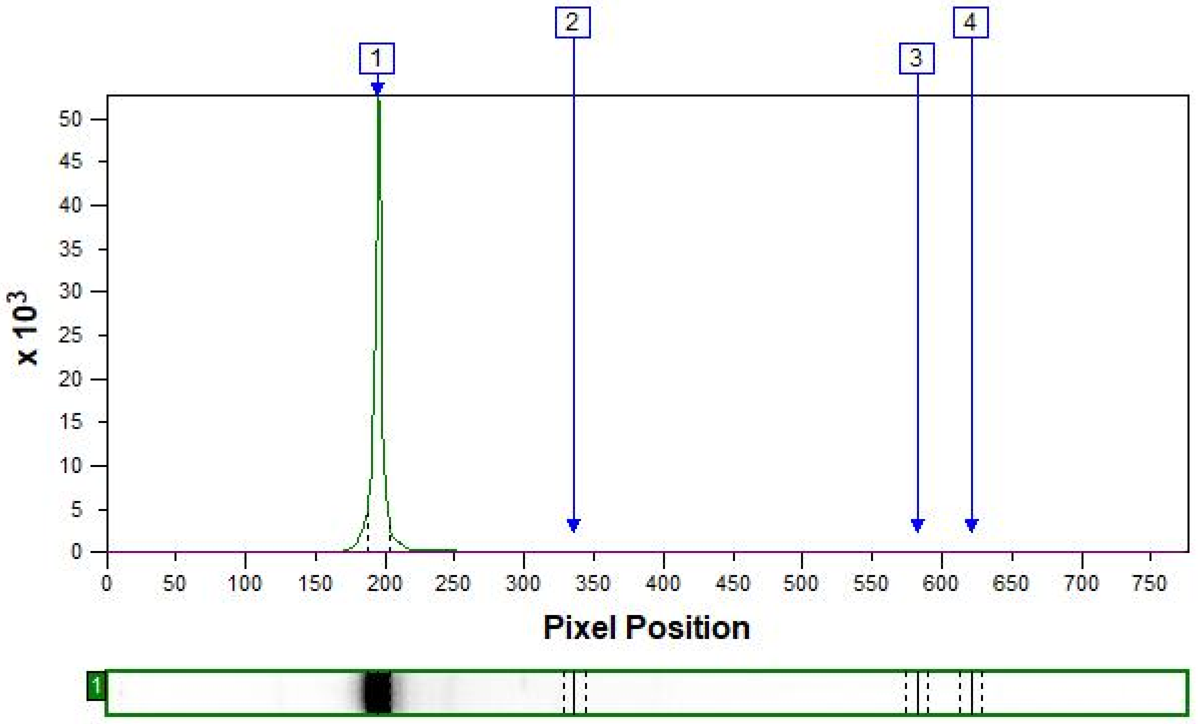

Band Table

Band No	Volume	Vol+BkGnd	Band %
1	9963001.00	9970681.00	99.449
2	40380.00	48060.00	0.403
3	3309.00	10989.00	0.033
4	11499.00	19179.00	0.115

ImageQuant TL

Lane Data Report: rw 2025_10_23 p8_p6b-[phosphor].tif, Lane 2

Summary

**Name:**	Lane 2

Background

**Background Type:**	Image Rectangle
**Rectangle:**	Left 429, Right 1125, Top 691, Bottom 783

Band Detection

**Automatic Detection:**	Yes
**Minimum Band Slope:**	182
**Median Filter:**	5
**Percentage Maximum Peak:**	0
**Maximum Peak Measure:**	Gel
**Edge Detection Method:**	Fixed Width
**Fixed Width pixels:**	17
**Band Positions Edited:**	Yes

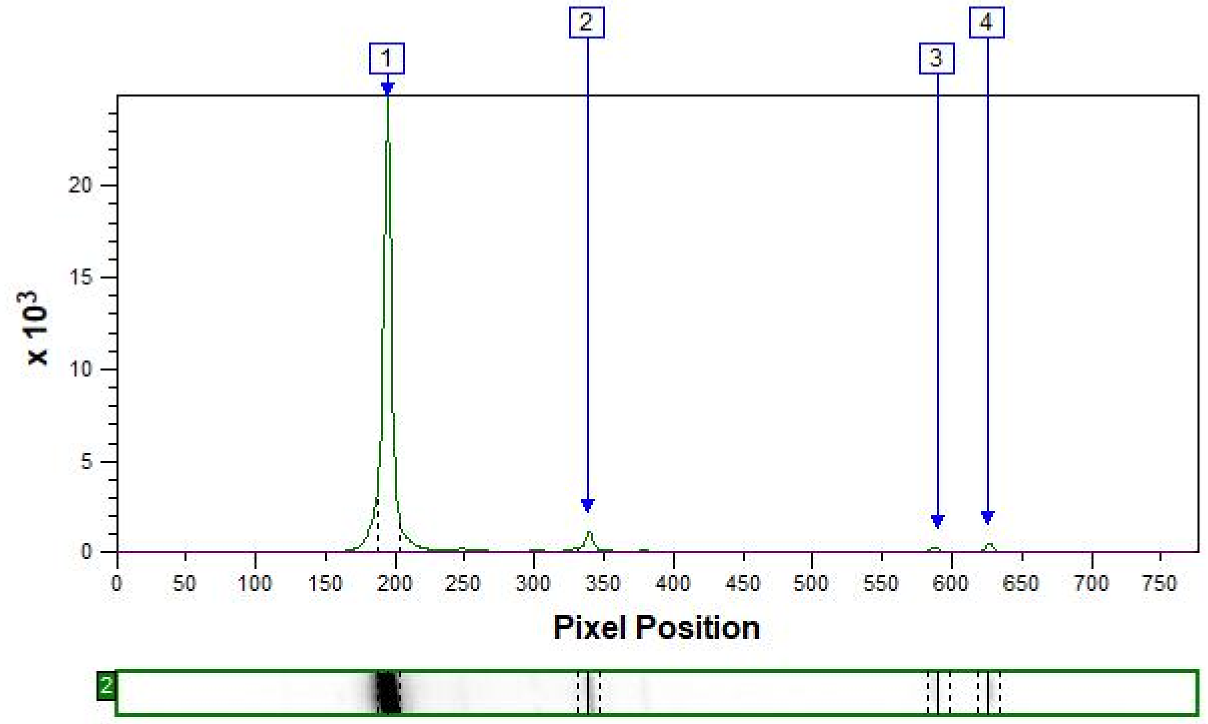

Band Table

Band No	Volume	Vol+BkGnd	Band %
1	5549625.26	5557304.75	91.945
2	287280.50	294973.94	4.760
3	83050.09	90846.26	1.376
4	115862.07	123627.79	1.920

IQTL Report version 8.1
Time of Report: 5:06 PM 10/23/2025 (Eastern Daylight Time)

ImageQuant TL

Lane Data Report: rw 2025_10_23 p8_p6b-[phosphor].tif, Lane 3

Summary

**Name:**	Lane 3

Background

**Background Type:**	Image Rectangle
**Rectangle:**	Left 429, Right 1125, Top 691, Bottom 783

Band Detection

**Automatic Detection:**	Yes
**Minimum Band Slope:**	182
**Median Filter:**	5
**Percentage Maximum Peak:**	0
**Maximum Peak Measure:**	Gel
**Edge Detection Method:**	Fixed Width
**Fixed Width pixels:**	17
**Band Positions Edited:**	Yes

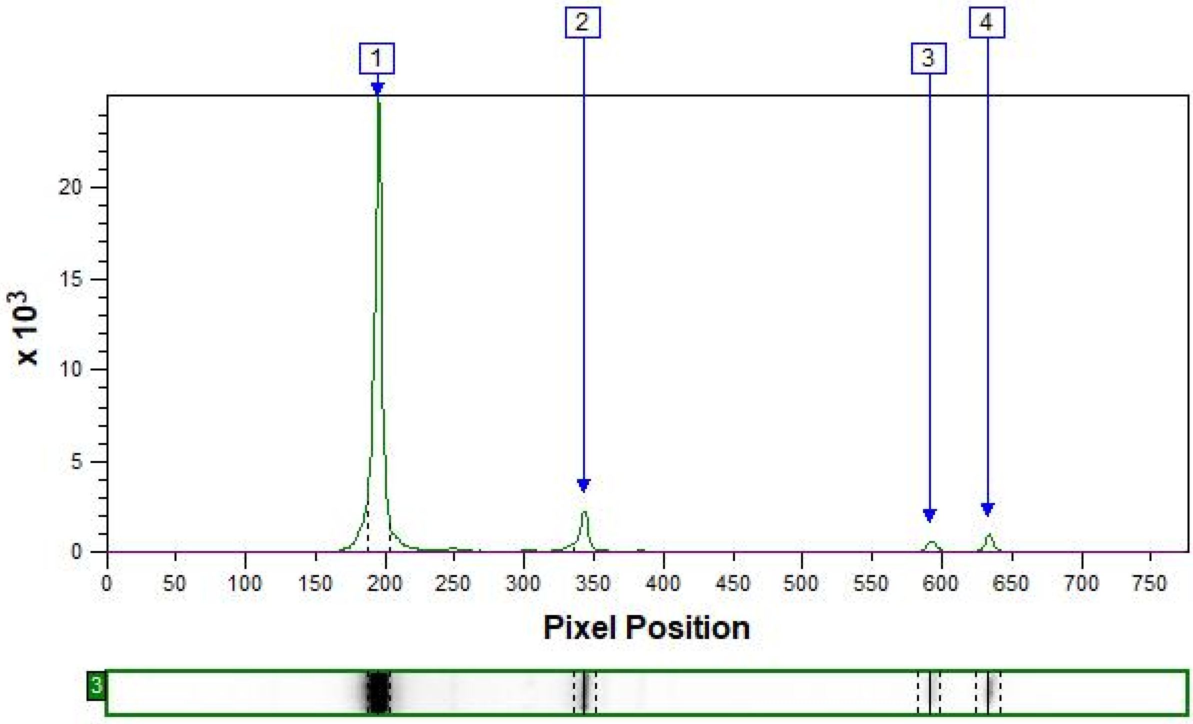

Band Table

Band No	Volume	Vol+BkGnd	Band %
1	5293038.00	5300718.00	84.962
2	535626.00	543306.00	8.598
3	167740.00	175420.00	2.693
4	233490.00	241170.00	3.748

ImageQuant TL

Lane Data Report: rw 2025_10_23 p8_p6b-[phosphor].tif, Lane 4

Summary

**Name:**	Lane 4

Background

**Background Type:**	Image Rectangle
**Rectangle:**	Left 429, Right 1125, Top 691, Bottom 783

Band Detection

**Automatic Detection:**	Yes
**Minimum Band Slope:**	182
**Median Filter:**	5
**Percentage Maximum Peak:**	0
**Maximum Peak Measure:**	Gel
**Edge Detection Method:**	Fixed Width
**Fixed Width pixels:**	17
**Band Positions Edited:**	Yes

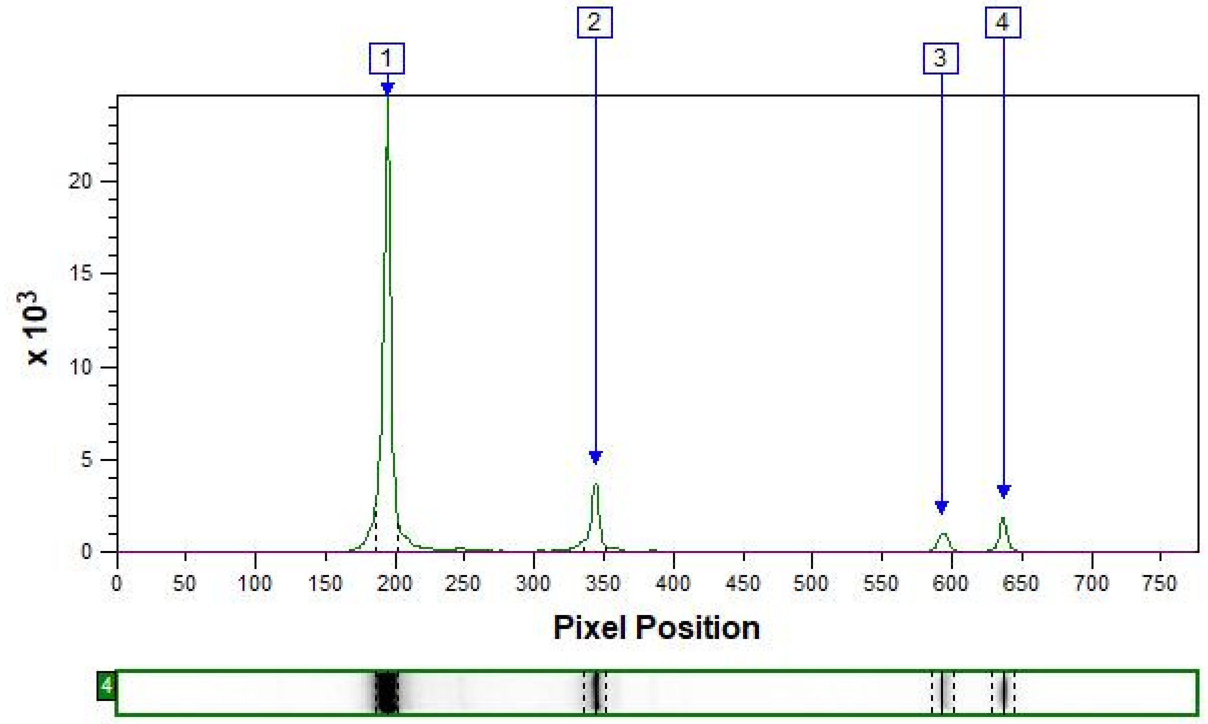

Band Table

Band No	Volume	Vol+BkGnd	Band %
1	5059110.00	5066790.00	76.660
2	843903.00	851583.00	12.787
3	305320.00	313000.00	4.626
4	391120.00	398800.00	5.927

Lane Data Report: rw 2025_10_23 p8_p6b-[phosphor].tif, Lane 5

Summary

**Name:**	Lane 5

Background

**Background Type:**	Image Rectangle
**Rectangle:**	Left 429, Right 1125, Top 691, Bottom 783

Band Detection

**Automatic Detection:**	Yes
**Minimum Band Slope:**	182
**Median Filter:**	5
**Percentage Maximum Peak:**	0
**Maximum Peak Measure:**	Gel
**Edge Detection Method:**	Fixed Width
**Fixed Width pixels:**	17
**Band Positions Edited:**	Yes

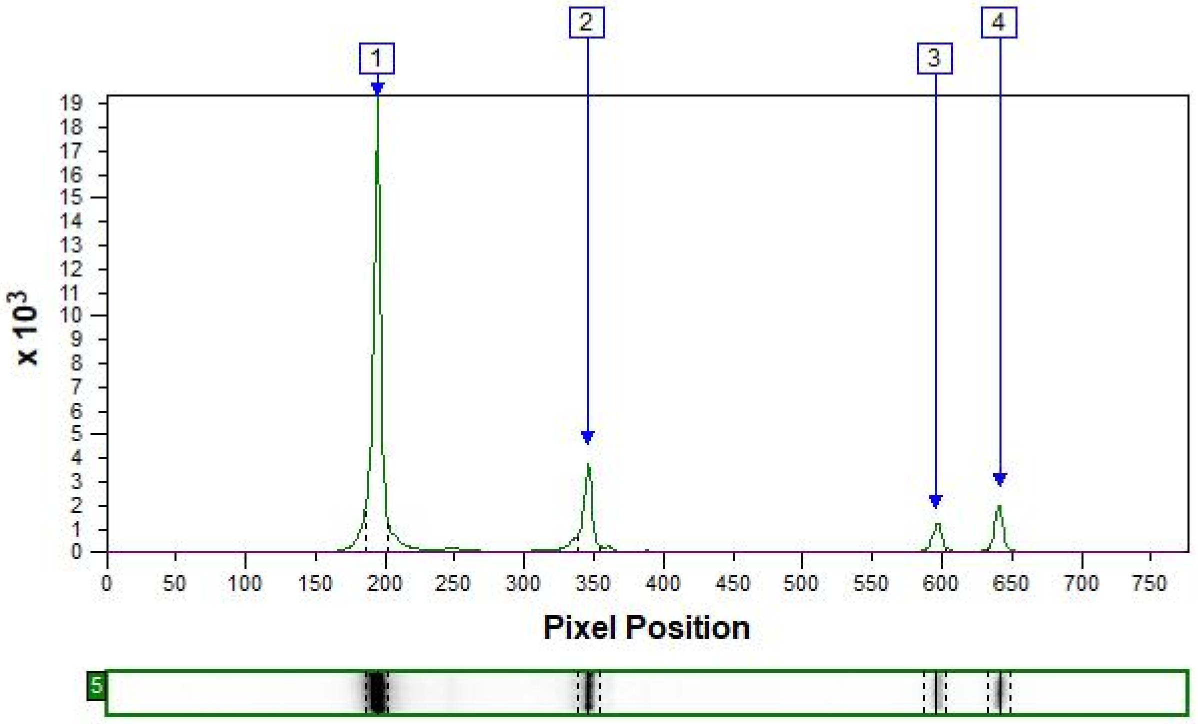

Band Table

Band No	Volume	Vol+BkGnd	Band %
1	3780012.00	3787692.00	69.842
2	876402.00	884082.00	16.193
3	322123.00	329803.00	5.952
4	433722.00	441402.00	8.014

ImageQuant TL

Lane Data Report: rw 2025_10_23 p8_p6b-[phosphor].tif, Lane 6

Summary

**Name:**	Lane 6

Background

**Background Type:**	Image Rectangle
**Rectangle:**	Left 429, Right 1125, Top 691, Bottom 783

Band Detection

**Automatic Detection:**	Yes
**Minimum Band Slope:**	182
**Median Filter:**	5
**Percentage Maximum Peak:**	0
**Maximum Peak Measure:**	Gel
**Edge Detection Method:**	Fixed Width
**Fixed Width pixels:**	17
**Band Positions Edited:**	Yes

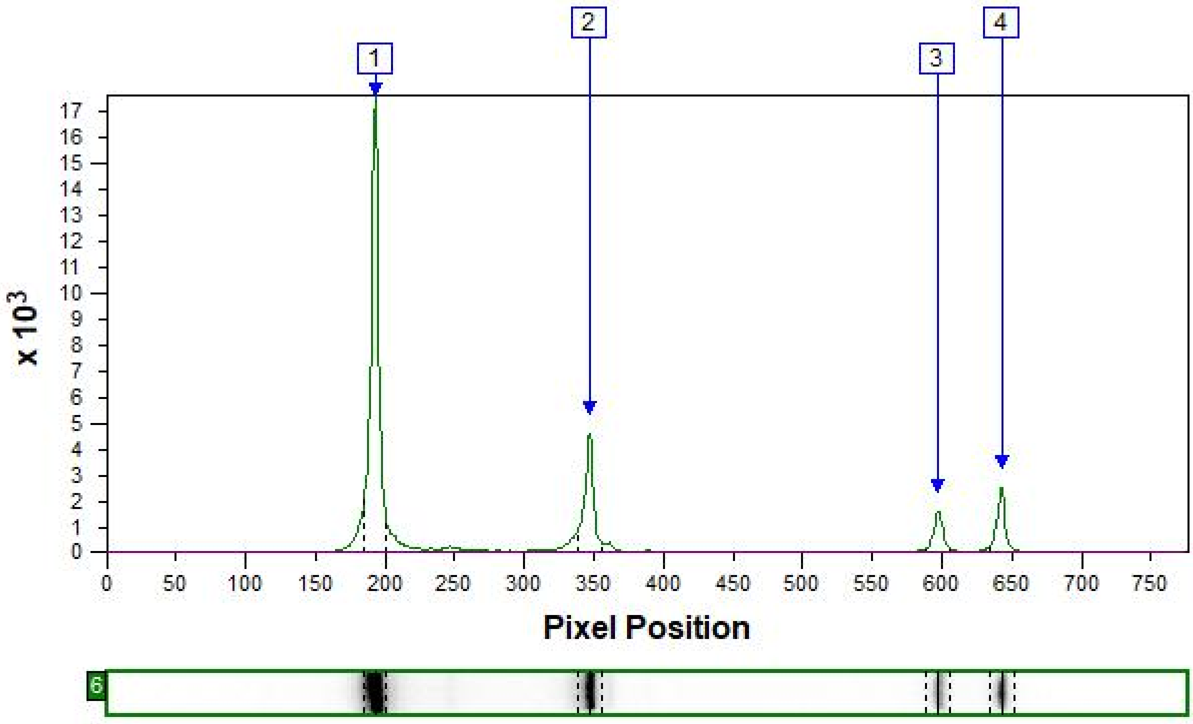

Band Table

Band No	Volume	Vol+BkGnd	Band %
1	3580455.00	3588135.00	64.022
2	1089509.00	1097189.00	19.481
3	400424.00	408104.00	7.160
4	522175.00	529855.00	9.337

Lane Data Report: rw 2025_10_23 p8_p6b-[phosphor].tif, Lane 7

Summary

**Name:**	Lane 7

Background

**Background Type:**	Image Rectangle
**Rectangle:**	Left 429, Right 1125, Top 691, Bottom 783

Band Detection

**Automatic Detection:**	Yes
**Minimum Band Slope:**	182
**Median Filter:**	5
**Percentage Maximum Peak:**	0
**Maximum Peak Measure:**	Gel
**Edge Detection Method:**	Fixed Width
**Fixed Width pixels:**	17
**Band Positions Edited:**	Yes

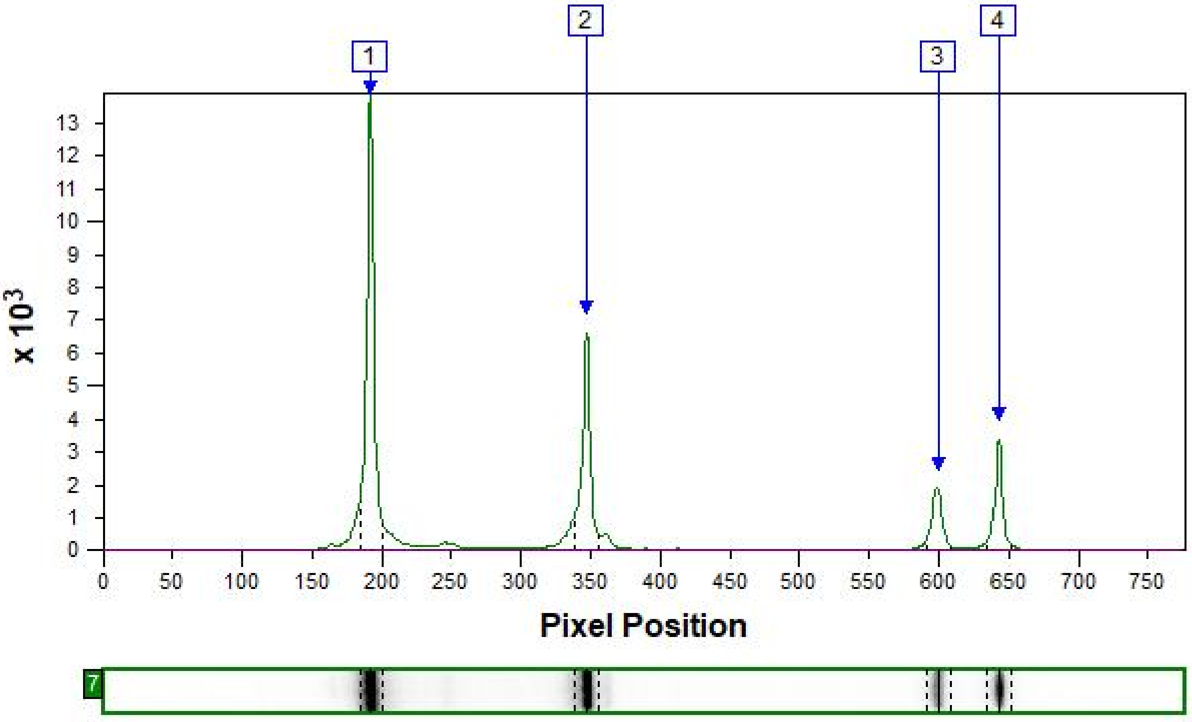

Band Table

Band No	Volume	Vol+BkGnd	Band %
1	2687322.00	2695002.00	50.046
2	1457154.00	1464834.00	27.137
3	532116.00	539796.00	9.910
4	693081.00	700761.00	12.907

IQTL Report version 8.1
Time of Report: 5:06 PM 10/23/2025 (Eastern Daylight Time)

ImageQuant TL

Lane Data Report: rw 2025_10_23 p8_p6b-[phosphor].tif, Lane 8

Summary

**Name:**	Lane 8

Background

**Background Type:**	Image Rectangle
**Rectangle:**	Left 429, Right 1125, Top 691, Bottom 783

Band Detection

**Automatic Detection:**	Yes
**Minimum Band Slope:**	182
**Median Filter:**	5
**Percentage Maximum Peak:**	0
**Maximum Peak Measure:**	Gel
**Edge Detection Method:**	Fixed Width
**Fixed Width pixels:**	17
**Band Positions Edited:**	No

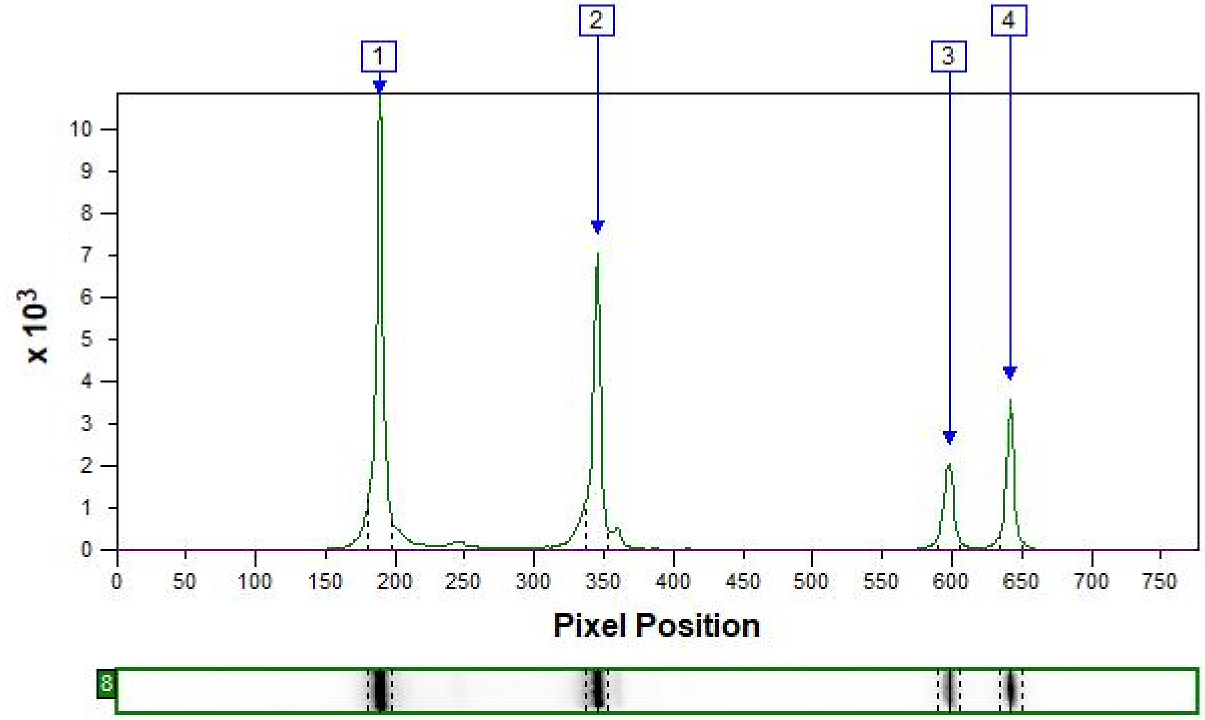

Band Table

Band No	Volume	Vol+BkGnd	Band %
1	2159051.00	2166731.00	43.409
2	1518209.00	1525889.00	30.524
3	571852.00	579532.00	11.497
4	724684.00	732364.00	14.570

ImageQuant TL

Lane Data Report: rw 2025_10_23 p8_p6b-[phosphor].tif, Lane 9

Summary

**Name:**	Lane 9

Background

**Background Type:**	Image Rectangle
**Rectangle:**	Left 429, Right 1125, Top 691, Bottom 783

Band Detection

**Automatic Detection:**	Yes
**Minimum Band Slope:**	182
**Median Filter:**	5
**Percentage Maximum Peak:**	0
**Maximum Peak Measure:**	Gel
**Edge Detection Method:**	Fixed Width
**Fixed Width pixels:**	17
**Band Positions Edited:**	Yes

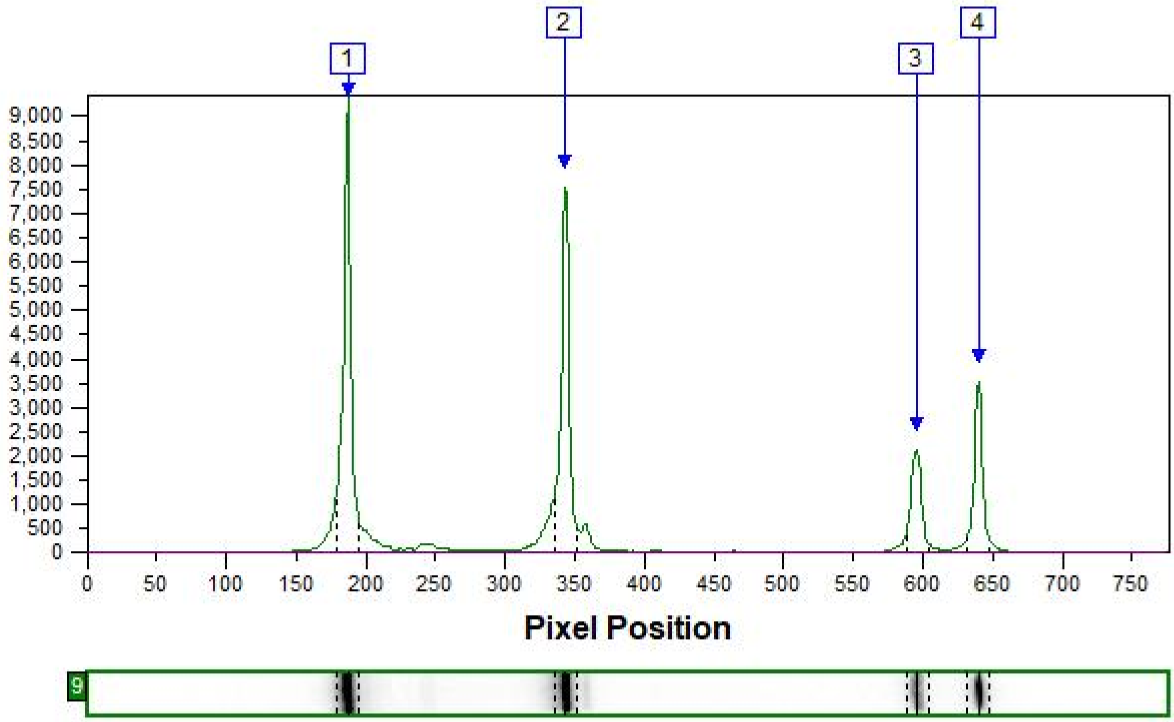

Band Table

Band No	Volume	Vol+BkGnd	Band %
1	1886984.00	1894664.00	39.180
2	1596728.00	1604408.00	33.153
3	596127.00	603807.00	12.377
4	736388.00	744068.00	15.290

ImageQuant TL

Lane Data Report: rw 2025_10_23 p8_p6b-[phosphor].tif, Lane 10

Summary

**Name:**	Lane 10

Background

**Background Type:**	Image Rectangle
**Rectangle:**	Left 429, Right 1125, Top 691, Bottom 783

Band Detection

**Automatic Detection:**	Yes
**Minimum Band Slope:**	182
**Median Filter:**	5
**Percentage Maximum Peak:**	0
**Maximum Peak Measure:**	Gel
**Edge Detection Method:**	Fixed Width
**Fixed Width pixels:**	17
**Band Positions Edited:**	No

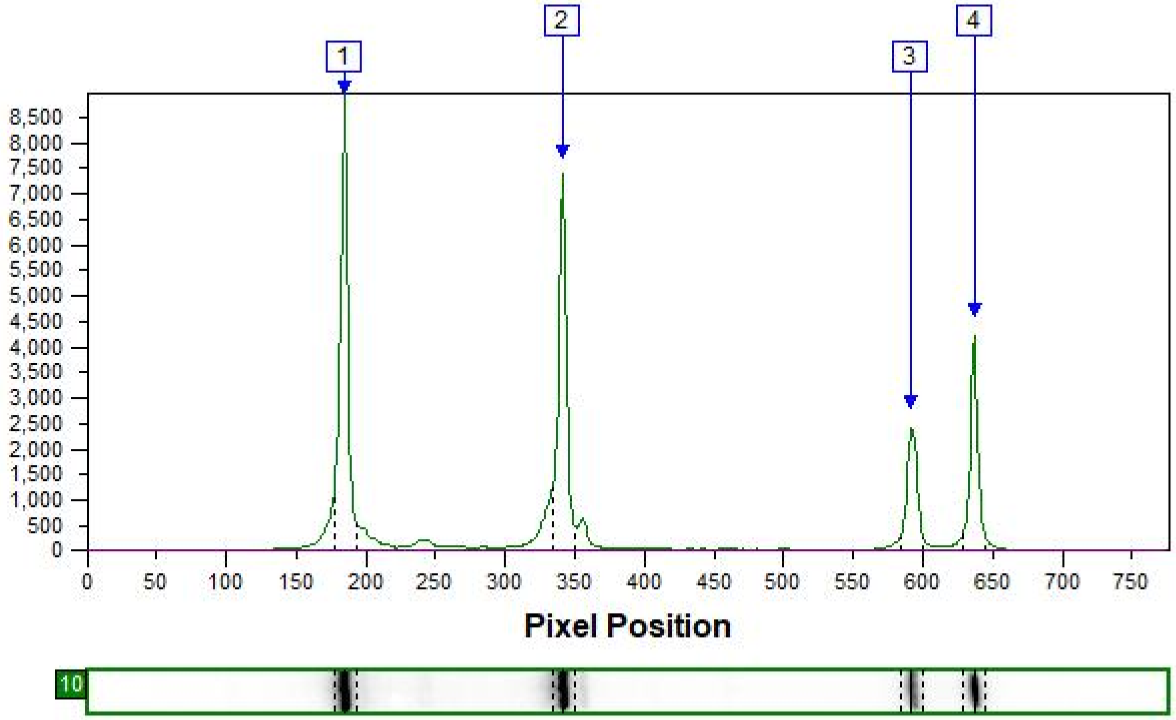

Band Table

Band No	Volume	Vol+BkGnd	Band %
1	1810563.00	1818243.00	36.936
2	1598140.00	1605820.00	32.602
3	659165.00	666845.00	13.447
4	834086.00	841766.00	17.015

ImageQuant TL

Lane Data Report: rw 2025_10_23 p8_p6b-[phosphor].tif, Lane 11

Summary

**Name:**	Lane 11

Background

**Background Type:**	Image Rectangle
**Rectangle:**	Left 429, Right 1125, Top 691, Bottom 783

Band Detection

**Automatic Detection:**	Yes
**Minimum Band Slope:**	182
**Median Filter:**	5
**Percentage Maximum Peak:**	0
**Maximum Peak Measure:**	Gel
**Edge Detection Method:**	Fixed Width
**Fixed Width pixels:**	17
**Band Positions Edited:**	Yes

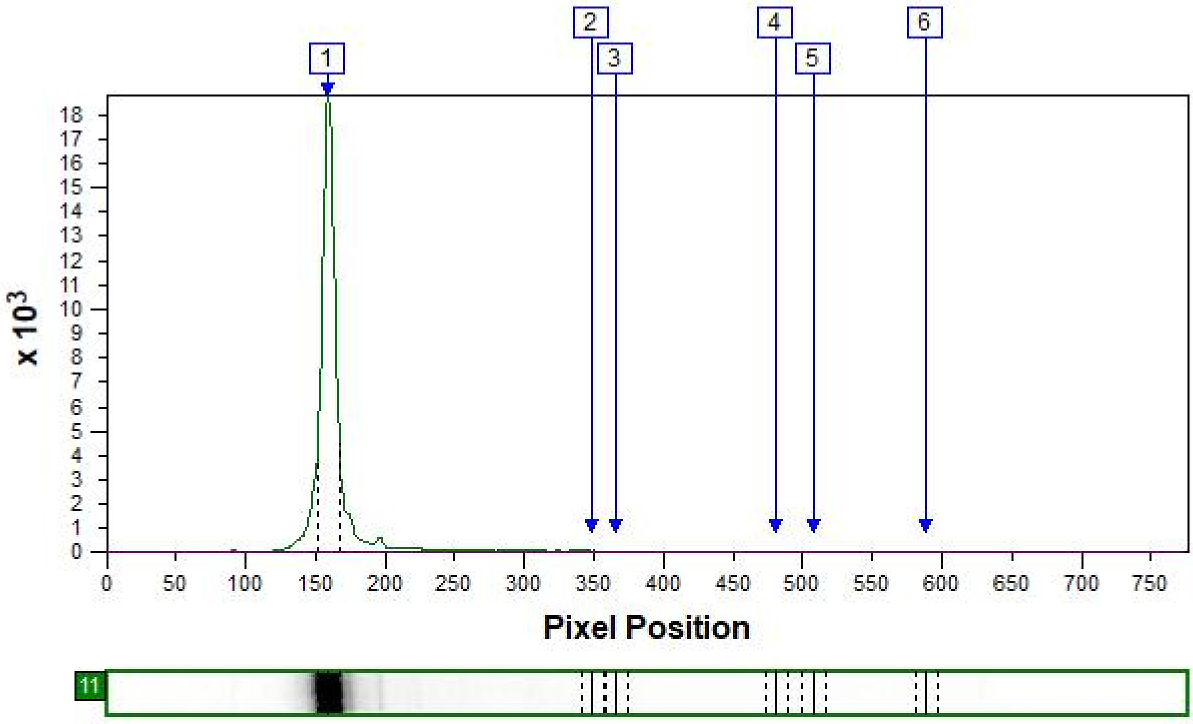

Band Table

Band No	Volume	Vol+BkGnd	Band %
1	6023084.00	6030764.00	98.507
2	35308.00	42988.00	0.577
3	25689.00	33369.00	0.420
4	13242.00	20922.00	0.217
5	8501.00	16181.00	0.139
6	8531.00	16211.00	0.140

IQTL Report version 8.1
Time of Report: 5:06 PM 10/23/2025 (Eastern Daylight Time)

ImageQuant TL

Lane Data Report: rw 2025_10_23 p8_p6b-[phosphor].tif, Lane 12

Summary

**Name:**	Lane 12

Background

**Background Type:**	Image Rectangle
**Rectangle:**	Left 429, Right 1125, Top 691, Bottom 783

Band Detection

**Automatic Detection:**	Yes
**Minimum Band Slope:**	182
**Median Filter:**	5
**Percentage Maximum Peak:**	0
**Maximum Peak Measure:**	Gel
**Edge Detection Method:**	Fixed Width
**Fixed Width pixels:**	17
**Band Positions Edited:**	Yes

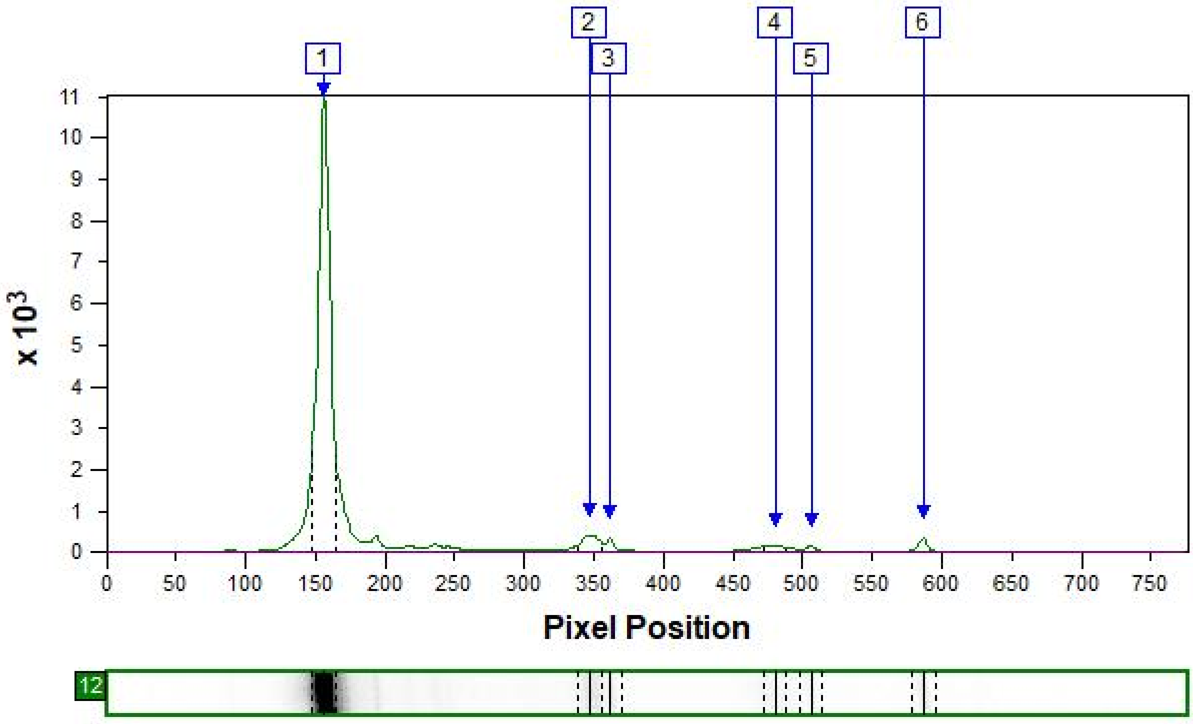

Band Table

Band No	Volume	Vol+BkGnd	Band %
1	3433586.00	3441266.00	87.817
2	172362.00	180042.00	4.408
3	94855.00	102055.00	2.426
4	75124.00	82804.00	1.921
5	48012.00	55692.00	1.228
6	86012.00	93692.00	2.200

ImageQuant TL

Lane Data Report: rw 2025_10_23 p8_p6b-[phosphor].tif, Lane 13

Summary

**Name:**	Lane 13

Background

**Background Type:**	Image Rectangle
**Rectangle:**	Left 429, Right 1125, Top 691, Bottom 783

Band Detection

**Automatic Detection:**	Yes
**Minimum Band Slope:**	182
**Median Filter:**	5
**Percentage Maximum Peak:**	0
**Maximum Peak Measure:**	Gel
**Edge Detection Method:**	Fixed Width
**Fixed Width pixels:**	17
**Band Positions Edited:**	Yes

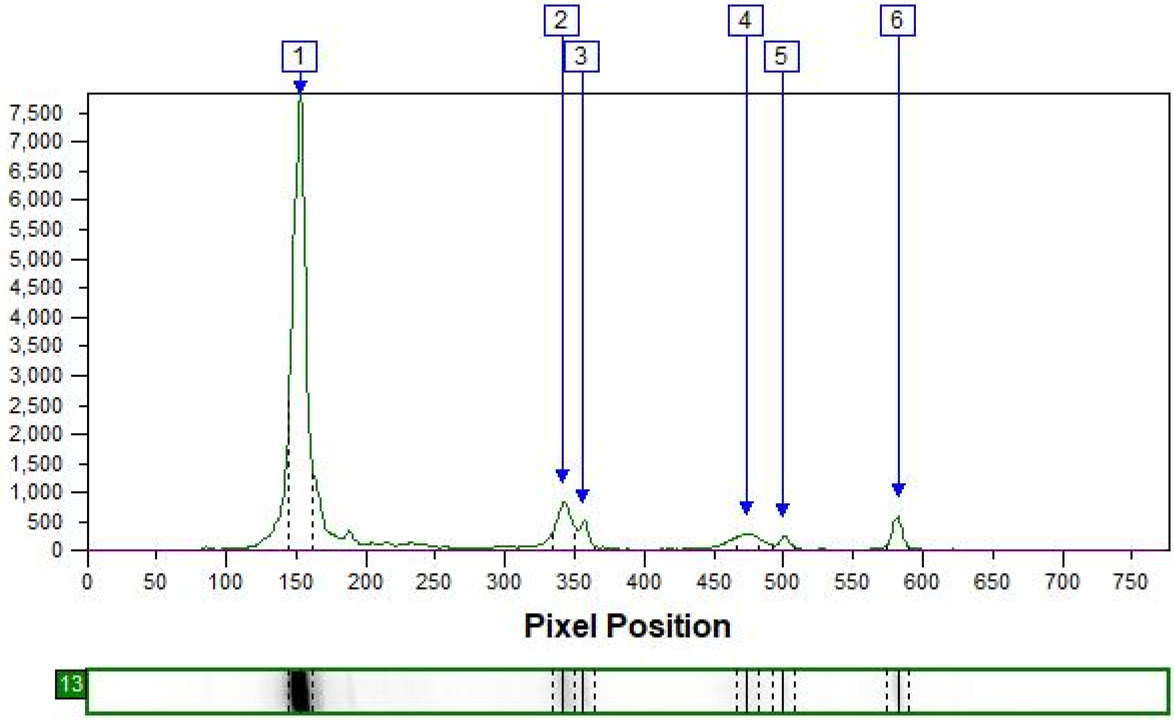

Band Table

Band No	Volume	Vol+BkGnd	Band %
1	2578186.00	2585866.00	75.720
2	306151.00	313831.00	8.992
3	156617.00	163337.00	4.600
4	131573.00	139253.00	3.864
5	74228.00	81908.00	2.180
6	158117.00	165797.00	4.644

Lane Data Report: rw 2025_10_23 p8_p6b-[phosphor].tif, Lane 14

Summary

**Name:**	Lane 14

Background

**Background Type:**	Image Rectangle
**Rectangle:**	Left 429, Right 1125, Top 691, Bottom 783

Band Detection

**Automatic Detection:**	Yes
**Minimum Band Slope:**	182
**Median Filter:**	5
**Percentage Maximum Peak:**	0
**Maximum Peak Measure:**	Gel
**Edge Detection Method:**	Fixed Width
**Fixed Width pixels:**	17
**Band Positions Edited:**	Yes

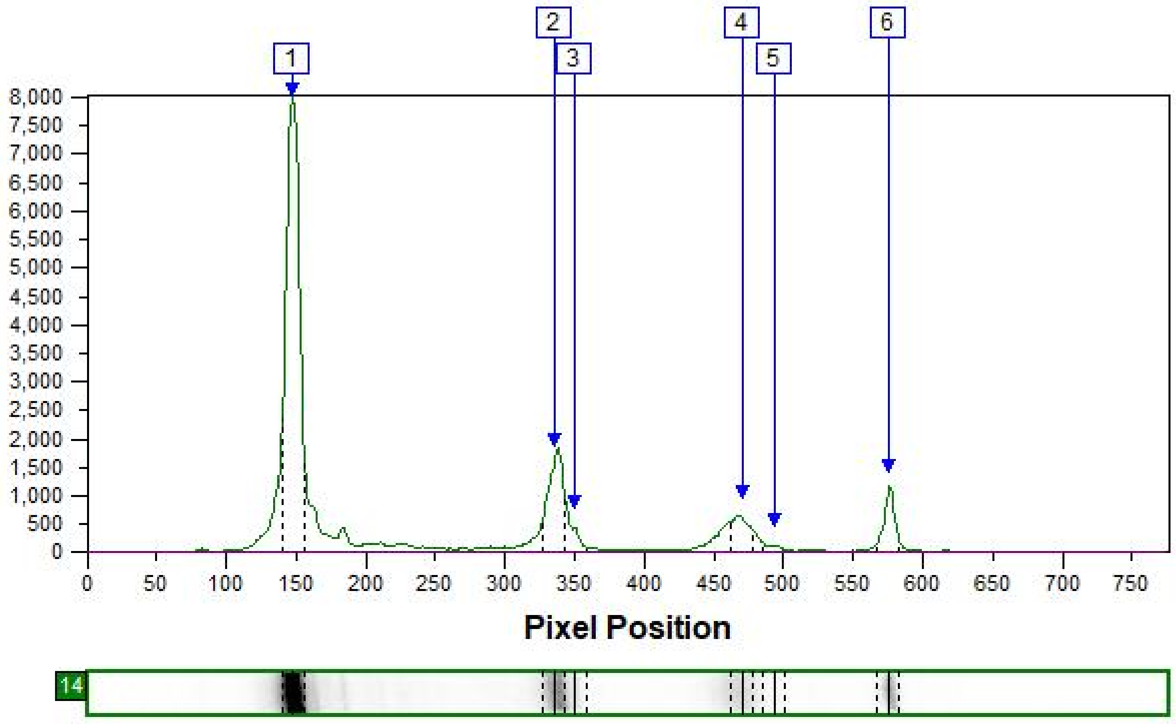

Band Table

Band No	Volume	Vol+BkGnd	Band %
1	2807655.00	2815335.00	64.688
2	674493.00	682173.00	15.540
3	202374.00	209574.00	4.663
4	281049.00	288729.00	6.475
5	55579.00	63259.00	1.281
6	319156.00	326836.00	7.353

ImageQuant TL

Lane Data Report: rw 2025_10_23 p8_p6b-[phosphor].tif, Lane 15

Summary

**Name:**	Lane 15

Background

**Background Type:**	Image Rectangle
**Rectangle:**	Left 429, Right 1125, Top 691, Bottom 783

Band Detection

**Automatic Detection:**	Yes
**Minimum Band Slope:**	182
**Median Filter:**	5
**Percentage Maximum Peak:**	0
**Maximum Peak Measure:**	Gel
**Edge Detection Method:**	Fixed Width
**Fixed Width pixels:**	17
**Band Positions Edited:**	Yes

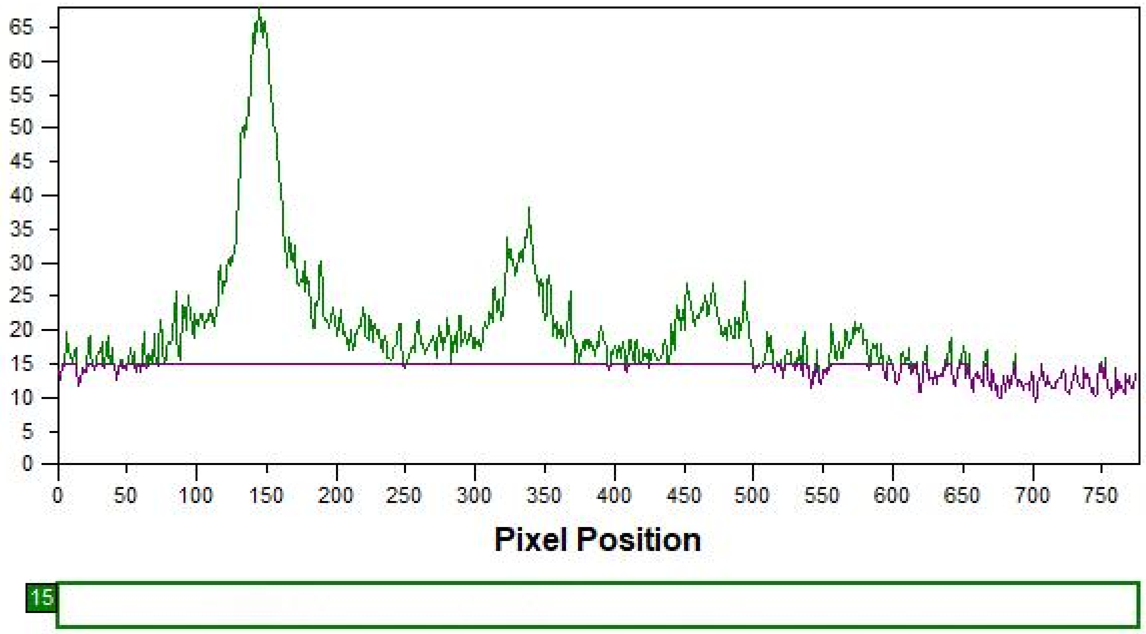

Band Table

Lane Data Report: rw 2025_10_23 p8_p6b-[phosphor].tif, Lane 16

Summary

**Name:**	Lane 16

Background

**Background Type:**	Image Rectangle
**Rectangle:**	Left 429, Right 1125, Top 691, Bottom 783

Band Detection

**Automatic Detection:**	Yes
**Minimum Band Slope:**	182
**Median Filter:**	5
**Percentage Maximum Peak:**	0
**Maximum Peak Measure:**	Gel
**Edge Detection Method:**	Fixed Width
**Fixed Width pixels:**	17
**Band Positions Edited:**	Yes

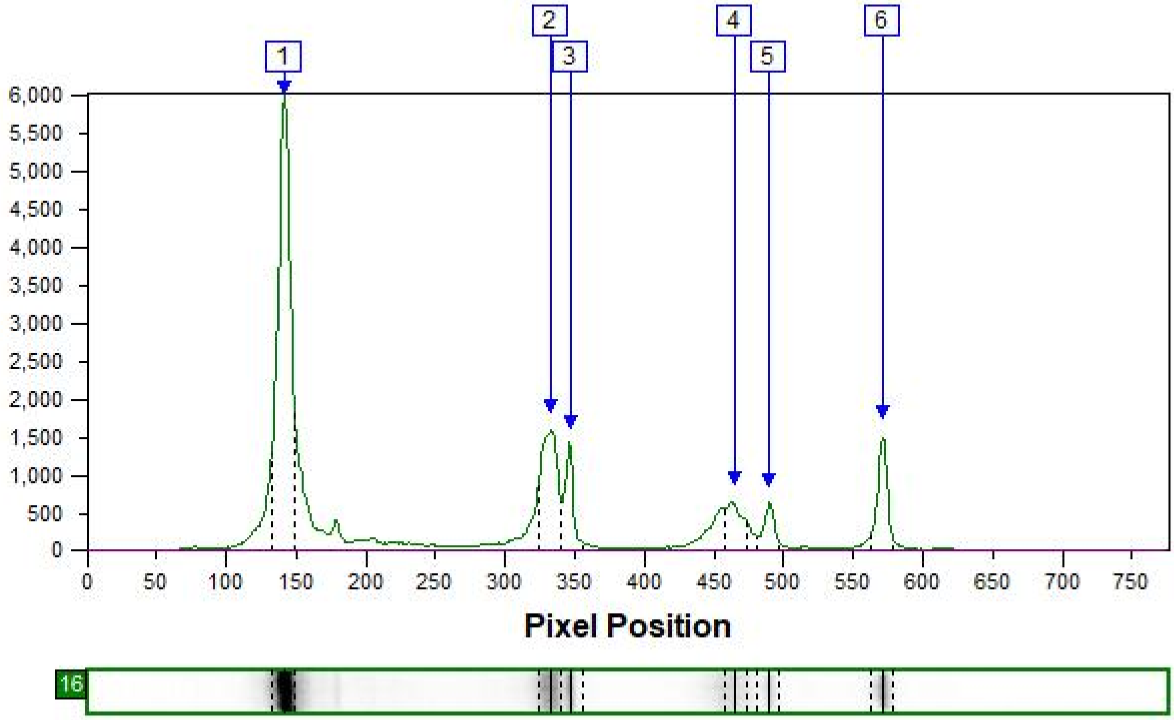

Band Table

Band No	Volume	Vol+BkGnd	Band %
1	1951608.00	1959288.00	51.530
2	671711.00	679391.00	17.736
3	331234.00	338434.00	8.746
4	270780.00	278460.00	7.150
5	178243.00	185923.00	4.706
6	383736.00	391416.00	10.132

ImageQuant TL

Lane Data Report: rw 2025_10_23 p8_p6b-[phosphor].tif, Lane 17

Summary

**Name:**	Lane 17

Background

**Background Type:**	Image Rectangle
**Rectangle:**	Left 429, Right 1125, Top 691, Bottom 783

Band Detection

**Automatic Detection:**	Yes
**Minimum Band Slope:**	182
**Median Filter:**	5
**Percentage Maximum Peak:**	0
**Maximum Peak Measure:**	Gel
**Edge Detection Method:**	Fixed Width
**Fixed Width pixels:**	17
**Band Positions Edited:**	Yes

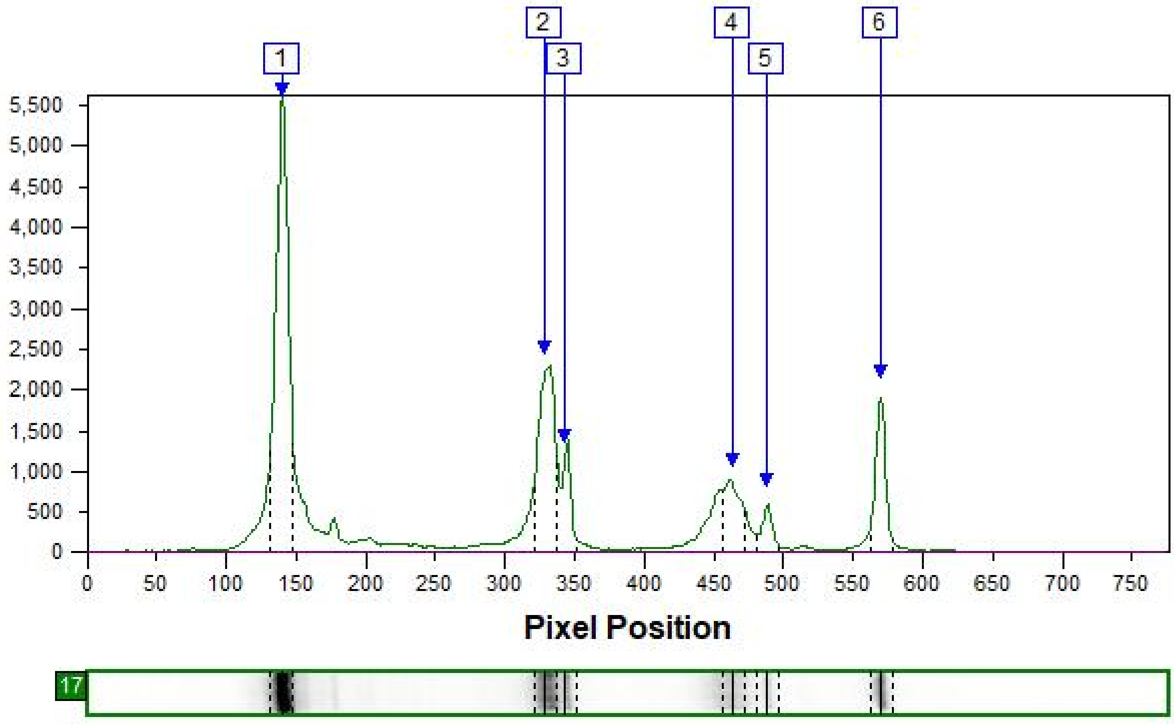

Band Table

Band No	Volume	Vol+BkGnd	Band %
1	1801290.00	1808970.00	43.100
2	932097.00	939777.00	22.303
3	390256.00	396976.00	9.338
4	386330.00	394010.00	9.244
5	170038.00	177718.00	4.069
6	499291.00	506971.00	11.947

IQTL Report version 8.1
Time of Report: 5:06 PM 10/23/2025 (Eastern Daylight Time)

ImageQuant TL

Lane Data Report: rw 2025_10_23 p8_p6b-[phosphor].tif, Lane 18

Summary

**Name:**	Lane 18

Background

**Background Type:**	Image Rectangle
**Rectangle:**	Left 429, Right 1125, Top 691, Bottom 783

Band Detection

**Automatic Detection:**	Yes
**Minimum Band Slope:**	182
**Median Filter:**	5
**Percentage Maximum Peak:**	0
**Maximum Peak Measure:**	Gel
**Edge Detection Method:**	Fixed Width
**Fixed Width pixels:**	17
**Band Positions Edited:**	Yes

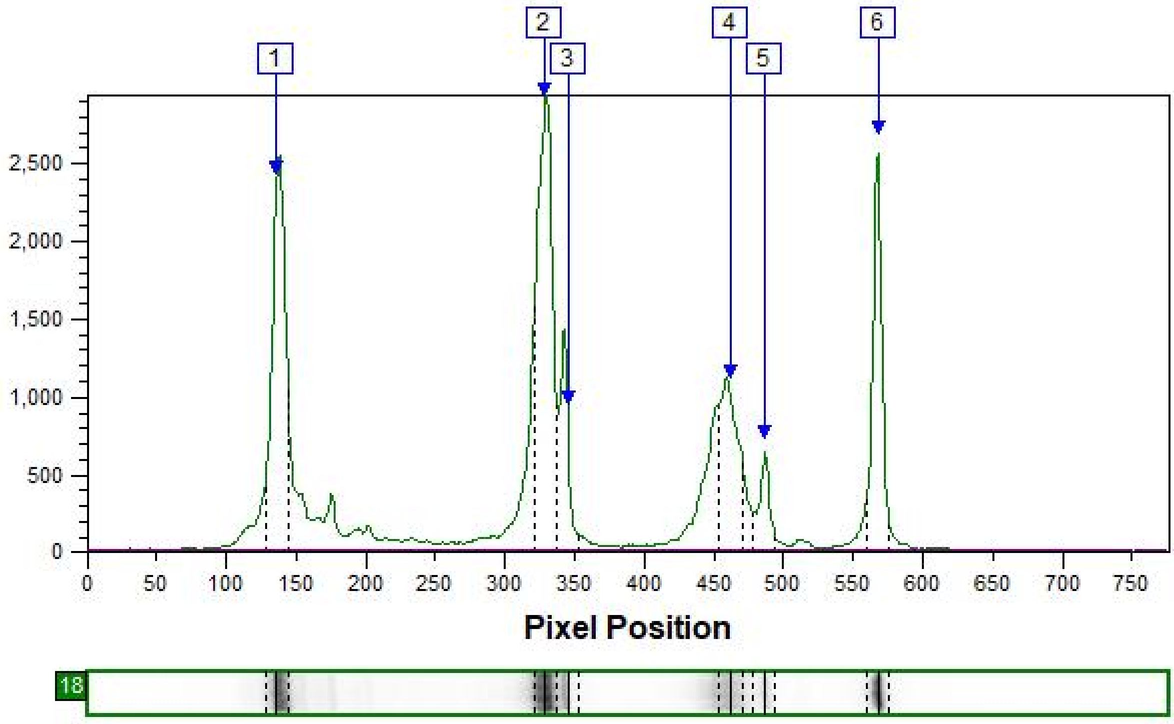

Band Table

Band No	Volume	Vol+BkGnd	Band %
1	808599.00	816279.00	22.567
2	1140253.00	1147933.00	31.823
3	360881.00	368561.00	10.072
4	471491.00	479171.00	13.159
5	178464.00	186144.00	4.981
6	623371.00	631051.00	17.398

Lane Data Report: rw 2025_10_23 p8_p6b-[phosphor].tif, Lane 19

Summary

**Name:**	Lane 19

Background

**Background Type:**	Image Rectangle
**Rectangle:**	Left 429, Right 1125, Top 691, Bottom 783

Band Detection

**Automatic Detection:**	Yes
**Minimum Band Slope:**	182
**Median Filter:**	5
**Percentage Maximum Peak:**	0
**Maximum Peak Measure:**	Gel
**Edge Detection Method:**	Fixed Width
**Fixed Width pixels:**	17
**Band Positions Edited:**	Yes

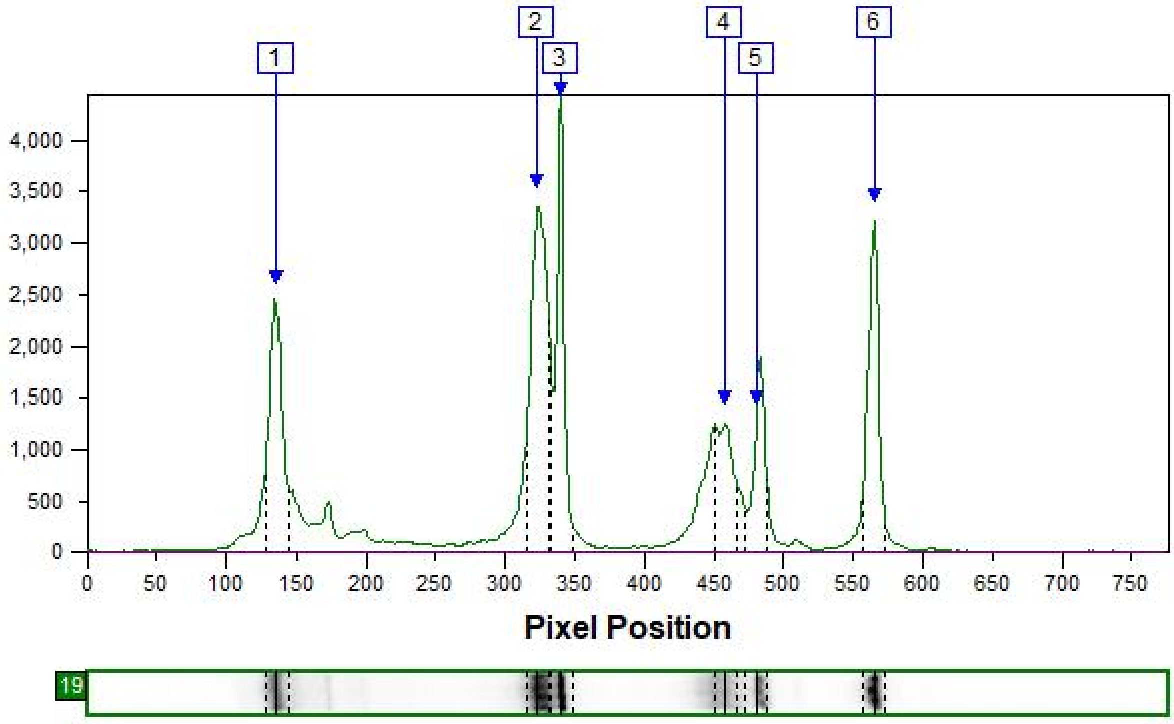

Band Table

Band No	Volume	Vol+BkGnd	Band %
1	791826.00	799506.00	15.410
2	1325595.00	1333275.00	25.798
3	1037769.00	1045449.00	20.197
4	558974.00	566654.00	10.879
5	520447.00	528127.00	10.129
6	903702.00	911382.00	17.588

ImageQuant TL

Lane Data Report: rw 2025_10_23 p8_p6b-[phosphor].tif, Lane 20

Summary

**Name:**	Lane 20

Background

**Background Type:**	Image Rectangle
**Rectangle:**	Left 429, Right 1125, Top 691, Bottom 783

Band Detection

**Automatic Detection:**	Yes
**Minimum Band Slope:**	182
**Median Filter:**	5
**Percentage Maximum Peak:**	0
**Maximum Peak Measure:**	Gel
**Edge Detection Method:**	Fixed Width
**Fixed Width pixels:**	17
**Band Positions Edited:**	Yes

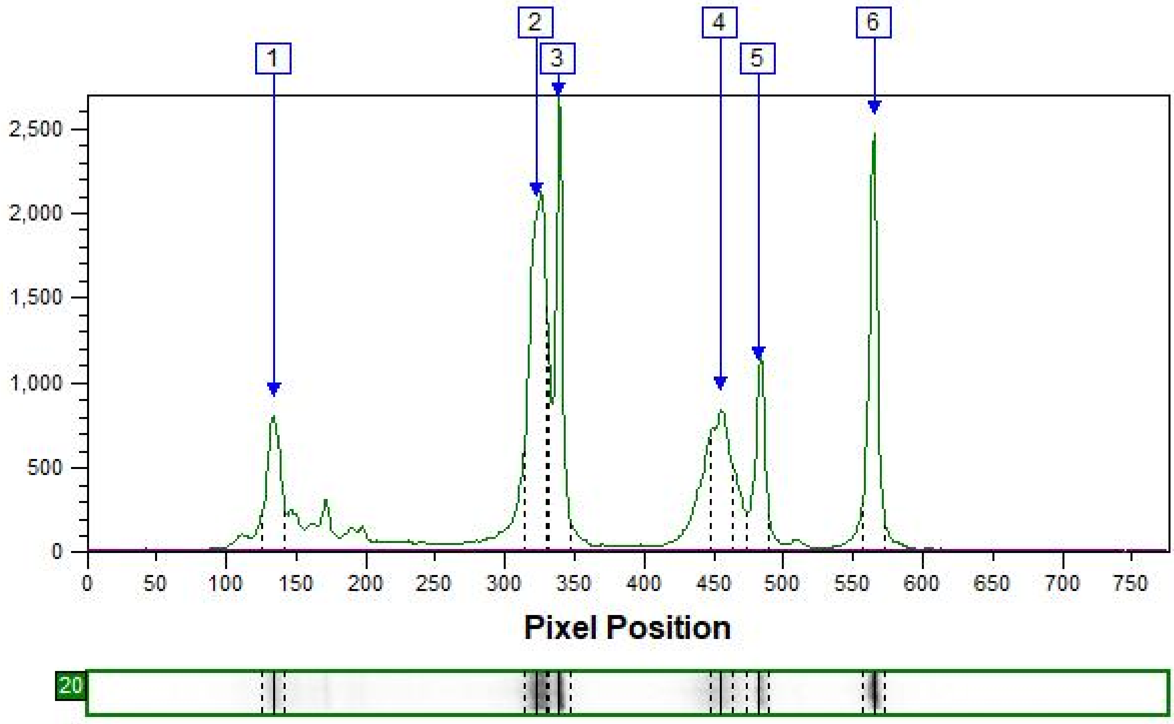

Band Table

Band No	Volume	Vol+BkGnd	Band %
1	272792.00	280472.00	9.175
2	827766.00	835446.00	27.840
3	623584.00	631264.00	20.973
4	367617.00	375297.00	12.364
5	308241.00	315921.00	10.367
6	573334.00	581014.00	19.283

Lane Data Report: rw 2025_10_23 p8_p6b-[phosphor].tif, Lane 21

Summary

**Name:**	Lane 21

Background

**Background Type:**	Image Rectangle
**Rectangle:**	Left 429, Right 1125, Top 691, Bottom 783

Band Detection

**Automatic Detection:**	Yes
**Minimum Band Slope:**	182
**Median Filter:**	5
**Percentage Maximum Peak:**	0
**Maximum Peak Measure:**	Gel
**Edge Detection Method:**	Fixed Width
**Fixed Width pixels:**	17
**Band Positions Edited:**	Yes

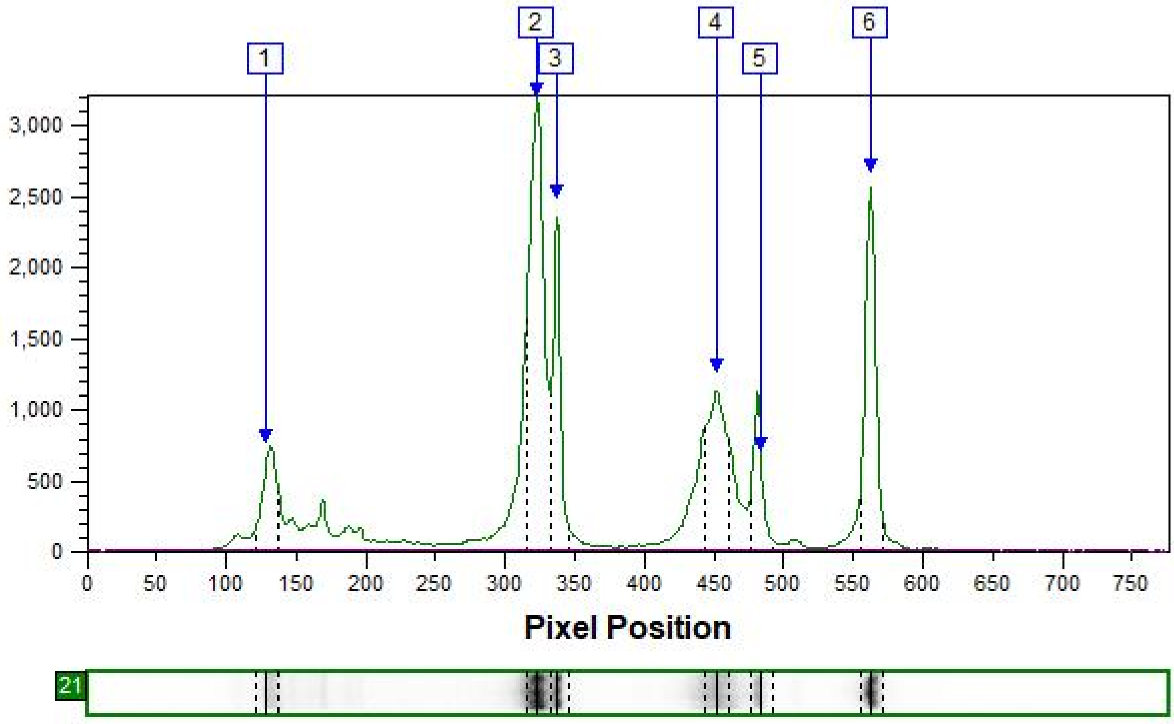

Band Table

Band No	Volume	Vol+BkGnd	Band %
1	246620.00	254300.00	7.092
2	1233305.00	1241465.00	35.465
3	519658.00	525898.00	14.943
4	497064.00	504744.00	14.294
5	271441.00	279121.00	7.806
6	709443.00	717123.00	20.401
